# PathwayMatcher: proteoform-centric network construction enables fine-granularity multiomics pathway mapping

**DOI:** 10.1093/gigascience/giz088

**Published:** 2019-07-30

**Authors:** Luis Francisco Hernández Sánchez, Bram Burger, Carlos Horro, Antonio Fabregat, Stefan Johansson, Pål Rasmus Njølstad, Harald Barsnes, Henning Hermjakob, Marc Vaudel

**Affiliations:** 1 K.G. Jebsen Center for Diabetes Research, Department of Clinical Science, University of Bergen, Children's Hospital, Haukeland University Hospital, 5021 Bergen, Norway; 2 Center for Medical Genetics and Molecular Medicine, Haukeland University Hospital, P.O Box 1400, 5021 Bergen, Norway; 3 European Molecular Biology Laboratory, European Bioinformatics Institute (EMBL-EBI), Wellcome Genome Campus, Hinxton, Cambridge, CB10 1SD, United Kingdom; 4 Proteomics Unit, Department of Biomedicine, University of Bergen, Postbox 7804, 5020 Bergen, Norway; 5 Computational Biology Unit, Department of Informatics, University of Bergen, P.O. Box 7803, 5020 Bergen, Norway; 6 Department of Pediatrics, Haukeland University Hospital, 5021 Bergen, Norway; 7 Beijing Proteome Research Center, National Center for Protein Sciences Beijing, No. 38, Life Science Park Road, Changping District, 102206 Beijing, China

**Keywords:** pathway, posttranslational modification, network, proteoform

## Abstract

**Background:**

Mapping biomedical data to functional knowledge is an essential task in bioinformatics and can be achieved by querying identifiers (*e.g*., gene sets) in pathway knowledge bases. However, the isoform and posttranslational modification states of proteins are lost when converting input and pathways into gene-centric lists.

**Findings:**

Based on the Reactome knowledge base, we built a network of protein-protein interactions accounting for the documented isoform and modification statuses of proteins. We then implemented a command line application called PathwayMatcher (github.com/PathwayAnalysisPlatform/PathwayMatcher) to query this network. PathwayMatcher supports multiple types of omics data as input and outputs the possibly affected biochemical reactions, subnetworks, and pathways.

**Conclusions:**

PathwayMatcher enables refining the network representation of pathways by including proteoforms defined as protein isoforms with posttranslational modifications. The specificity of pathway analyses is hence adapted to different levels of granularity, and it becomes possible to distinguish interactions between different forms of the same protein.

## Findings

In biomedicine, molecular pathways are used to infer the mechanisms underlying disease conditions and identify potential drug targets. Pathways are composed of series of biochemical reactions, of which the main participants are proteins, that together form a complex biological network. Proteins can be found in various forms, referred to as proteoforms [1]. The different proteoforms that can be obtained from the same gene/protein depend on the individual genetic profiles, on sequence cleavage and folding, and on posttranslational modification (PTM) states [2]. Proteoforms can carry PTMs at specific sites, conferring each proteoform unique structure and properties [2]. Notably, many pathway reactions can only occur if all or some of the proteins involved are in specific posttranslational states.

However, when analyzing omics data, both input and pathways are summarized in a gene- or protein-centric manner, meaning that the different proteoforms and their reactions are grouped by gene name or protein accession number, and the fine-grained structure of the pathways is lost. One can therefore anticipate that proteoform-centric networks provide a rich new paradigm to study biological systems. But while gene networks have proven their ability to identify genes associated with diseases [3], networks of finer granularity remain largely unexplored.

Here, we present PathwayMatcher, an open-source standalone application that considers the isoform and PTM status when building protein networks and mapping omics data to pathways from the Reactome database. Reactome [4] is an open-source curated knowledge base consolidating documented biochemical reactions categorized in hierarchical pathways and notably includes isoform and PTM information for the proteins participating in reactions and pathways.

**Figure 1.**
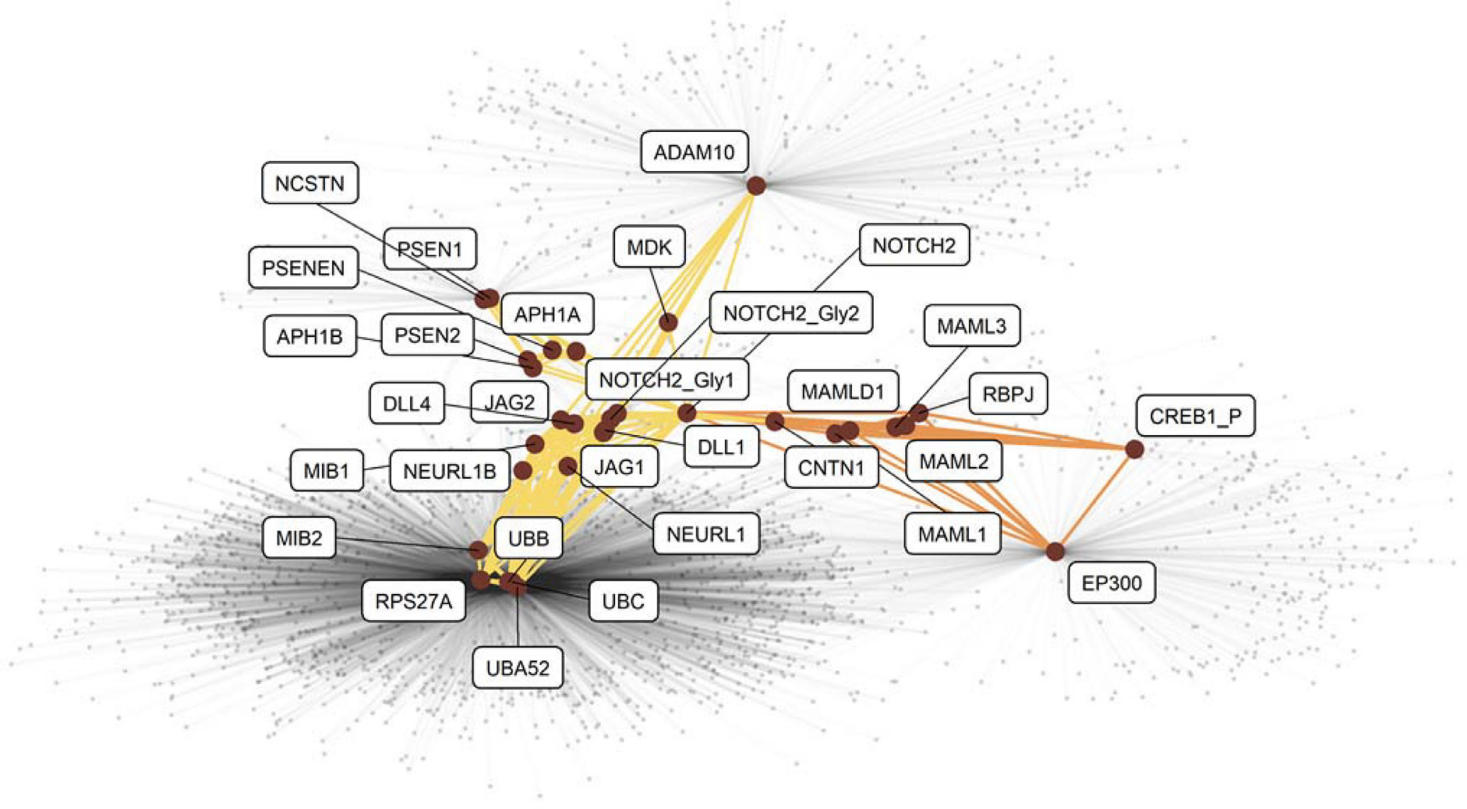
Graph representation of the *Signaling by NOTCH2* pathway as extracted from the Reactome database. Participating proteins are displayed as large dark red dots labeled with their canonical gene name. Posttranslational modifications (PTMs) are indicated with suffixes in the label. A connection between two dots indicates a documented interaction between the two proteins in the given pathway. Connections belonging to the subpathways *NOTCH2 intracellular domain regulate transcription* and *NOTCH2 activation and transmission of signal to the nucleus* are displayed in orange and yellow, respectively. The interactions involving these proteins in other pathways are displayed with light gray connections in the background.

As an example of the complexity of hierarchical pathway information, we provide a graph representation of *Signaling by NOTCH2* from Reactome (Fig. 1). This pathway is a subpathway of the pathways *Signaling by NOTCH* and *Signal Transduction*. It is composed of two subpathways (*NOTCH2 Intracellular Domain Regulates Transcription* and *NOTCH2 Activation and Transmission of Signal to the Nucleus*), comprising 32 and 54 reactions, yielding 28 and 141 edges, respectively. The 31 participants of the *Signaling by NOTCH2* pathway are also involved in reactions in other pathways, between themselves and with 2,055 other proteins, resulting in 6,525 external edges. Note that in this pathway, Cyclic AMP-responsive element-binding protein 1 (coded by *CREB1*) is phosphorylated at position 46 (labeled as *CERB1_P* in Fig. 1) and Neurogenic locus notch homolog protein 2 (coded by *NOTCH2*) is found in 3 forms (unmodified and with two combinations of glycosylation, labeled as NOTCH2, NOTCH2_Gly1, and NOTCH2_Gly2, respectively).

**Figure 2.**
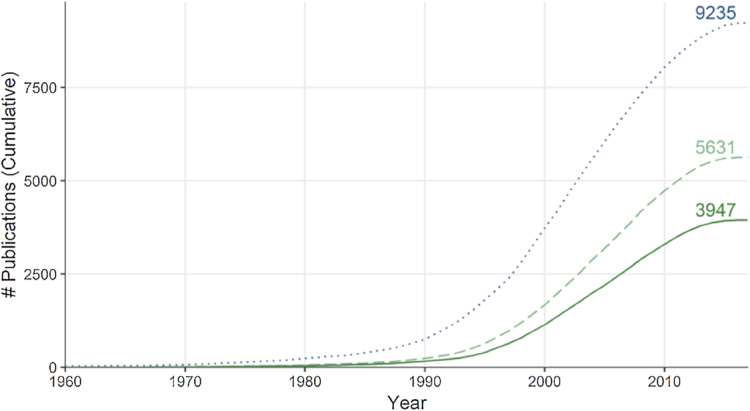
The cumulative number of publications indexed in Reactome documenting at least one reaction between two proteins with PTMs (solid dark green line), between one protein with PTMs and one without (dashed light green line), and two proteins without PTMs (dotted blue line), counting all publications with a year earlier than or equal to the *x*-axis value. The number of publications in each category at time of writing is indicated to the right.

The amount of information available on reactions involving modified proteins has dramatically increased during the past two decades (Fig.   2), with 3,947 and 5,631 publications indexed in Reactome (version 64 at time of writing) describing at least one reaction between modified proteins or between a modified and an unmodified protein, respectively. To harness this vast amount of knowledge, we built a network representation of pathways that we refer to as *proteoform-centric*, where protein isoforms with different sets of PTMs are represented with different nodes, in contrast to *gene-centric* networks, where one node is used per gene name or protein accession. In this representation, two proteoforms are connected if they participate in the same reaction. Note that proteoforms can participate in reactions both individually and as part of a set or complex. Furthermore, they can have four different roles: input, output, catalyst, or regulator.

**Figure 3.**
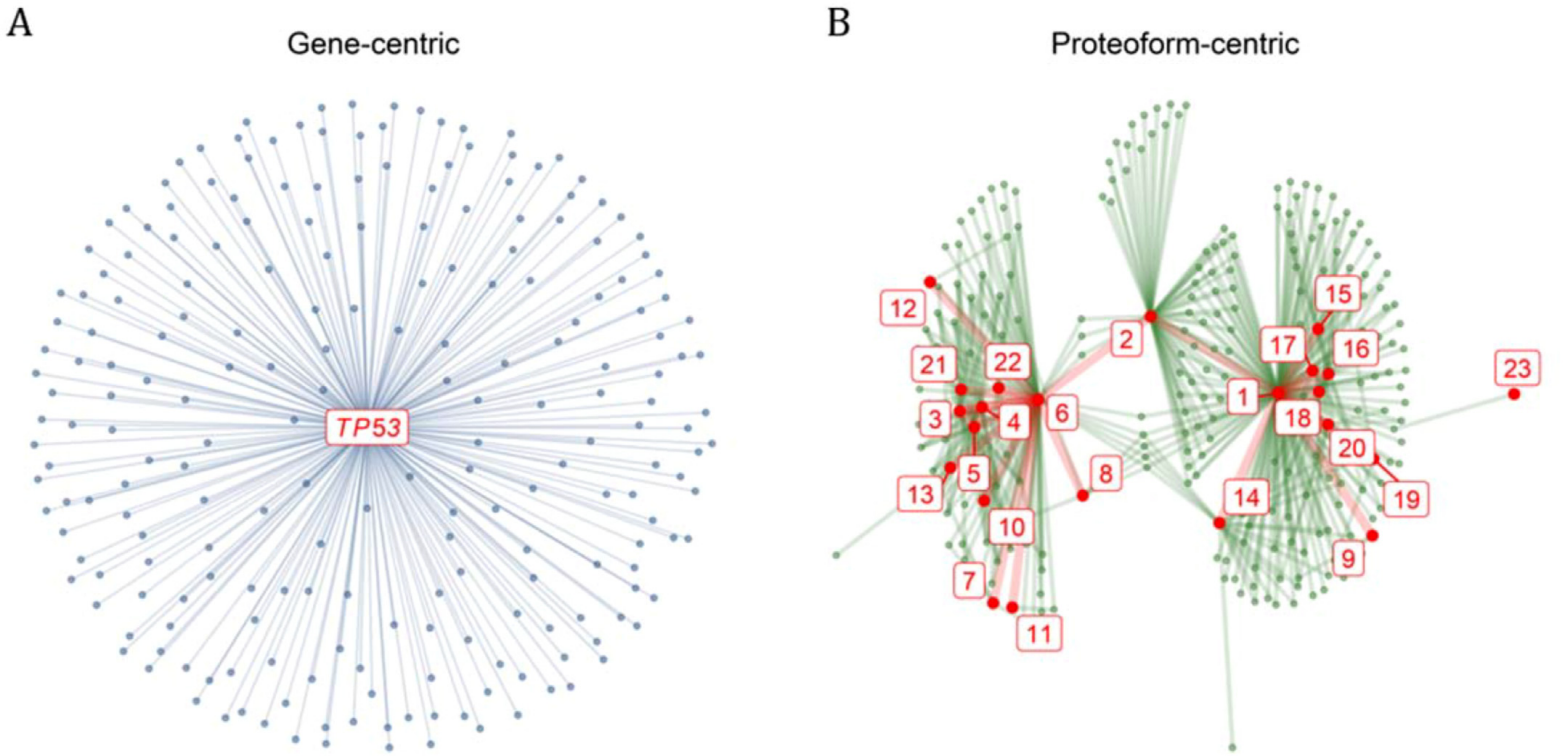
Gene-centric versus proteoform-centric representation. (A) Graph representation of the genes involved in reactions (through their corresponding proteins) with (the corresponding proteins of) *TP53*, with a single node per gene. *TP53* is represented with a red label at the center and genes coding proteins involved in reactions with *TP53* are represented with smaller blue dots at the periphery connected to the *TP53* gene with blue lines. (B) Graph representation of the proteins involved in a reaction with gene products of *TP53*, distinguishing isoforms and posttranslationally modified proteins as different proteoforms. The proteoforms coded by *TP53* and the proteoforms involved in a reaction with them are represented with large red and small green dots, respectively. The proteoforms coded by *TP53* are numbered according to Table 1. The connections between proteoforms coded by *TP53* are displayed with thick red lines and connections with other proteoforms with thin green lines.

The fundamental difference between gene- and proteoform-centric networks is illustrated in Fig. 3, showing the graph representation of interactions with the protein *cellular tumor antigen p53* (P04637) from the *TP53* gene. In a gene-centric paradigm (Fig. 3A), 221 nodes are connected to a single node, making 220 connections; while in a proteoform-centric network (Fig. 3B), 227 proteoforms connect to 23 proteoforms coded by *TP53*, making 414 connections. Note that the proteoforms coded by *TP53* are themselves involved in reactions, making 24 *TP53-TP53* connections. In this example, the proteoform-centric network thus presents more nodes and connections than the gene-centric network, with visible structural differences in the network organization. We hypothesize that the proteoform-centric network paradigm depicted in Fig. 3B provides a rich map that will enable navigating biomedical knowledge to a higher level of detail, to better assess the effect of perturbations and identify drug targets more specifically.

**Figure 4.**
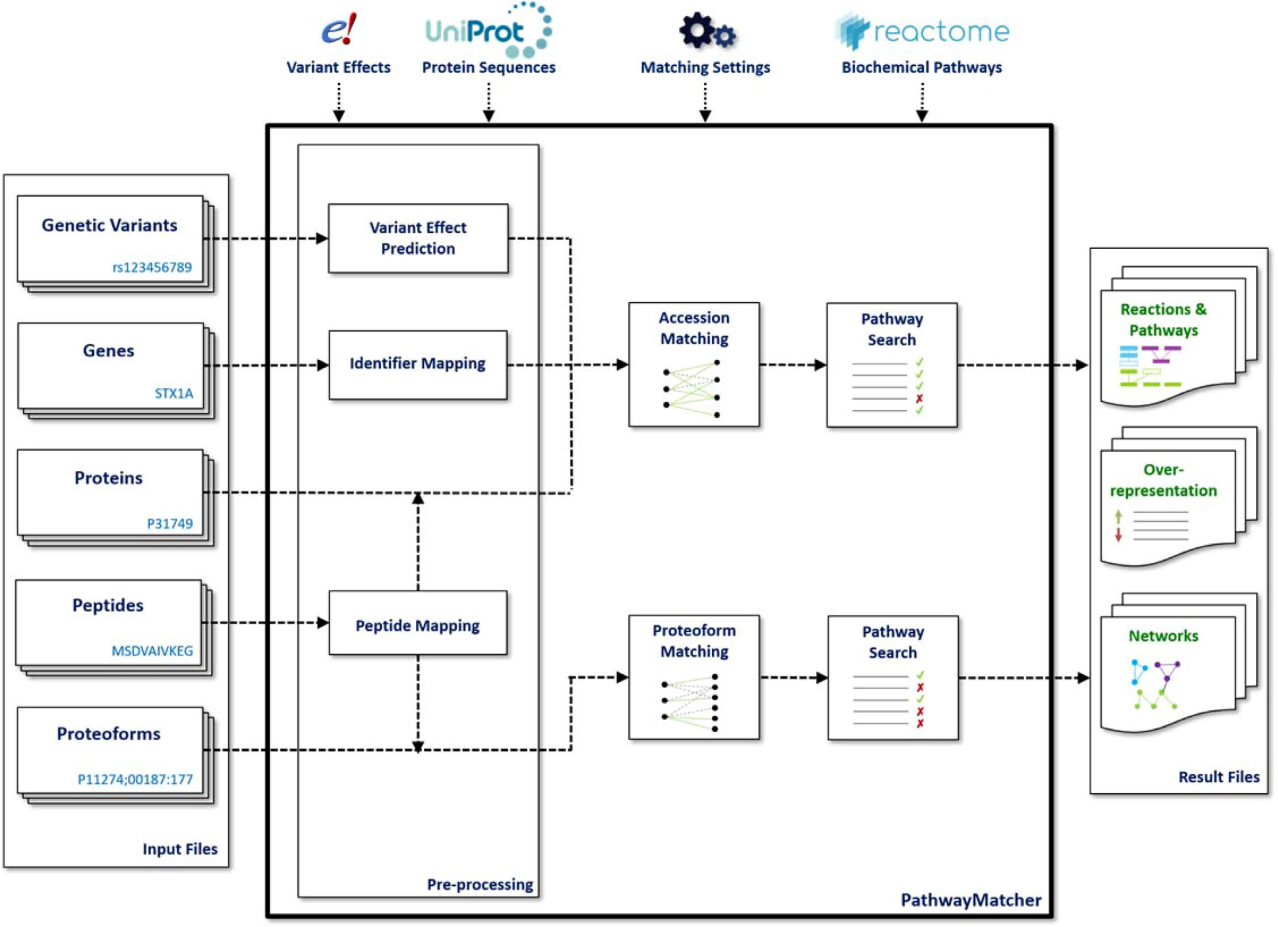
Schematic representation of the PathwayMatcher matching procedure. Input of various types is modelized as sets of proteins or proteoforms based on the annotation of isoforms and PTMs. Proteins and proteoforms are then mapped to Reactome based on user settings. Matched reactions and pathways, the results of an overrepresentation analysis, and subnetworks generated from the input are exported as text files.

PathwayMatcher allows the user to tune the granularity of the network representation of pathways by representing nodes as (i) gene names, (ii) protein accession numbers, or (iii) proteoforms and supports the mapping of multiple types of omics data: (i) genetic variants, (ii) genes, (iii) proteins, (iv) peptides, and (v) proteoforms. Genetic variants are mapped to proteins using the Ensembl Variant Effect Predictor [5], gene names are mapped to proteins using the UniProt identifier mapping [6], and peptides are mapped to proteins using PeptideMapper [7]. If a peptide maps to different proteins, all possible proteins are considered for the search and protein inference must be conducted *a posteriori* [8]. If peptides are modified, they are mapped to the proteoforms presenting compatible PTM sets. Proteins are mapped to the pathway network using their accession, while proteoforms are mapped by comparing their protein accession, isoform number, and PTM set. A schematic representation of the PathwayMatcher matching procedure is shown in Fig. 4. More details on the mapping procedure, formats, and settings can be found in the Methods section and in the online documentation (github.com/PathwayAnalysisPlatform/PathwayMatcher/wiki). For more information on how the pathway representation is constructed from the different external resources, please consult the Methods section and the online documentation (github.com/pathwayanalysisplatform/pathwaymatcher/tree/master/src/main/java/extractor).

PathwayMatcher produces three types of output: (i) the result of the matching, listing all possible reactions and pathways linked to the input; (ii) the results of an overrepresentation analysis; and (iii) networks in relationship with the input. The overrepresentation analysis is performed on the pathways matching and follows the first generation of pathway analysis methods [9], *i.e*., a *P*-value for each pathway in the reference database is calculated using a binomial distribution followed by Benjamini-Hochberg correction [10] (in a similar way as performed by the Reactome online analysis tool [4]). If the input can be mapped to proteoforms, the overrepresentation analysis is conducted using a proteoform-centric representation of pathways, using proteins otherwise. The exported networks represent the internal and external connections that can be drawn from the input, where internal connections connect two nodes from the input list, and external connections connect one node from the input list to any node not in the input. The user can select to export these networks using nodes defined as genes, proteins, or proteoforms. Connections between nodes in the network are annotated with information on whether they participate as complex or set and their role in the reaction.

**Figure 5.**
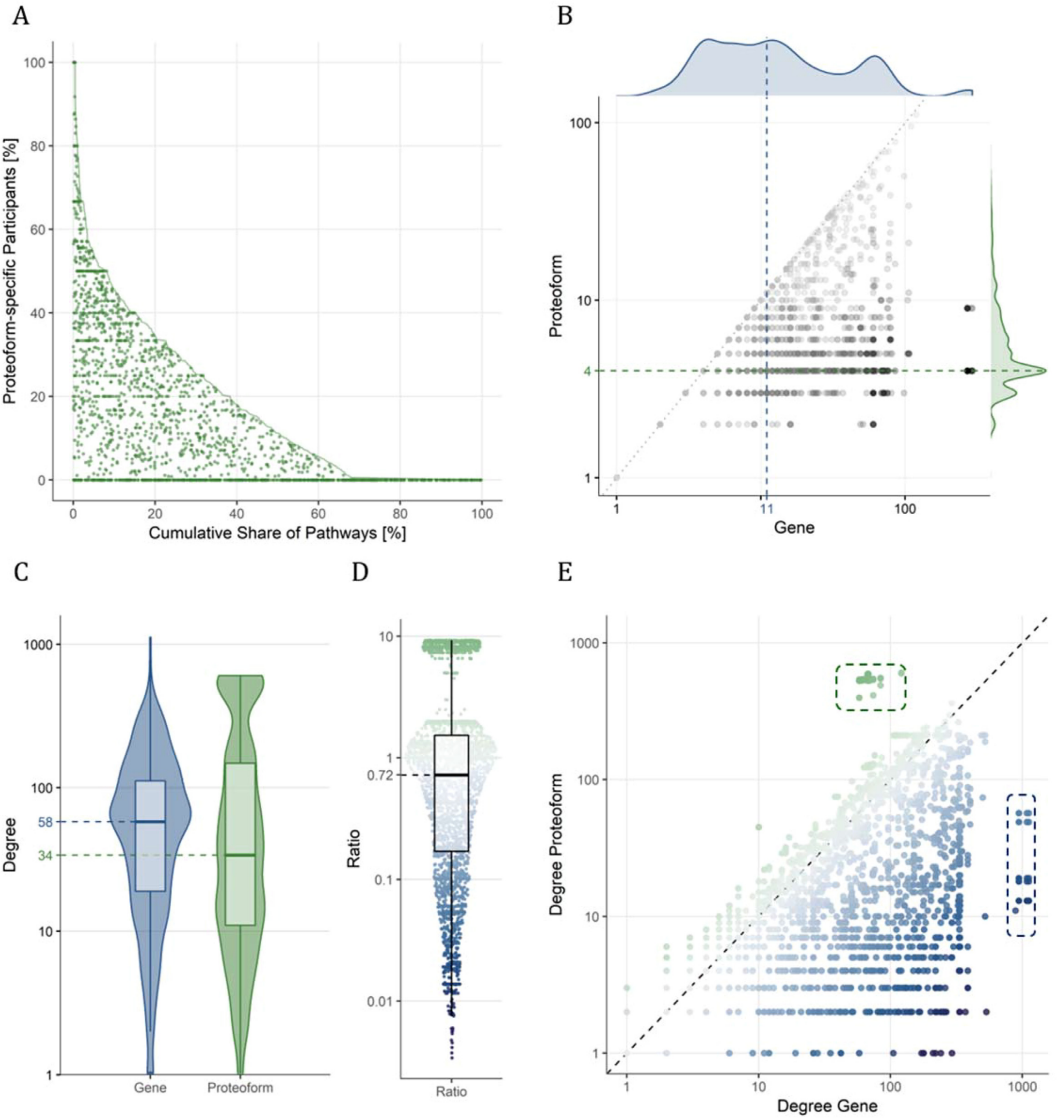
Prevalence of proteoforms in pathways. (A) The share of proteoform-specific participants in a pathway (*i.e*., proteins that are annotated with isoform and/or PTM information) is plotted against the cumulative share of pathways, going from the highest share of proteoforms to the lowest. The cumulative share of pathways is displayed with a solid green line. The share of proteoform-specific participants in each pathway is plotted with a green dot with a jitter on the *x*-axis between zero and the solid line. (B) For all proteoform-specific participants, the number of pathways mapped using the proteoform versus gene is plotted in black. The density of the number of pathways mapped is indicated at the top (blue) and right (green) for gene and proteoform matching, respectively. The median number of pathways mapped is indicated with dashed lines. (C) The violin and box plots of the degree, *i.e*., number of connections, for the proteoform-specific participants in a gene-centric or proteoform-centric network are plotted to the left (blue) and right (green), respectively. (D) The ratio of degrees, proteoform over gene, is plotted with a blue-gray-green gradient with the box plot overlaid in black. (E) The degree of the proteoform-specific participants in the proteoform-centric network is plotted against the degree in the gene-centric network. Dots are colored with a blue-gray-green gradient corresponding to the ratio in D. Outliers of high degree in the gene-centric but not in the proteoform-centric network are indicated with blue dashes to the right. Outliers of high degree in the proteoform-centric but not in the gene-centric network are indicated with green dashes to the top. Note that base 10 logarithmic scales are used for the axes in B, C, D, and E.

As displayed in Fig. 5A, 68% of the pathways present at least one proteoform-specific participant, *i.e*., with isoform or PTM annotation. The number of pathways containing a given gene product or proteoform is displayed in Fig. 5B, showing how using proteoforms allows distinguishing pathways more specifically than genes, with a median of 4 pathways matched per proteoform compared to 11 pathways per gene. When the input can be mapped to proteoforms, PathwayMatcher can restrict the search for reactions and pathways to those that specifically involve proteins in the desired form, hence reducing the number of possible connections for a given node in the resulting network. Conversely, the proteoform-centric network representation allows identifying interactions between multiple proteoforms originating from the same gene or protein, resulting in new connections compared to a gene-centric representation.

Figure 5C shows that the number of connections per proteoform is lower than the number of connections for the respective gene for most proteoforms, varying from a 300-fold decrease to a 10-fold increase. Interestingly, plotting the number of connections of a proteoform in gene-centric or proteoform-centric networks shows that the largest gene-centric hubs, corresponding to 5 genes, decompose into 127 proteoforms that do not outlie the distribution of the number of connections in the proteoform network (Fig. 5D). Conversely, a group of 484 densely connected outliers emerges from 44 genes.

In order to fully benefit from the gain in specificity of the proteoform-representation of pathways, it is necessary to exactly match the representation of proteoforms in Reactome. Any mismatch between the input data and the database would result in a loss of sensitivity. In practice, such mismatches can result from an incomplete proteoform representation in Reactome, where only the minimal set of modifications necessary to perform a reaction is annotated. Conversely, input data can present unresolved isoform, missing modifications, or inaccurate localization, especially in the case of bottom-up proteomics [11]. Since the size of the proteoform network is unknown to date, the effect of missing annotations in the database is not directly quantifiable.

To estimate the sensitivity of the matching, we mapped the phosphoproteome from Ochoa et al. [12] to Reactome using PathwayMatcher: among the 10,588 accessions representing phosphoproteins, 5,519 (52%) could be matched to an accession in Reactome, while among the 116,258 phosphosites reported, only 654 (<1%) could be matched exactly in Reactome. Accession matching is equivalent in terms of sensitivity and specificity to a gene-centric representation of pathways, while strict proteoform matching, requiring exact isoform and modification set, maximizes specificity at the cost of sensitivity.

In order to mitigate the sensitivity loss while maintaining specificity, we implemented multiple types of matching that present different levels of stringency, as detailed in the methods: (i) *One*, (ii) *One without PTM types*, (iii) *Superset*, (iv) *Superset without PTM types*, (v) *Subset*, (vi) *Subset without PTM types*, and (vii) *Strict*. Table 2 lists the share of phosphosites that can be matched to a proteoform in Reactome when querying the accession with a phosphorylation at the given site, and only at this site, with a tolerance of 5 amino acids. There, one can see that increasing the stringency of the matching dramatically reduces the sensitivity. Since both Reactome and the list of phosphosites represent a minimal set of modifications, the *Strict* matching is overly selective, while *Accession* and *Superset* include reactions where the proteins are not modified.

**Table 1.** Proteoforms of Figure 3B

#	Isoform	Modifications
1	Canonical	None
2	Canonical	pS15
3	Canonical	pS15 pS20 aceK120 aceK382
4	Canonical	pS15 pS20 aceK382
5	Canonical	pS15 pS20 aceK120
6	Canonical	pS15 pS20
7	Canonical	pS15 pS20 dimethR335 dimethR337 methR333
8	Canonical	pS15 pS20 ubiK
9	Canonical	pS15 pS33 pS46
10	Canonical	pS15 pS20 pS269 pT284
11	Canonical	pS15 pS20 methK370
12	Canonical	pS15 pS20 methK372
13	Canonical	pS15 pS20 methK382
14	Canonical	ubiK
15	Canonical	pS315
16	Canonical	pT55
17	Canonical	pS15 pS392
18	Canonical	pS37
19	Canonical	dimethK373
20	Canonical	sumoK386
21	Canonical	pS15 pS20 pS46
22	Canonical	pS15 pS20 pS392
23	Canonical	dimethK370 dimethK382

Only the canonical isoforms are annotated to date, as indicated in the second column. The posttranslational modification status is indicated in the third column with modification short name and modification site when annotated. Abbreviations: aceK, N6-acetyl-L-lysine; dimethK, N6, N6-dimethyl-L-lysine; dimethR, symmetric dimethyl-L-arginine; methK, N6-methyl-L-lysine; methR, omega-N-methyl-L-arginine; pS, O-phospho-L-serine; pT, O-phospho-L-threonine; ubiK, ubiquitinylated lysine; sumoK, sumoylated lysine.

**Table 2.** Share of the phosphosites from Ochoa et al. [12] matching to Reactome using different matching types

Matching Type	Share of Phosphosites Matched
*Accession*	57.44%
*Superset without PTM types*	56.38%
*Superset*	56.33%
*One without PTM types*	6.01%
*Subset without PTM types*	6.01%
*One*	1.27%
*Subset*	1.27%
*Strict*	0.15%

Proteoforms were constructed by adding a phosphorylation at the given site, and only at this site, and were queried against Reactome. The percentage of proteoforms matched is provided in the second column. A tolerance of 5 amino acids was used on the modification site. More details on this analysis can be found in the Methods section.

*Subset* and *One* represent the coverage of the input by Reactome. Here, *Subset* and *One* are equivalent because the input consists of single phosphosites. In a data set containing combinations of phosphosites, *Subset* would match proteoforms taking phosphosite combinations into account, while *One* would represent any proteoform with at least one matching phosphosite. The increased number of matches without PTM type can be imputed to mismatching PTM identifiers or the presence of other PTMs at the input sites or at neighboring positions.

**Figure 6.**
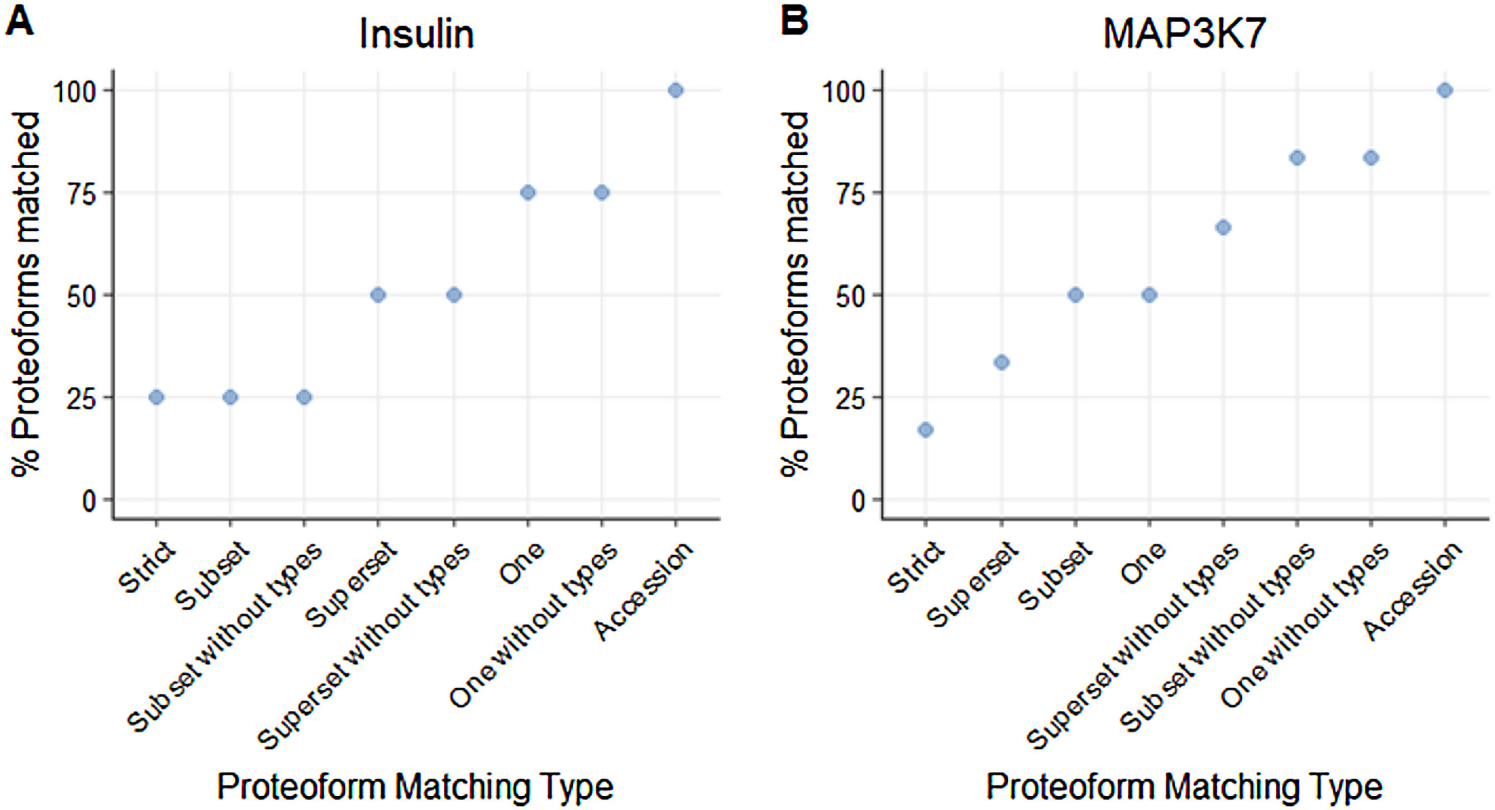
Two examples of proteoforms showing the proteoform matching results for each matching type. (A) Proteoform P01308; MOD:00087:53;MOD:00798:31;MOD:00798:43, from insulin (P01308), is matched against all modified proteoforms of insulin in Reactome. (B) Proteoform O43318;MOD:00047:184;MOD:00047:187, from “mitogen-activated protein kinase kinase kinase 7” (MAP3K7), is matched against all modified proteoforms of MAP3K7 in Reactome.

To illustrate the difference induced by each matching type on the proteoform matching, we calculated the percentage of proteoforms matched with selected example proteoforms. In Fig. 6, we present two example proteoforms, one from insulin (P01308) and one from mitogen-activated protein kinase kinase kinase 7 (MAP3K7). Insulin and MAP3K7 have five and seven different proteoforms annotated in Reactome, four and six of them with PTM annotation, respectively. By design, the *Strict* matching type matches only the original proteoform while the accession matching matches all proteoforms. The other matching types allow balancing between the two stringencies and display varying levels of specificity for those proteoforms. The results show that relaxing the stringency of the matching rapidly induces a loss in specificity due to the similarity of the different proteoforms of a given gene or protein.

Furthermore, we randomly selected proteoforms in Reactome and altered them by changing the type and localization of the PTMs to simulate mismatching or missing information, and the altered proteoforms were matched to Reactome; see details in the Methods section. In this setup, the share of altered proteoforms that can be recovered using the different matching types, referred to as *Original* matches, provides an estimate of the matching sensitivity in case of incomplete or mismatching proteoform definition. Conversely, the share of other proteoforms matching despite not being originally selected, referred to as *Other* matches, provides an estimate of the error rate, the complement of specificity.

**Figure 7.**
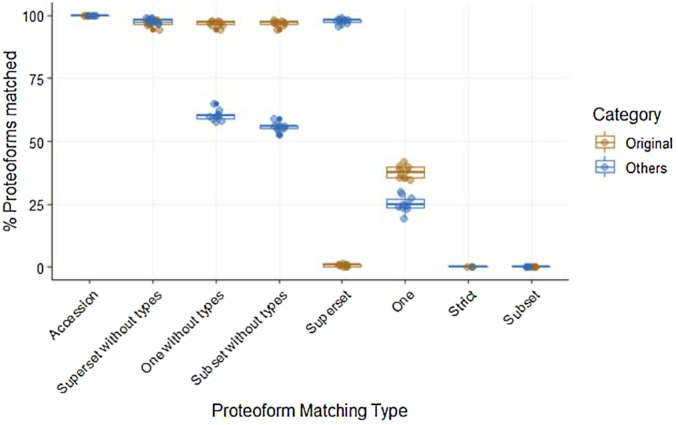
Percentage of proteoforms with at least one proteoform match in the database with each matching criterion. The total candidate proteoforms available are separated in two categories, *Original* and *Others. Original* is the proteoform in the database that was modified for the sampling, while *Others* are the proteoforms that share the same protein accession.

Fig. 7shows the percentage of proteoforms that matched at least one proteoform in the database separated on matching type. As expected, accession matching displays the highest sensitivity at the lowest specificity, while the *Strict* and *Subset* matching display the highest specificity at the lowest sensitivity. The *Superset* matching presented low sensitivity and low specificity, while the *One* matching presented a balance between specificity and sensitivity. Finally, the matching with no types presented similar trends but with almost maximum sensitivity and lower specificity. Together, these results show how relaxing the matching stringency allows balancing between sensitivity and specificity, and they demonstrate the importance of accurate proteoform definition in both the input and the reference knowledge base.

Through its paradigm shift, PathwayMatcher hence provides a fine-grained representation of pathways for the analysis of omics data. However, this comes at the cost of increased complexity: gene-centric networks comprise a limited number of nodes, approximately 20,000 for humans, whereas in a proteoform-centric paradigm, the human network is expected to have several million nodes [13]. With the current version of Reactome, building the gene- and proteoform-centric networks results in 9,759 and 12,775 nodes with 443,229 and 672,047 connections, respectively. We classified the nodes into two categories, canonical or specific gene products, depending on whether or not they represent the unmodified canonical isoform of a protein according to UniProt. Within the proteoform network, 432,169 connections between 9,694 nodes link two canonical gene products, 95,539 connections between 7,734 nodes involved one canonical and one specific gene product, and 2,806 nodes with 144,339 connections involved two specific gene products. More summary statistics on the underlying network can be found in the wiki of the PathwayMatcher repository.

**Figure 8.**
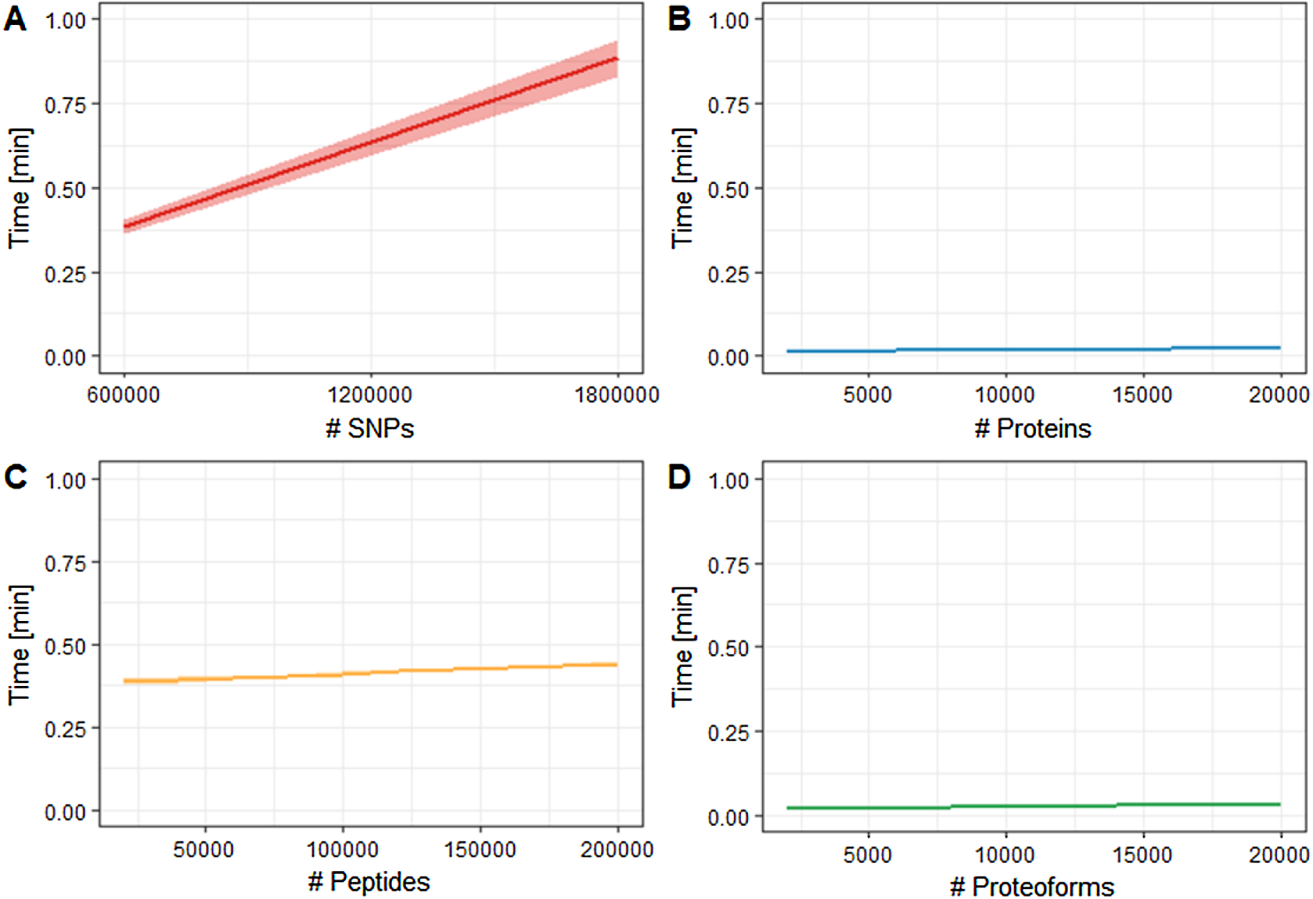
Performance of PathwayMatcher using (A) genetic variants as single-nucleotide polymorphisms (SNPs), (B) proteins, (C) peptides, and (D) proteoforms. Time in minutes is plotted against input size. The mean is displayed as a solid line and the 95% range as a ribbon (only visible in (A) due to the high reproducibility in other cases).

In addition to the increased size of the underlying network, matching proteoforms requires comparing isoforms and sets of modifications, possibly with tolerance and wildcards for the modification definition and localization, which is computationally much more intensive than simply comparing identifiers. Fig. 8 shows the performance of PathwayMatcher benchmarked against public data sets of (A) genetic variants, (B) proteins, (C) peptides, and (D) proteoforms.

For the proteins and proteoforms, the processing time increased linearly related to the query size with a small slope, making it possible to search all available proteins within a few seconds. As expected, protein identifiers provided the fastest response time, while proteoforms were the second fastest. Mapping peptides took approximately 30 seconds more, corresponding to the indexing time of the protein sequences database by PeptideMapper [7], after which the time increased linearly in a similar fashion as for proteins. For the genetic variants, an extra mapping step is required to map possibly affected proteins, adding additional computing time. The overall mapping time for a million single-nucleotide polymorphisms (SNPs) was less than a minute, which is acceptable compared to the other steps of a variant analysis pipeline. Note that the processing time was very reproducible across runs, where minor variation is only noticeable using genetic variants, resulting in very thin ribbons in Fig. 8B-D.

In conclusion, PathwayMatcher is a versatile application enabling the mapping of several types of omics data to pathways in reasonable time and can readily be included in bioinformatic workflows. It is important to underline that PathwayMatcher maps experimental data to pathways in a systematic and unbiased fashion, *i.e*., it collects all pathways containing at least one of the participant proteins or proteoforms of the input data and does not perform any filtering or biological inference. Through this process, it attempts at minimizing the prevalence of false negatives by considering all the possible pathways annotated in the reference database. It can, however, not control for missing annotation, *i.e*., what is not annotated in the knowledge base is not considered.

Furthermore, although PathwayMatcher implements an overrepresentation analysis module, we recommend that users rather interpret the results of the matching and the resulting networks using the systems biology method that best suits the experiment and biomedical context. Based on generic pathways, PathwayMatcher is not developed as a mechanism inference or validation tool, but as a hypothesis generation tool, helping to navigate large data sets and guide experiments to uncover biological processes relevant to specific research questions.

Thanks to the fine-grained information available in Reactome, PathwayMatcher supports refining the pathway representation to the level of proteoforms. To date, only a fraction of the several million expected proteoforms [13] has annotated interactions, but as the understanding of protein interactions continues to increase and the ability to identify and characterize them in samples progresses, proteoform-centric networks will surely become of prime importance in biomedical studies. Notably, the effect of genetic variation on genes, transcripts, and proteins is currently only partially resolved for a fraction of the genome. The rapid development of this field will make it possible to identify biological functions affected by variants within the human network. Refining its representation to the level of proteoforms will allow pinpointing more precisely reactions and pathways, and hence increase our ability to understand biological mechanisms and potentially identify druggable targets.

## Methods

### Implementation

PathwayMatcher is implemented in Java 8.0.

### Availability

PathwayMatcher is freely available at github.com/PathwayAnalysisPlatform/PathwayMatcher under the permissive Apache 2.0 license. It is also possible to use PathwayMatcher as a Docker image: hub.docker.com/r/lfhs/pathwaymatcher. PathwayMatcher can be obtained from the Bioconda channel of the Conda [14] package manager at bioconda.github.io/recipes/pathwaymatcher/README.html. Finally, PathwayMatcher is available as a Galaxy [15] tool in the Galaxy ToolShed [16] at toolshed.g2.bx.psu.edu/view/galaxyp/reactome_pathwaymatcher, where it can be readily integrated into analysis workflows. PathwayMatcher has also been installed into the public European Galaxy instance, usegalaxy.eu, making it possible to use the application without requiring any local configuration and just providing valid input files and options. The complete URL for the online tool is listed in reference [17].

Upon installation, PathwayMatcher can be used from the command line to query Reactome using various types of omics data. Either the “.jar” file is run directly using Java or the Docker image is instantiated to a container. Detailed information on implementation, installation, usage, and format specifications is available in the online documentation at github.com/PathwayAnalysisPlatform/PathwayMatcher/wiki.

### Input and output

Detailed and updated documentation of the input and output can be found in the online documentation at github.com/PathwayAnalysisPlatform/PathwayMatcher/wiki.

**Figure 9.**
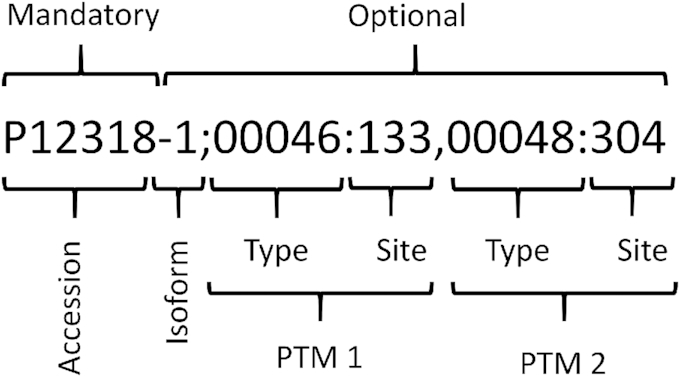
Example of proteoform notation, composed of a protein accession, an isoform number, and a set of PTMs.

As schematized in Fig. 9, a simple representation is used for proteoforms: (i) the UniProt protein accession and (ii) the set of PTMs separated by a semicolon “;”. The protein accession can include the isoform number specified with a dash “-”. The PTM set contains each PTM separated by a comma “,”. Each PTM is specified using a modification identifier and a site, separated by a colon “:”.

Note that the order of PTMs does not affect the search. The PTM identifier is a 5-digit identifier from the PSI-MOD Protein Modification [18]. The site is an integer specifying the 1-based index of the modified amino acid on the sequence as defined by UniProt. The modification site field is mandatory, and *?* or *null* indicates that the position is not known.

It is common to write the identifiers for the PTM types with the prefix “MOD:” before the 5 digits of the ontology term. PathwayMatcher also allows the user to write the identifier without the prefix. PathwayMatcher also allows querying all proteoforms modified at a given site using the “00000” wildcard for modification type combined with a matching type that does not consider the modification types such as *One* without types or *Subset* without types. For more details, see the Proteoform Matching subsection.

### Posttranslational modifications in the Reactome data model

**Figure 10.**
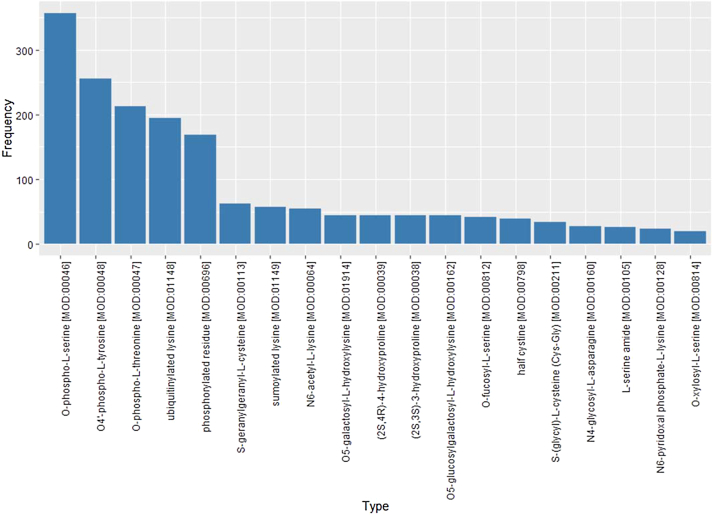
Prevalence of the different PTM annotations in Reactome. PTM labels are extracted from the Reactome database and the number of proteins annotated with the PTM is displayed for each label. If a protein is carrying multiple instances of the PTM, the PTM is counted only once.

The Reactome object model specifies physical entities (*e.g*., complexes, proteins, and small molecules) and proteins are annotated using unique identifiers. These entities participate in reactions in specific cellular compartments. They can also be connected to multiple instances of *Translational Modification* objects, which contain a specific coordinate on the protein sequence and an identifier following the PSI-MOD ontology [18]. The portion of physical entities referring to proteins is associated with another class of objects as reference entities, which contain protein annotations in external databases such as UniProt [19]. Therefore, a proteoform is represented as a physical entity associated with a set of modifications for specific processes at a specific subcellular location. Each modification has a PSI-MOD ontology identifier as type and an integer coordinate for the site in the peptide sequence where the modification occurs. The coordinate can be *?* or *null* when the site is not known. Reactome annotates 127 different protein modifications for humans, of which Fig.10 Reference source not found. displays the most frequent.

### Proteoform matching

Searching pathways using gene names or protein accessions solely requires mapping a string of characters between the input and the knowledge base. In order to map the proteoforms to reactions and pathways, it is necessary to decide if the proteoforms in the input are equivalent to the proteoforms annotated in the reference database, Reactome, taking into account the protein accession, isoform information, and the set of PTMs. Two proteoforms can have all, some, or none of these elements in common. We defined a set of criteria to match two proteoforms, one from the input and another from the reference database. First, identical protein accession and isoform numbers are required for a match: either both proteoforms are from the canonical isoform (*e.g*., P31749) or from the same isoform (*e.g*., P31749–3). Then, the PTMs carried by each proteoform are compared using the modification type and the modification site on the protein sequence. For 2 PTMs to match, their modification type as defined by the PSI-MOD ontology [18] needs to be identical and the distance between their sites must be below a user-provided margin, as detailed in Table 3.

**Table 3.** Posttranslational modification coordinates criteria for comparison

Input	Reference	Margin	Matched	Comment
17	17	0	Yes	Equal
16	17	0	No	Out of margin
7	13	5	No	Out of margin
8	13	5	Yes	In margin
19	13	5	No	Out of margin
0	2	5	No	Input in margin, but 0 is not a valid coordinate
-1	2	5	No	Input in margin but negative
?, empty, null	c	k	Yes	Input is less specific
c	?, empty, null, -1	k	Yes	Input is more specific
?, empty, null	?, empty, null, -1	k	Yes	Equally unspecific
Negative int, zero	Any	k	No	Negative or zero input are invalid

This table compares the value of a PTM coordinate of an input Proteoform with the value of a PTM coordinate in a reference proteoform. The letter k represents any positive integer.

PTM

Different matching types are implemented in PathwayMatcher for the PTM sets:

- *Strict*: the input and reference proteoforms have the same number of PTMs and every PTM of the input proteoform matches a PTM in the reference proteoform.
- *Superset*: every PTM of the reference proteoform matches a PTM of the input proteoform, but some PTMs in the input proteoform may not match PTMs in the reference proteoform.
- *Subset*: every PTM of the input proteoform matches a PTM of the reference proteoform, but some PTMs of the reference proteoform may not match PTMs in the input proteoform.
- *One*: at least one PTM of the input proteoform matches a PTM of the reference proteoform.

In addition, *Superset without PTM types, Subset without PTM types*, and *One without PTM types* are identical to *Superset, Subset*, and *One*, respectively, but do not account for modification type in PTM matching. Finally, note that for the *Strict* matching, the PTMs match when their sites are exactly identical and no margin is allowed: either both are the same positive integer or both are *null* or *?*.

For details and examples to run PathwayMatcher with the different matching criteria, see the online documentation (github.com/PathwayAnalysisPlatform/PathwayMatcher/wiki/Proteoform-matching).

Additional considerations:

- Negative, zero, or floating-point values are invalid as sequence coordinates in the input.
- The margin to compare the coordinates must be a positive integer.

### Sensitivity analysis

In order to estimate the prevalence of missing annotation in Reactome, we evaluated the matching power of each matching type of PathwayMatcher using a reference list of 116,258 phosphosites obtained from Ochoa et al. [12]. Each phosphosite was transformed into a proteoform, which had the same protein accession and a single PTM at the given site. The PTM accession number 00046, 00047, or 00048 was used if the phosphorylated amino acid reported was a serine, a threonine, or a tyrosine, respectively. Each of the proteoforms with a single phosphorylation was matched against all proteoforms available in Reactome using PathwayMatcher. The share of phosphosites yielding a match for each matching type is available in Table 2.

Subsequently, we evaluated the robustness of each matching type by selecting sets of proteoforms from Reactome, altering them, and matching them back.

First, we selected the proteins that had multiple proteoforms with at least one PTM (1,364 proteins). Then, we gathered all those posttranslationally modified proteoforms and altered them: (1) for the proteoforms with one or more PTMs, the type of the first PTM was replaced by “00000” and modification sites were increased by 5 positions; (2) for the proteoforms with two or more PTMs, the site of the second PTM was moved as well.

Then, we took ten samples of 300 altered proteoforms and matched them to proteoforms in Reactome using PathwayMatcher. For each matching type, we calculated the percentage proteoforms in the sample that matched any proteoform in the database$.$

The results for all ten samples are shown in Fig.   7, where we split the matching of the original sample proteoforms and other candidate proteoforms.

### Mapping omics data to pathways

**Figure 11.**
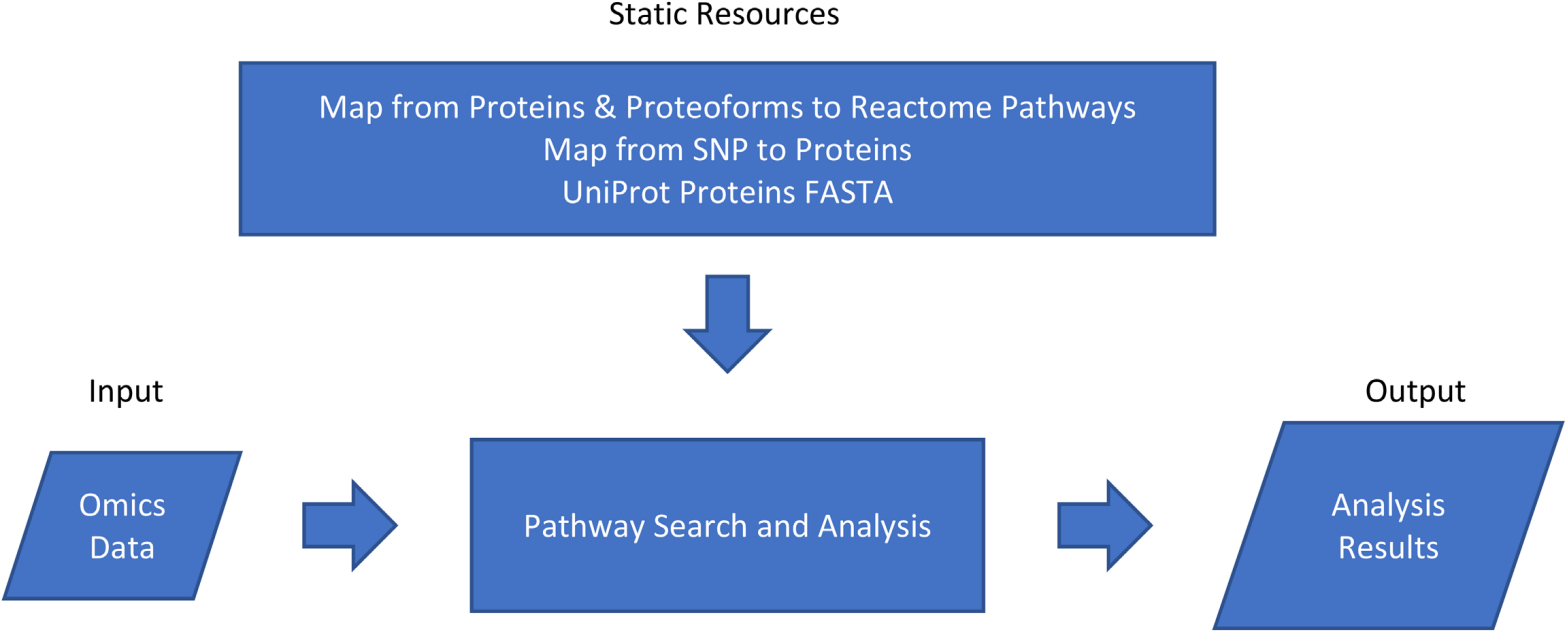
PathwayMatcher general overview. The program takes the user input in the form of omics data files and the reference pathways from the database as input. It then executes the search and analysis algorithm to create a resulting list of output files.

The input is mapped to proteins or proteoforms to find the reactions where the input entities are participants (Fig.11). The input is mapped to proteins when data types without PTMs or specific translation products are specified; otherwise, a mapping to proteoforms is used. When one type of data yields multiple results due to ambiguity (*e.g*., a SNP or peptide mapping multiple proteins), all the possibilities are included in the search entities.

When a list of SNPs is provided, mapping from the Ensembl Variant Effect Predictor [5] is used to find the possibly affected proteins. When peptides are provided, their sequence is mapped to UniProt protein identifiers [6] using PeptideMapper [7] and possible proteoforms are constructed. When proteins or proteoforms are available, PathwayMatcher maps them to reactions and pathways using data structures embedded in the PathwayMatcher jar file. These data structures are extracted from the Reactome Neo4j graph database [19] and serialized. All mapping files are available in a dedicated repository: github.com/PathwayAnalysisPlatform/MappingFiles.

In addition, we made it possible for the user to generate new mapping files as detailed in the PathwayMatcher repository (github.com/PathwayAnalysisPlatform/PathwayMatcher/tree/master/src/main/java/extractor). PathwayMatcher can then be executed with the new set of mapping files as provided by the user.

### Overrepresentation analysis

The matching of each entity to a given pathway is modeled as a Bernoulli trial with two possible outcomes: success or failure, depending on whether the protein or proteoform is a participant of a reaction in the pathway. Trials are considered independent from each other, meaning that the outcome of previous trials does not affect the next. Finally, the probability of success is calculated by the proportion of choosing a protein in a pathway over the total number of possible proteins, and therefore the probability is constant over all trials.

First, we search all the input entities (proteins or proteoforms) across all the pathways and count how many of them were found in each pathway. The number of entities found in a pathway is taken as the number of successful trials. Then, with the binomial probability distribution, we calculate how likely it would be to get a result equal to or more extreme than the current result (the same number or more proteins or proteoforms in the pathway), given that the input (proteins or proteoforms) was randomly selected [9].

This is done using the cumulative distribution function for the binomial distribution, which calculates the probability of getting at most *k* successes out of *n* trials, with a probability *p* ∈ [0,1], where *X* is a random variable following the binomial distribution, as detailed in Equation 1.

(1) \begin{equation*} F\ \left( {k,n,p} \right) = \Pr \left( {X \le k} \right)\ = \ \mathop \sum \limits_{i\ = \ 0}^{\rm{k}} \left( {\begin{array}{@{}*{1}{c}@{}} n\\ i \end{array}} \right){p^i}{\left( {1 - p} \right)^{n - i}} \end{equation*}

For each pathway, *p* is set to the ratio between the number of total proteins or proteoforms in the pathway and the total possible entities in the database, *n* is the number of proteins or proteoforms in the input sample, *k* is the number of proteins successfully mapped in the pathway, and*X* is the number of entities found in the current pathway after the search.

Finally, given that the *P*-value requires the calculation of the probability of an equal or more extreme result, we use the complement of Equation 1 to calculate the probability of getting at least *k* successful trials out of *n*, as stated in Equation 2.

(2) \begin{equation*} {\rm{Pr}}\left( {{\rm{X\ }} \ge {\rm{\ k}}} \right){\rm{\ }} = {\rm{\ }}1{\rm{\ }} - {\rm{\ Pr}}\left( {{\rm{X}} \le {\rm{k}} - 1} \right) \end{equation*}

The calculations for proteins or proteoforms are similar but are performed separately depending on the input. If the input consists of protein accessions, the number of participants is calculated by only considering proteins. On the other hand, for the proteoform input, the number of entities in the pathways and the database are the participant proteoforms.

### Performance benchmark

The performance of PathwayMatcher was evaluated using data sets of different sizes obtained from sampling publicly available resources:

- Proteins: human complement of the UniProtKB/Swiss-Prot database (release 2017_10)
- Peptides: ProteomeTools [20] as available in PRIDE [21], data set PXD004732, release date January 23, 2017
- Genetic variants: variants from the human assembly GRCh37.p13
- Proteoforms: annotated proteoforms in Reactome Graph database version 62

Performance testing was done using a standard desktop computer (Intel® Core™ i7–6600U CPU @ 2.60 GHz with 2 cores using 64-bit Windows 10 with Java SE 1.8.0_144 on SSD). Details and code are available at github.com/PathwayAnalysisPlatform/PathwayMatcher/wiki/Performance.

### Metrics and figures

The metrics presented in this article were obtained by querying the Reactome graph database directly [22]. The queries used can be found in the online documentation at github.com/PathwayAnalysisPlatform/PathwayMatcher/wiki/queries.

The figures in this article were built in R version 3.4.1 (2017–06-30)—“Single Candle” (r-project.org) using the following packages: ggplot2, ggrepel, igraph, scico, grid, purr, dplyr, graphlayouts, and gtable. The R scripts used to build the figures are available in the tool repository at github.com/PathwayAnalysisPlatform/PathwayMatcher_Publication/tree/master/R.

### Availability of supporting source code and requirements

**Project name:** PathwayMatcher

**Project home page:** github.com/PathwayAnalysisPlatform/PathwayMatcher

**Operating system(s):** Platform independent

**Programming language:** Java

**Other requirements:**

**License:** Apache 2.0

RRID: SCR_01 6759

## Availability of Supporting Data

Snapshots of our code and other supporting data are available in the *GigaScience* repository, GigaDB [23].

## Declarations

### List of abbreviations

PTM: posttranslational modification.

### Ethics approval and consent to participate

Not applicable.

### Consent for publication

Not applicable.

### Competing interests

The authors declare that they have no competing interests.

## Funding

LFHS, SJ, PRN, and MV are supported by the European Research Council and the Research Council of Norway. BB, CH, and HB are supported by the Bergen Research Foundation. HB is also supported by the Research Council of Norway. LFHS, SJ, PRN, and MV are supported by the European Research Council and by the Research Council of Norway. This work has been supported by National Institutes of Health BD2K grant (U54 GM114833) and National Human Genome Research Institute at the National Institutes of Health Reactome grant (U41 HG003751).

## Authors' contributions

LFHS did most of the programming, testing, and documentation and wrote the manuscript. BB and CH contributed with programming, testing, documentation, ideas, and manuscript writing. AF, SJ, and PRN contributed with ideas and manuscript writing. HB contributed with ideas, supervised the work, and wrote the manuscript. HH contributed with project design, manuscript writing, and supervised the work. MV contributed with project design, programming, documentation, and testing; supervised the work; and wrote the manuscript. All authors participated in the preparation of the manuscript.

## Supplementary Material

giz088_GIGA-D-18-00514_Original_Submission

giz088_GIGA-D-18-00514_Revision_1

giz088_Response_to_Reviewer_Comments_Original_Submission

giz088_Reviewer_1_Report_Original_Submission David Lahnemann -- 1/10/2019 Reviewed

giz088_Reviewer_1_Report_Revision_1 David Lahnemann -- 6/14/2019 Reviewed

giz088_Reviewer_2_Report_Original_Submission Marcella Nunes Melo-Braga, PhD -- 2/21/2019 Reviewed

giz088_Reviewer_2_Report_Revision_1 Marcella Nunes Melo-Braga, PhD -- 6/11/2019 Reviewed

## References

[1] Smith LM, Kelleher NL; The Consortium for Top Down P. Proteoform: a single term describing protein complexity. Nat Methods. 2013;10(3):186–7. 23443629 10.1038/nmeth.2369 PMC4114032

[2] Seet BT, Dikic I, Zhou M-M, Pawson T Reading protein modifications with interaction domains. Nat Rev Mol Cell Biol. 2006;7:473. 16829979 10.1038/nrm1960

[3] Menche J, Sharma A, Kitsak M, et al. Uncovering disease-disease relationships through the incomplete interactome. Science. 2015;347:6224. 10.1126/science.1257601 PMC4435741 25700523

[4] Fabregat A, Jupe S, Matthews L, et al. The Reactome Pathway Knowledgebase. Nucleic Acids Res. 2018;46(D1):D649–D55. 29145629 10.1093/nar/gkx1132 PMC5753187

[5] McLaren W, Gil L, Hunt SE, et al. The Ensembl Variant Effect Predictor. Genome Biol. 2016;17(1):122. 27268795 10.1186/s13059-016-0974-4 PMC4893825

[6] The UniProt C. UniProt: the universal protein knowledgebase. Nucleic Acids Res. 2017;45(D1):D158–D69. 27899622 10.1093/nar/gkw1099 PMC5210571

[7] Kopczynski D, Barsnes H, Njolstad PR et al. PeptideMapper: efficient and versatile amino acid sequence and tag mapping. Bioinformatics. 2017;33(13):2042–4. 28334306 10.1093/bioinformatics/btx122

[8] Nesvizhskii AI, Aebersold R Interpretation of shotgun proteomic data: the protein inference problem. Mol Cell Proteomics. 2005;4(10):1419–40. 16009968 10.1074/mcp.R500012-MCP200

[9] García-Campos MA, Espinal-Enríquez J, Hernández-Lemus E Pathway analysis: state of the art. Front Physiol. 2015;6:383. 10.3389/fphys.2015.00383 PMC4681784 26733877

[10] Benjamini Y, Hochberg Y Controlling the false discovery rate: a practical and powerful approach to multiple testing. J R Stat Soc Series B Methodol. 1995:57:289–300.

[11] Schaffer LV, Millikin RJ, Miller RM, et al. Identification and quantification of proteoforms by mass Ssectrometry. Proteomics. 2019;19(10):1800361. 10.1002/pmic.201800361 PMC6602557 31050378

[12] Ochoa D, Jarnuczak AF, Gehre M, et al. The functional landscape of the human phosphoproteome. bioRxiv. 2019; doi:10.1101/541656. PMC7100915 31819260

[13] Aebersold R, Agar JN, Amster IJ, et al. How many human proteoforms are there?. Nat Chem Biol. 2018;14:206. 29443976 10.1038/nchembio.2576 PMC5837046

[14] Grüning B, Dale R, Sjödin A, et al. Bioconda: sustainable and comprehensive software distribution for the life sciences. Nat Methods. 2018;15(7):475–6. 29967506 10.1038/s41592-018-0046-7 PMC11070151

[15] Afgan E, Baker D, Batut B, et al. The Galaxy platform for accessible, reproducible and collaborative biomedical analyses: 2018 update. Nucleic Acids Res. 2018;46(W1):W537–W44. 29790989 10.1093/nar/gky379 PMC6030816

[16] Blankenberg D, Von Kuster G, Bouvier E, et al. Dissemination of scientific software with Galaxy ToolShed. Genome Biol. 2014;15(2):403. 25001293 10.1186/gb4161 PMC4038738

[17] https://usegalaxy.eu/?tool_id=toolshed.g2.bx.psu.edu%2Frepos%2Fgalaxyp%2Freactome_pathwaymatcher%2Freactome_pathwaymatcher, Accessed July 24, 2019

[18] Montecchi-Palazzi L, Beavis R, Binz PA, et al. The PSI-MOD community standard for representation of protein modification data. Nat Biotechnol. 2008;26(8):864–6. 18688235 10.1038/nbt0808-864

[19] Natale DA, Arighi CN, Blake JA, et al. Protein Ontology (PRO): enhancing and scaling up the representation of protein entities. Nucleic Acids Res. 2017;45(D1):D339–D46. 27899649 10.1093/nar/gkw1075 PMC5210558

[20] Zolg DP, Wilhelm M, Schnatbaum K, et al. Building ProteomeTools based on a complete synthetic human proteome. Nat Methods. 2017;14:259. 28135259 10.1038/nmeth.4153 PMC5868332

[21] Vizcaíno JA, Csordas A, del-Toro N, et al. 2016 update of the PRIDE database and its related tools. Nucleic Acids Res. 2016;44(D1):D447–D56. 26527722 10.1093/nar/gkv1145 PMC4702828

[22] Fabregat A, Korninger F, Viteri G, et al. Reactome graph database: efficient access to complex pathway data. PLoS Comput Biol. 2018;14(1):e1005968. 29377902 10.1371/journal.pcbi.1005968 PMC5805351

[23] Hernández Sánchez LF, Burger B, Horro C, et al. Supporting data for “PathwayMatcher: proteoform-centric network construction enables fine-granularity multi-omics pathway mapping” GigaScience Database. 2019 10.5524/100621. PMC6667378 31363752

