# Supplementary material for: PathwayMatcher: proteoform-centric network construction enables fine-granularity multiomics pathway mapping
Source: Gigascience. 2019 Jul 30;8(8):giz088. doi: 10.1093/gigascience/giz088 (PMC6667378; doi:10.1093/gigascience/giz088)
Supplement: giz088_GIGA-D-18-00514_Revision_1 [file giz088_giga-d-18-00514_revision_1.pdf]

GigaScience

PathwayMatcher: proteoform-centric network construction enables fine-granularity multi-omics pathway mapping

--Manuscript Draft--

**Manuscript Number:**	GIGA-D-18-00514R1
**Full Title:**	PathwayMatcher: proteoform-centric network construction enables fine-granularity multi-omics pathway mapping
**Article Type:**	Technical Note
**Funding Information:**	
**Abstract:**	Background<br>Mapping biomedical data to functional knowledge is an essential task in bioinformatics and can be achieved by querying identifiers, e.g. gene sets, in pathway knowledgebases. However, the isoform and post-translational modification states of proteins are lost when converting input and pathways into gene-centric lists.<br><br>Findings<br>Based on the Reactome knowledgebase, we built a network of protein-protein interactions accounting for the documented isoform and modification statuses of proteins. We then implemented a command line application called PathwayMatcher (github.com/PathwayAnalysisPlatform/PathwayMatcher) to query this network. PathwayMatcher supports multiple types of omics data as input, and outputs the possibly affected biochemical reactions, subnetworks, and pathways.<br><br>Conclusions<br>PathwayMatcher enables refining the network-representation of pathways by including proteoforms defined as protein isoforms with post-translational modifications. The specificity of pathway analyses is hence adapted to different levels of granularity and it becomes possible to distinguish interactions between different forms of the same protein.
**Corresponding Author:**	Marc Vaudel<br><br>NORWAY
**Corresponding Author Secondary Information:**	
**Corresponding Author's Institution:**	
**Corresponding Author's Secondary Institution:**	
**First Author:**	Luis Francisco Hernández Sánchez
**First Author Secondary Information:**	
**Order of Authors:**	Luis Francisco Hernández Sánchez
	Bram Burger
	Carlos Horro
	Antonio Fabregat
	Stefan Johansson
	Pål Rasmus Njølstad
	Harald Barsnes
	Henning Hermjakob
	Marc Vaudel
**Order of Authors Secondary Information:**	

Powered by Editorial Manager® and ProduXion Manager® from Aries Systems Corporation

Response to Reviewers:

Reviewer reports:
Reviewer #1:

general review

The manuscript presents a software that extends beyond existing query methods for biological pathway databases in that it allows querying for specific proteoforms of a protein instead of only the consensus protein entry. It establishes different matching setups for proteoforms with varying strictness, describes the developed software and provides some basic characterization of how proteoform identifier queries can have an increased specificity compared to protein or gene identifier queries.

With the description of a new software tool and the augmented data base it uses, this manuscript is a good fit for publication in Giga Science.
The software and the respective data are available with an Apache license and are mostly well-documented in the manuscript and in a repository wiki. Code and data for the figures generated for the manuscript are available in the same repository.

With the two major questions below addressed, I see the minimum standards of reporting fulfilled and have no objections to publication.

Answer: We have carefully examined all comments and corrected our work accordingly. We are convinced that the software, documentation, and manuscript were greatly improved thanks to the reviewer's comments. We would therefore like to express our gratitude for this outstanding review.

requested revisions for publication

The following two main questions should in my opinion be addressed before publication. Below come further smaller comments, spotted errors and recommendations regarding the software, the data and the manuscript text itself.

extended description of Extractor

The abstract states:
Based on the Reactome knowledgebase, we built a network of protein-protein interactions accounting for the documented isoform and modification statuses of proteins.

To me, this indicates that this generated network is a major part of the innovation presented in this manuscript. The data availability and method description requirements of Giga Science would in my opinion therefore require a description of what the respective Extractor tool does both in the manuscript here and in the README of the repository for its code (<https://github.com/PathwayAnalysisPlatform/Extractor>).

I would especially welcome a description of which exact resources are used to construct this network, and how it is constructed--i.e. what is matched to what. From the Extractor repository, it looks to me, as though data is extracted from the Ensembl Variant Effect Predictor (vep), ProteomeTools (peptides), PSIMOD and Reactome (neo4j). Are these all used to create a single network? Which versions of each data base were used in the current version of PathwayMatcher?

In connection to this Extractor point, please also see the recommendation for separation of data and code in the `data` section below.

Answer: We thank the reviewer for this suggestion. We agree that the manuscript was lacking details on the Extractor, and as the reviewer points out here and in the data section, our architecture was not efficient. We have therefore refactored our repositories entirely so that the organization is cleaner and the system easier to

Powered by Editorial Manager® and ProduXion Manager® from Aries Systems Corporation

maintain. Notably, the code of the different modules, including Extractor, is now integrated into the PathwayMatcher repository. The structure of the application is now described in the wiki, with specific readme files for the different modules:

github.com/PathwayAnalysisPlatform/PathwayMatcher/tree/master/src/main/java/extractor/
github.com/PathwayAnalysisPlatform/PathwayMatcher/tree/master/src/main/java/model/
github.com/PathwayAnalysisPlatform/PathwayMatcher/tree/master/src/main/java/methods/
github.com/PathwayAnalysisPlatform/PathwayMatcher

The reviewer is correct that we use third-party tools and resources for the creation of the network and to allow the matching of different types of omics data. For the sake of ease of installation, portability, and performance, these third-party tools are not used when running PathwayMatcher, but static mappings are created by the extractor module at every release. We have extended the manuscript and documentation to clarify and better detail our usage of third-party tools and resources.

decreased sensitivity?

While the manuscript clearly makes the point that using proteoform queries will improve specificity of the results, by narrowing down on fewer pathways and interactions than protein / gene queries would, it lacks a test and discussion of sensitivity. My main question would be:

Will using the proteoform query result in missing some potential pathways for lack of proper proteoform annotation to date?
This boils down to: Will available proteoforms of a gene always recreate all the interactions reported for that gene? Or asked the other way around: Are there genes where (a lot of or certain) interactions are only annotated for the main gene identifier, but not annotated for any of its reported proteoforms, while there are proteoforms reported?
I think that this could mostly be addressed by characterizing the current proteoform annotation status of the underlying Reactome data base, e.g. answering questions like: Do genes with few annotated proteoforms have lots of gene-centric annotations that are not annotated to a specific proteoform? Does this number decrease with more proteoforms annotated? Here, both summary statistics and individual show-cases would be helpful, along the lines of what the manuscript nicely does for specificity.

Answer: The reviewer is correct that the sensitivity of the search is decreased when proteoform annotation is mismatching or missing. The annotation can be incomplete or inaccurate in the reference database, but also in the data, for example with bottom-up proteomics data. In some cases, one can speculate that the loss of sensitivity might even shadow the gain in specificity.

The reviewer is correct in that the gene-centric representation encompass all proteoform-centric edges, without the distinction of proteoform-proteoform interaction between proteoforms from the same gene. In contrast, the proteoform-centric representation contains the gene-centric network, but with more details. As a consequence, it is possible to build the gene-centric network from the proteoform-centric representation, but not the other way around.
To give the user more flexibility, we implemented many ways of tuning the matching: by relaxing proteoform matching tolerances, the user can increase sensitivity at the cost of specificity, up to the extreme case of matching by accession, where there is no loss of sensitivity but no gain in specificity. We anticipate that users will use different stringencies in proteoform matching based on the type of data queried, ranging from exact proteoform matching to gene matching, hence balancing specificity and sensitivity. It will even be possible to do differential analyses using different levels of stringencies in matching.

Powered by Editorial Manager® and ProduXion Manager® from Aries Systems Corporation

To highlight this, we conducted a sensitivity and specificity analysis and included all results in the manuscript. As suggested by the reviewer, we used individual show-cases (namely Insulin and MAP3K7) as well as summary statistics. We also use a recently published meta-analysis of phosphoproteomics data representing over 100,000 phosphosites. We are convinced that the results of these analyses greatly improved the text and will be valuable to the users when tuning PathwayMatcher. We would therefore like to thank the reviewer for this challenging but very useful comment.

software

installation

It is very much appreciated, that various options for installation and usage are offered, that all aim at a simple installation and reproducible usage. I have explicitly tried out the installation via bioconda and can confirm that it installs seamlessly.

Answer: We thank the reviewer for underlying our efforts in integrating our software in multiple bioinformatic environments. This has been greatly enabled by the Galaxy community who deserves acknowledgement for their indefectible support.

documentation

Both the installation process and the usage are well documented, with the documentation Wiki linked to directly in the main README of the software repository. Example data for all possible input data is provided. As proteoform input is a unique feature of PathwayMatcher, I used this as a general test case for trying out the software.

The software worked well and produced the described outputs. One thing I was missing in documentation were suggestions on how to visualise and / or analyse the graph files that are an optional output. Here, I could imagine both a general pointer to software and / or a pointer to scripts used in the manuscript or elsewhere.

Answer: We thank the reviewer for this suggestion. Links to follow-up analysis tools (Cytoscape, IGraph), and to the scripts used to generate the examples featured in the paper have been added to the documentation:
github.com/PathwayAnalysisPlatform/PathwayMatcher/wiki/Protein-connection-graph#visualization-and-follow-up-analysis
github.com/PathwayAnalysisPlatform/PathwayMatcher_Publication/tree/master/R
github.com/PathwayAnalysisPlatform/PathwayMatcher/wiki/queries

command-line interface

The command-line interface provides a useful help message and provides standard flags like `--version`. Some minor things I have stumbled upon where I would suggest future improvements--but which I would not make a requirement for publication--are:
* It seems like not all command line options are displayed in the `--help` output, e.g. I found the hidden `--version` tag.
The options for help and version are now visible.
* It would be useful to have the help message display the defaults for command line arguments. I came across this for the match type, when using the proteoform.

* It would be useful to have a quick description of the output files generated in the help message, so not to have to refer to the wiki for that.
* It would be useful to be able to specify the names of individual output files for easier pipeline integration of PathwayMatcher, where usually input and output files have to be named explicitly. The `--output` path option makes this possible, but individual options for

Powered by Editorial Manager® and ProduXion Manager® from Aries Systems Corporation

the file names with the current values as defaults would in my opinion increase usability.
* Instead of one command for all possible input types, I would recommend using different subcommands instead of a command line argument for input type. This would allow for different interfaces for different formats, as e.g. for proteoform input you have to specify the matching type, whereas other input types don't need this. So a usage could look like something along the lines of `pathwaymatcher match-proteoforms <options>` or `java -jar PathwayMatcher.jar match-proteforms <options>`.

From the above points, it seems like the currently used CLI library is probably not the best choice. As I am not a Java programmer, I am only guessing here and cannot recommend a better command line interface library, but maybe this stackexchange thread is useful: <https://softwarerecs.stackexchange.com/questions/16450/what-library-should-i-use-for-handling-cli-arguments-for-my-java-program>

Answer:
The options for help and version are now visible and can be executed with the short (“-v”) and long (“--version”) arguments. The default values for range and matchType are shown in the help text. The other arguments have no default value, but the user is now required to provide the values in order to execute. We added a brief description of the output files in the help text and what each command does.

We replaced the command line interface library from Apache CLI to Picocli. The “inputType” parameter was removed in favor of the subcommands interface provided by the new library. We also made it possible for the user to name the output files produced by a command execution using a common prefix, which allows using the same output folder for different runs without overwriting of the results.

We thank the reviewer for these suggestions which greatly simplify the usage of the tool.

code

Upon a quick glance by a non-Java coder, the code looks well organised and seems to contain extensive tests for the different possible input formats, which is very much appreciated. The modules in the separate repositories (Model, Method and Extractor) all still lack a useful README file, which would help grasping how they work together, but the code itself contains useful comments.

Answer: We thank the reviewer for his appreciation of our effort to abide by programming good practices, and for taking the time to dive in our code. As suggested, a README.md file has been added to the Extractor, Model and Methods modules. As detailed in our answer to the first comment, the code architecture has been refactored and better documented.

data

Example input data is available for all possible input types and output formats are well described in the documentation. The data base needed for mapping inputs to Reactome pathways is provided with the executable and is thus directly available.

The last point, while facilitating accessibility, is also a point of criticism for me. With the data base included in the main software repository, including multiple versions of it in the `.git` history, the repository currently has a size of 2 GB and will drastically increase in size with every new version of the data base generated--which will become necessary with every new version of the Reactome data base that someone wants to use with PathwayMatcher. Also, there will be differences between the version numbers of the software and the Reactome

Powered by Editorial Manager® and ProduXion Manager® from Aries Systems Corporation

data base mapping packaged with it and with the current setup it will not be clear to users which is which--from what I gather, I cannot currently query the command-line tool for the Reactome data base used. I would therefore recommend separating out the network generated with Extractor from the software repository, and distributing it separately (e.g. via GigaDB: <http://gigadb.org/>, Open Science Framework: <https://osf.io/> or something similar, e.g. check via: <https://www.re3data.org>). This will reduce the repo size drastically, from currently above 2 GB to probably a couple of MB, and will then allow for a separate versioning of the software and versions of the network generated from different versions of Reactome. To remove large files from git history, e.g. consider the respective GitHub tutorial: <https://help.github.com/articles/removing-sensitive-data-from-a-repository/>

A further reduction in repo size could be achieved by also separating out the manuscript (including code for plots) from the software code into a separate repository. As the manuscript and associated code will not change further after publication, such a repository would not change further, whereas the software will live on.

Answer: Once again we thank the reviewer for very relevant suggestions on how to organize our codebase. We have now refactored our repositories and better described the structure in the documentation: all code necessary to build and use the network are now in the same repository (github.com/PathwayAnalysisPlatform/PathwayMatcher), and all large files are now in a separate repository (github.com/PathwayAnalysisPlatform/MappingFiles), as well as all code and resources used for the paper (github.com/PathwayAnalysisPlatform/PathwayMatcher_Publication). As a result, the main repository is much smaller, and the cloning of the repository takes considerably less space and time.

The version of Reactome and all third-party resources are available from the command line and displayed in the command line help.
A set of compressed mapping files are still included in PathwayMatcher to ensure that it can be run upon download, and to facilitate integration in docker and Galaxy. Now, it is further possible for the user to create the static mapping files within the Extractor (github.com/PathwayAnalysisPlatform/PathwayMatcher/tree/master/src/main/java/extractor/#running-extractor), this allows setting the version of the database locally. We added a parameter for the path to the mapping files to be used in the pathway analysis. We however anticipate that this functionality will be used by expert users only.

manuscript / text comments

Findings

Page 5, line 10: The self-citation [1] does not provide support for the statement in the previous sentence, that proteins through biochemical reactions form pathways that interact to form a biological network. However, this statement is so basic that a citation might not be necessary, at all.

Answer: The citation has been removed. However, we disagree with the reviewer that the citation does not support the sentence since the structure of the network formed by pathways and its complexity are precisely the object of this study.

Page 7, Line 53 (Figure 2):
It is not immediately apparent, that counts are cumulative, as this is only mentioned later in the caption. I would suggest the following two minor changes:

Powered by Editorial Manager® and ProduXion Manager® from Aries Systems Corporation

* amend the y-axis label to read: cumulative # publications
* amend the caption start to read: The cumulative number of publications

Answer: The y-axis title has been renamed and the caption updated accordingly.

Page 8, Line 50 (Figure 3):
Two minor changes I would like to suggest:

* correct the caption start from protein to proteoform, to read:
Gene-centric versus proteoform-centric representation
* Gene symbols should always be italicized, while protein symbols should always be just plain formatting. Currently, this is not used systematically in this caption, while the main text seems to be fine.

Answer: This has been corrected. Since the legends are in italic, gene names are switched back to roman there, as normally done for italics within italics.

Page 12, Figure 5, panels C and D:
How can a ratio of degrees which are all positive become negative? Or are the ratio values in the inset log10-transformed, like the values in panel D? This should be noted in the axis labelling and the figure caption. To make the panels more accessible, I wouldn't log-transform the values, but only the axes -- as it is done in panel B. In this case, the tick mark labels of ratios in the C inset would correspond to values found in the main text and the tick mark labels in D would correspond to the degree values in panel C. In addition, the colour scale used in panel D, could also be used in the inset in panel C, to further highlight the correspondence.

Answer: The reviewer is correct that the ratio in C is log-transformed and we apologize that this figure was not correctly annotated and described. This has now been corrected. We have also now use the same scaling, representation, and coloring throughout the elements of the panel. We thank the reviewer for these suggestions that greatly improved the figure.

Methods

Proteoform matching

The description of the proteoform matching types was very hard to follow, especially the part starting page 19, line 5 and running until page 22, line 1. I would remove redundancies between the different matching types, to make this section more readable. In order to make every definition only once, the following reasoning flow seems the most straightforward to me:
1. matching of UniProt accessions
2. matching of isoform specifiers (if isoform doesn't exist in Reactome, shouldn't it match the unmodified one as a default? should there be a mode for that?)
3. PTM matching:
   1. coordinate matching
   2. type matching
4. explain the three non-strict matching types and that they can all be invoked with or without considering PTM type information
5. describe how the strict matching differs from the other matching types

Table 1: The input reference combinations 18-17, 9-13 and 17-13 do not add any information, I would remove them for a quicker overview and only keep the important corner cases. Also, Table 1 is not referenced in the

text, but probably should be in the description of PTM coordinate matching.

Answer: We thank the reviewer for suggestions on how to improve this section. It has been rewritten accordingly.

Mapping omics data to pathways

Page 23, line 50: The link in parentheses suggests to be the source of the Reactome database, while this is only a tool to download it -- as described at: <https://reactome.org/dev/graph-database>. I would prefer having the proper citation of the database here (currently reference [22])

Tables 2 and 3: These do not really add to the text, so I would skip them altogether or reduce them to something like 2-3 entries each.

Answer: This link has been replaced. The tables have been relocated to a summary statistics wiki page and are referred to in the results section.
github.com/PathwayAnalysisPlatform/PathwayMatcher/wiki/Summary-statistics

References

* Reference 13 is a duplicate of 6.
* Reference 14 is a duplicate of 3.

Answer: This has been corrected.

Reviewer #2:
The manuscript entitled "PathwayMatcher: proteoform-centric network construction enables fine-granularity multi-omics pathway mapping" by Sánchez et. al describes a new paradigm to build networks for human biomedical data based on proteoforms including PTMs rather than centering on gene. Developed algorithm relies on Reactome knowledgebase database for proteoform interactions. This manuscript has originality and covers an interesting topic for multi-omics field. I have no doubts that this application will be of great interest for OMICS users. It is important to highlight this review is from the viewpoint of a potential user, since I am a researcher that works with proteomics rather than an expert in application developer. Therefore, I lack the expertise to evaluate the technical algorism issues and I hope other revi-ewers with this expertise will bring more valuable suggestions on this matter. Regarding the use of PathwayMatcher, the Galaxy version seems user friendly and intuitive. However, in my experience was not straightforward when I tried. It is essential to have a better tutorial for users to get the output results as reactions & pathways, over-representation and network view as illustrated in figure 4 of the manuscript. In case users have to login to have full access, this information should be clear. In addition, the local installation shows a major concern. Even though I had installed the Java as suggested in the website instructions I could not execute the jar file. The error was "could not find or load main class". Since, this local installation is an option in additional to the galaxy version, it would be helpful to have a better description in the website regarding possible troubleshoots to guide new users.

Answer: We thank the reviewer for this positive assessment for our work. We have now extended the documentation, and notably added more details on how to get started and how to work with the output. We apologize for the issues with the local installation, the command line should run as simply as in Bioconda or Galaxy. We have corrected potential issues and extended the documentation to prevent problems with the local

Powered by Editorial Manager® and ProduXion Manager® from Aries Systems Corporation

	installation.<br><br>The suggestions pointed by this reviewer were here in order to improve users' accessibility since I believe and hope that PathwayMatcher will be widely used in OMICS field.<br>Minor points:<br>-> This reviewer believes that authors used the term "isoform" sometimes to do not overwrite the correct term "proteoform". However, I strongly suggest using only proteoform throughout the manuscript since it is the most acceptable term nowadays.<br><br>Answer: We agree with the reviewer that isoforms and proteoforms are two different concepts and have thoroughly checked the manuscript that the wording is correct.<br><br>-> I suggest the author to include a zoom-in on fig 3B to highlight the proteoforms (including PTMs) in the red nodes regarding TP53 gene.<br><br>Answer: We thank the reviewer for this suggestion, the different nodes are now annotated as suggested.<br><br>-> There are several proteoforms that does not have the interaction information. How often will be PathwayMatcher updating the database? Will it be based on Reactome update? Please indicate in the manuscript.<br><br>Answer: PathwayMatcher is updated at every release of Reactome, bug fix, and new feature implementation.<br>Furthermore, the code has now been extended so that users can generate the mapping files for PathwayMatcher from a specific version of Reactome. Then the program can be executed with an extra parameter stating the location of the self-generated mapping files. We expect this feature to be of interest to expert users. Instructions on how to do this are given in the wiki:<br>github.com/PathwayAnalysisPlatform/PathwayMatcher/tree/master/src/main/java/extractor/#running-extractor<br><br>-> For consistency, the MOD number for all modifications represented in Fig. 8 (x-axis) should be included.<br><br>Answer: This has been fixed.<br><br>-> The phrase "PathwayMatcher is developed to be a hypothesis generation tool, helping to navigating large datasets and guide experiments. It is not a validation or mechanism inference tool" written in Methods section should be included in the main body text as many readers may first recognize this as a potential tool to understand biological mechanisms.<br><br>Answer: We agree with the reviewer and apologize for this inconsistency in the manuscript. This consideration has now been moved to the discussion and made more prominent.
**Additional Information:**	
**Question**	**Response**
Are you submitting this manuscript to a special series or article collection?	No
**Experimental design and statistics**<br><br>Full details of the experimental design and statistical methods used should be given	Yes

in the Methods section, as detailed in our Minimum Standards Reporting Checklist. Information essential to interpreting the data presented should be made available in the figure legends.<br><br>Have you included all the information requested in your manuscript?	
**Resources**<br><br>A description of all resources used, including antibodies, cell lines, animals and software tools, with enough information to allow them to be uniquely identified, should be included in the Methods section. Authors are strongly encouraged to cite Research Resource Identifiers (RRIDs) for antibodies, model organisms and tools, where possible.<br><br>Have you included the information requested as detailed in our Minimum Standards Reporting Checklist?	Yes
**Availability of data and materials**<br><br>All datasets and code on which the conclusions of the paper rely must be either included in your submission or deposited in publicly available repositories (where available and ethically appropriate), referencing such data using a unique identifier in the references and in the "Availability of Data and Materials" section of your manuscript.<br><br>Have you have met the above requirement as detailed in our Minimum Standards Reporting Checklist?	Yes

Manuscript with DOI
Click here to view linked References
Click here to access/download;Manuscript;Hernandez et al. PathwayMatcher_31.05.19 w. DOI.docx

PathwayMatcher: proteoform-centric network construction enables fine-granularity multi-omics pathway mapping

Luis Francisco Hernández Sánchez[1,2,3] (luis.sanchez@uib.no), Bram Burger[4,5] (bram.burger@uib.no), Carlos Horro[4,5] (carlos.horro@uib.no), Antonio Fabregat[3] (fabregat@ebi.ac.uk), Stefan Johansson[1,2] (stefan.johansson@uib.no), Pål Rasmus Njølstad[1,6] (pal.njolstad@uib.no), Harald Barsnes[4,5] (harald.barsnes@uib.no), Henning Hermjakob[3,7] (hhe@ebi.ac.uk), and Marc Vaudel[1,2,] (marc.vaudel@uib.no)*

[1] K.G. Jebsen Center for Diabetes Research, Department of Clinical Science, University of Bergen, Norway

[2] Center for Medical Genetics and Molecular Medicine, Haukeland University Hospital, Bergen, Norway

[3] European Molecular Biology Laboratory, European Bioinformatics Institute (EMBL-EBI), Wellcome Genome Campus, Hinxton, Cambridge, United Kingdom

[4] Proteomics Unit, Department of Biomedicine, University of Bergen, Bergen, Norway

[5] Computational Biology Unit, Department of Informatics, University of Bergen, Bergen, Norway

[6] Department of Pediatrics, Haukeland University Hospital, Bergen, Norway

[7] Beijing Proteome Research Center, National Center for Protein Sciences Beijing, Beijing, China

* To whom correspondence should be addressed

Abstract

Background

Mapping biomedical data to functional knowledge is an essential task in bioinformatics and can be achieved by querying identifiers, e.g. gene sets, in pathway knowledgebases. However, the isoform and post-translational modification states of proteins are lost when converting input and pathways into gene-centric lists.

Findings

Based on the Reactome knowledgebase, we built a network of protein-protein interactions accounting for the documented isoform and modification statuses of proteins. We then implemented a command line application called PathwayMatcher (github.com/PathwayAnalysisPlatform/PathwayMatcher) to query this network. PathwayMatcher supports multiple types of omics data as input, and outputs the possibly affected biochemical reactions, subnetworks, and pathways.

Conclusions

PathwayMatcher enables refining the network-representation of pathways by including proteoforms defined as protein isoforms with post-translational modifications. The specificity of pathway analyses is hence adapted to different levels of granularity and it becomes possible to distinguish interactions between different forms of the same protein.

Keywords: Pathway, post-translational modification, network, proteoform

Findings

In biomedicine, molecular pathways are used to infer the mechanisms underlying disease conditions and identify potential drug targets. Pathways are composed of series of biochemical reactions, of which the main participants are proteins, that together form a complex biological network. Proteins can be found in various forms, referred to as proteoforms [1]. The different proteoforms that can be obtained from the same gene/protein depend on the individual genetic profiles, on sequence cleavage and folding, and on post-translational modification (PTM) states[2]. Proteoforms can carry PTMs at specific sites, conferring each proteoform unique structure and properties [3]. Notably, many pathway reactions can only occur if all or some of the proteins involved are in specific post-translational states.

However, when analyzing omics data, both input and pathways are summarized in a gene- or protein-centric manner, meaning that the different proteoforms and their reactions are grouped by gene name or protein accession number, and the fine-grained structure of the pathways is lost. One can therefore anticipate that proteoform-centric networks provide a rich new paradigm to study biological systems. But while gene networks have proven their ability to identify genes associated with diseases [4], networks of finer granularity remain largely unexplored.

Here, we present PathwayMatcher, an open-source standalone application that considers the isoform and PTM status when building protein networks and mapping omics data to pathways from the Reactome database. Reactome [5], is an open-source curated knowledgebase consolidating documented biochemical reactions categorized in hierarchical pathways, and notably includes isoform and PTM information for the proteins participating in reactions and pathways.

As an example of the complexity of hierarchical pathway information, we provide a graph representation of *Signaling by NOTCH2* from Reactome (**Figure 1**). This pathway is a sub-pathway of the pathways *Signaling by NOTCH* and *Signal Transduction*. It is composed of two

sub-pathways (*NOTCH2 intracellular domain regulates transcription* and *NOTCH2 Activation and Transmission of Signal to the Nucleus*), comprising 32 and 54 reactions, yielding 28 and 141 edges, respectively. The 31 participants of the *Signaling by NOTCH2* pathway are also involved in reactions in other pathways, between themselves and with 2,055 other proteins, resulting in 6,525 external edges. Note that in this pathway, Cyclic AMP-responsive element-binding protein 1 (coded by *CREB1*) is phosphorylated at position 46 (labeled as *CERB1_P* in **Figure 1**) and Neurogenic locus notch homolog protein 2 (coded by *NOTCH2*), is found in three forms (unmodified and with two combinations of glycosylation, labeled as NOTCH2, NOTCH2_Gly1, and NOTCH2_Gly2, respectively).

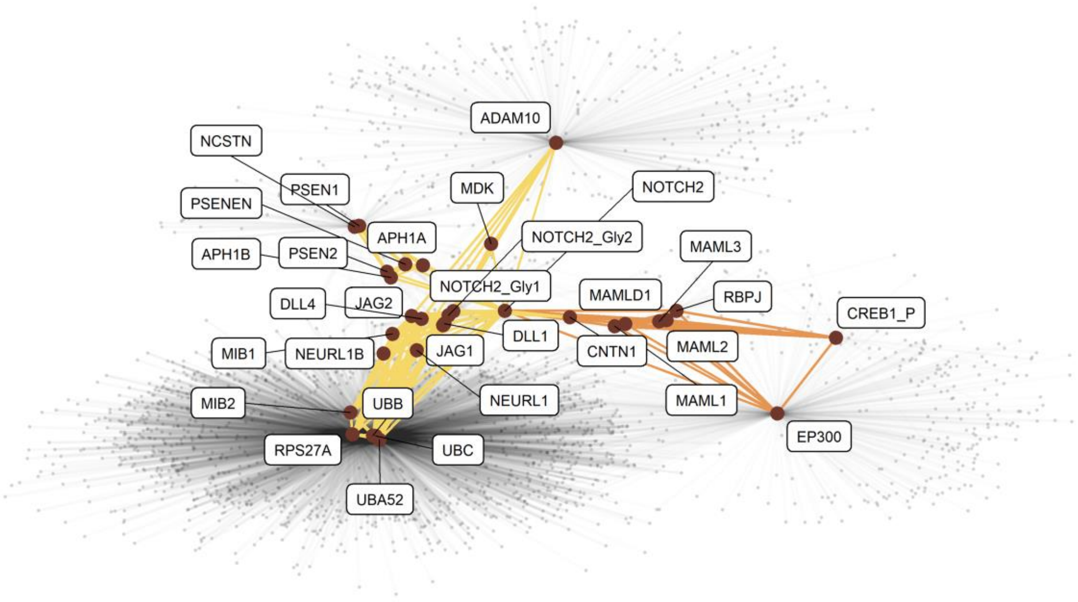

Figure 1: Graph representation of the Signaling by NOTCH2 *pathway as extracted from the Reactome database. Participating proteins are displayed as large dark red dots labeled with their canonical gene name. Post-translational modifications (PTMs) are indicated with suffixes in the label. A connection between two dots indicates a documented interaction between the two proteins in the given pathway. Connections belonging to the sub-pathways* NOTCH2 intracellular domain regulates transcription *and* NOTCH2 Activation and Transmission of Signal to the Nucleus *are displayed in orange and yellow, respectively. The interactions involving these proteins in other pathways are displayed with light gray connections in the background.*

The amount of information available on reactions involving modified proteins has dramatically increased during the past two decades (**Figure 2**), with 3,947 and 5,631 publications indexed in Reactome (version 64 at time of writing) describing at least one reaction between modified proteins or between a modified and an unmodified protein, respectively. To harness this vast amount of knowledge, we built a network representation of pathways that we refer to as *proteoform-centric*, where protein isoforms with different sets of PTMs are represented with different nodes, in contrast to *gene-centric* networks, where one node is used per gene name or protein accession. In this representation, two proteoforms are connected if they participate in the same reaction. Note that proteoforms can participate in reactions both individually and as part of a set or complex. Furthermore, they can have four different roles: input, output, catalyst, or regulator.

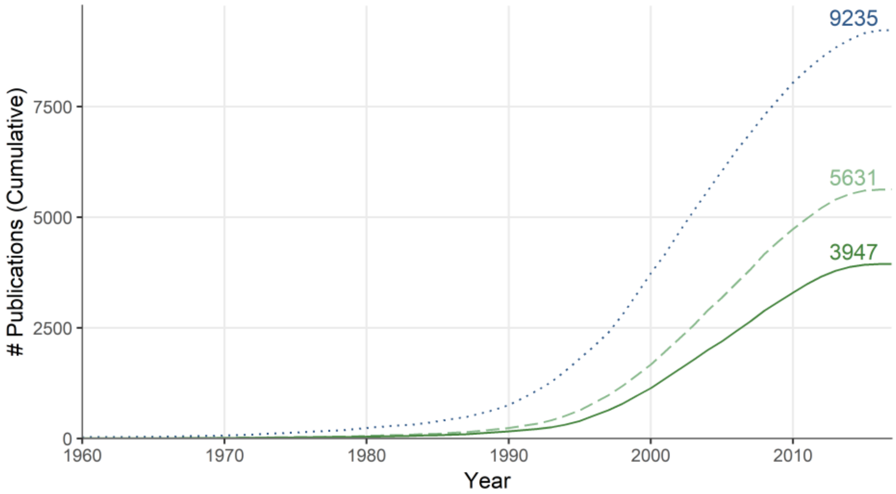

Figure 2: The cumulative number of publications indexed in Reactome documenting at least one reaction between two proteins with PTMs (solid dark green line), between one protein with PTMs and one without (dashed light green line), and two proteins without PTMs (dotted blue line), counting all publications with a year earlier than or equal to the x-axis value. The number of publications in each category at time of writing is indicated to the right.

The fundamental difference between gene- and proteoform-centric networks is illustrated in **Figure 3**, showing the graph representation of interactions with the protein *Cellular tumor antigen p53* (P04637) from the *TP53* gene. In a gene-centric paradigm (**Figure 3A**), 221 nodes

are connected to a single node, making 220 connections; while in a proteoform-centric network (**Figure 3B**), 227 proteoforms connect to 23 proteoforms coded by *TP53* making 414 connections. Note that the proteoforms coded by *TP53* are themselves involved in reactions, making 24 *TP53-TP53* connections. In this example, the proteoform-centric network thus presents more nodes and connections than the gene-centric network, with visible structural differences in the network organization. We hypothesize that the proteoform-centric network paradigm depicted in **Figure 3B** provides a rich map that will enable navigating biomedical knowledge to a higher level of detail, to better assess the effect of perturbations, and identify drug targets more specifically.

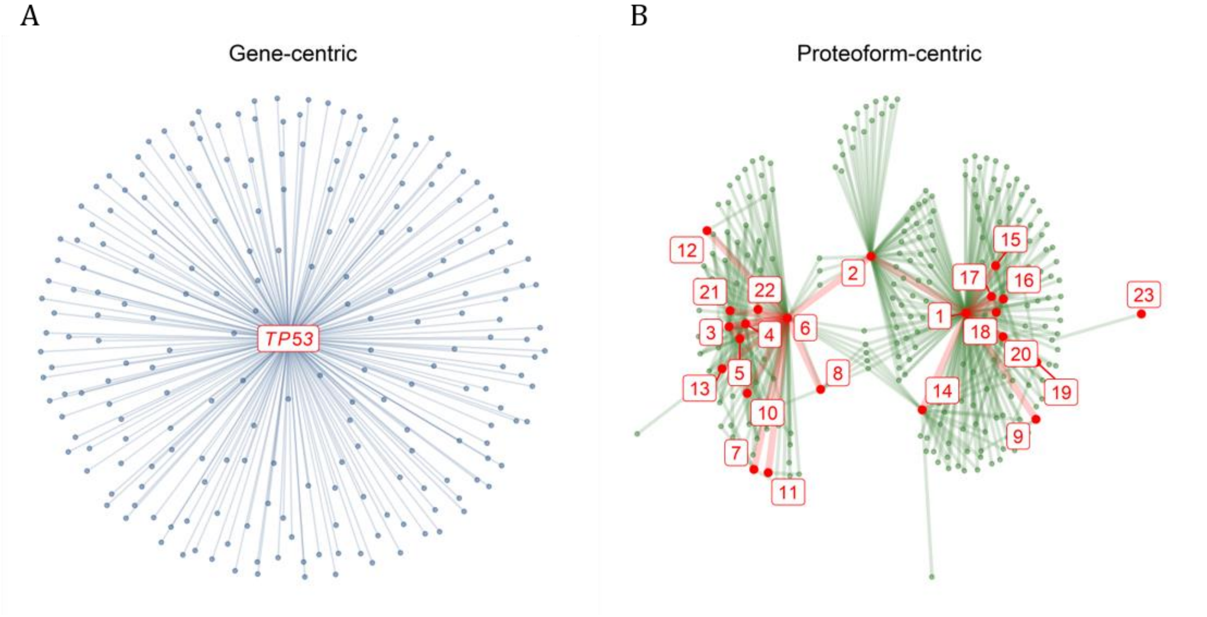

Figure 3: Gene-centric versus *proteoform-centric representation. (A) Graph representation of the genes involved in reactions (through their corresponding proteins) with (the corresponding proteins of)* TP53, *with a single node per gene.* TP53 *is represented with a red label at the center and genes coding proteins involved in reactions with TP53 are represented with smaller blue dots at the periphery connected to the* TP53 *gene with blue lines. (B) Graph representation of the proteins involved in a reaction with gene products of* TP53, *distinguishing isoforms and post-translationally modified proteins as different proteoforms. The proteoforms coded by* TP53 *and the proteoforms involved in a reaction with them are represented with large red and small green dots, respectively. The proteoforms coded by* TP53 *are numbered according to Table 1. The connections between proteoforms coded by* TP53 *are displayed with thick red lines and connections with other proteoforms with thin green lines.*

#	*Isoform*	*Modifications*
1	Canonical	None

2	Canonical	pS15
3	Canonical	pS15 pS20 aceK120 aceK382
4	Canonical	pS15 pS20 aceK382
5	Canonical	pS15 pS20 aceK120
6	Canonical	pS15 pS20
7	Canonical	pS15 pS20 dimethR335 dimethR337 methR333
8	Canonical	pS15 pS20 ubiK
9	Canonical	pS15 pS33 pS46
10	Canonical	pS15 pS20 pS269 pT284
11	Canonical	pS15 pS20 methK370
12	Canonical	pS15 pS20 methK372
13	Canonical	pS15 pS20 methK382
14	Canonical	ubiK
15	Canonical	pS315
16	Canonical	pT55
17	Canonical	pS15 pS392
18	Canonical	pS37
19	Canonical	dimethK373
20	Canonical	sumoK386
21	Canonical	pS15 pS20 pS46
22	Canonical	pS15 pS20 pS392
23	Canonical	dimethK370 dimethK382

Table 1: Proteoforms of Figure 3B. Only the canonical isoforms are annotated to date, as indicated in the second column. The post-translational modification status is indicated in the third column with modification short name and modification site when annotated. Abbreviations: pS: O-phospho-L-serine; pT: O-phospho-L-threonine; aceK: N6-acetyl-L-lysine; dimethR: symmetric dimethyl-L-arginine; dimethK: N6,N6-dimethyl-L-lysine; methR: omega-N-methyl-L-arginine; methK: N6-methyl-L-lysine; ubiK: ubiquitinylated lysine; sumoK: sumoylated lysine.

PathwayMatcher allows the user to tune the granularity of the network representation of pathways by representing nodes as (i) gene names, (ii) protein accession numbers, or (iii) proteoforms, and supports the mapping of multiple types of omics data: (i) genetic variants, (ii) genes, (iii) proteins, (iv) peptides, and (v) proteoforms. Genetic variants are mapped to proteins using the Ensembl Variant Effect Predictor [6], gene names are mapped to proteins using the UniProt identifier mapping [7], and peptides are mapped to proteins using PeptideMapper [8]. If a peptide maps to different proteins, all possible proteins are considered for the search and protein inference must be conducted *a posteriori* [9]. If peptides are modified, they are mapped to the proteoforms presenting compatible PTM sets. Proteins are mapped to the pathway network using their accession, while proteoforms are mapped by comparing their protein accession, isoform number, and PTM set. A schematic representation of the

PathwayMatcher matching procedure is shown in **Figure 4**. More details on the mapping procedure, formats, and settings can be found in the methods section and in the online documentation (github.com/PathwayAnalysisPlatform/PathwayMatcher/wiki). For more information on how the pathway representation is constructed from the different external resources, please consult the methods section and the online documentation (github.com/pathwayanalysisplatform/pathwaymatcher/tree/master/src/main/java/extractor).

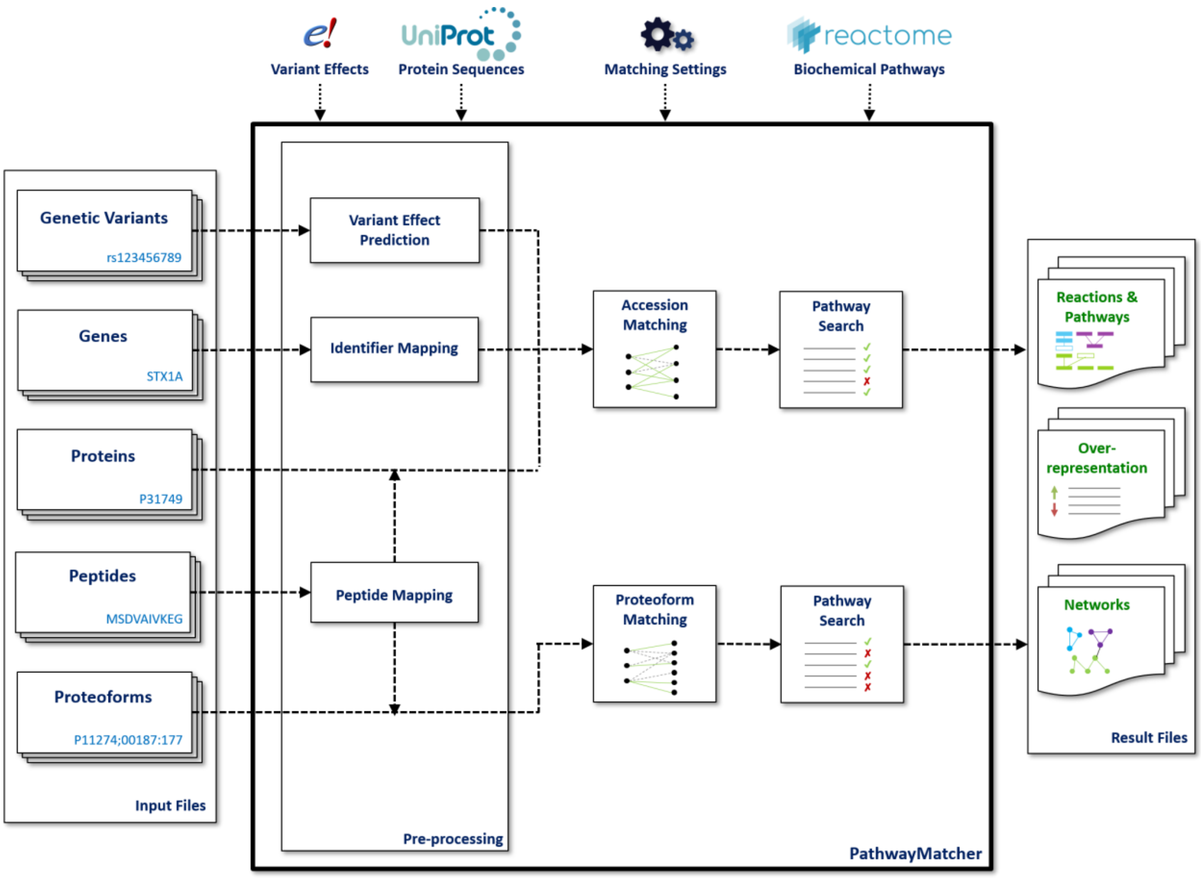

Figure 4: Schematic representation of the PathwayMatcher matching procedure. Input of various types is modelized as sets of proteins or proteoforms based on the annotation of isoforms and PTMs. Proteins and proteoforms are then mapped to Reactome based on user settings. Matched reactions and pathways, the results of an over-representation analysis, and sub-networks generated from the input are exported as text files.

PathwayMatcher produces three types of output: (i) the result of the matching, listing all possible reactions and pathways linked to the input; (ii) the results of an over-representation analysis; and (iii) networks in relationship with the input. The over-representation analysis is

performed on the pathways matching and follows the first generation of pathway analysis methods [10], *i.e.* a *p*-value for each pathway in the reference database is calculated using a binomial distribution followed by Benjamini-Hochberg correction [11] (in a similar way as performed by the Reactome online analysis tool [5]). If the input can be mapped to proteoforms, the over-representation analysis is conducted using a proteoform-centric representation of pathways, using proteins otherwise. The exported networks represent the internal and external connections that can be drawn from the input, where internal connections connect two nodes from the input list, and external connections one node from the input list to any node not in the input. The user can select to export these networks using nodes defined as genes, proteins, or proteoforms. Connections between nodes in the network are annotated with information on whether they participate as complex or set, and their role in the reaction.

As displayed in **Figure 5A**, 68% of the pathways present at least one proteoform-specific participant, *i.e.* with isoform or PTM annotation. The number of pathways containing a given gene product or proteoform is displayed in **Figure 5B**, showing how using proteoforms allows distinguishing pathways more specifically than genes, with a median of four pathways matched per proteoform compared to eleven pathways per gene. When the input can be mapped to proteoforms, PathwayMatcher can restrict the search for reactions and pathways to those that specifically involve proteins in the desired form, hence reducing the number of possible connections for a given node in the resulting network. Conversely, the proteoform-centric network representation allows identifying interactions between multiple proteoforms originating from the same gene or protein, resulting in new connections compared to a gene-centric representation.

Figure 5C shows that the number of connections per proteoform is lower than the number of connections for the respective gene for most proteoforms, varying from 300-fold decrease to 10-fold increase. Interestingly, plotting the number of connections of a proteoform in gene-centric or proteoform-centric networks shows that the largest gene-centric hubs, corresponding to five genes, decompose into 127 proteoforms that do not outlie the distribution of the number

of connections in the proteoform network (**Figure 5D**). Conversely, a group of 484 densely connected outliers emerges from 44 genes.

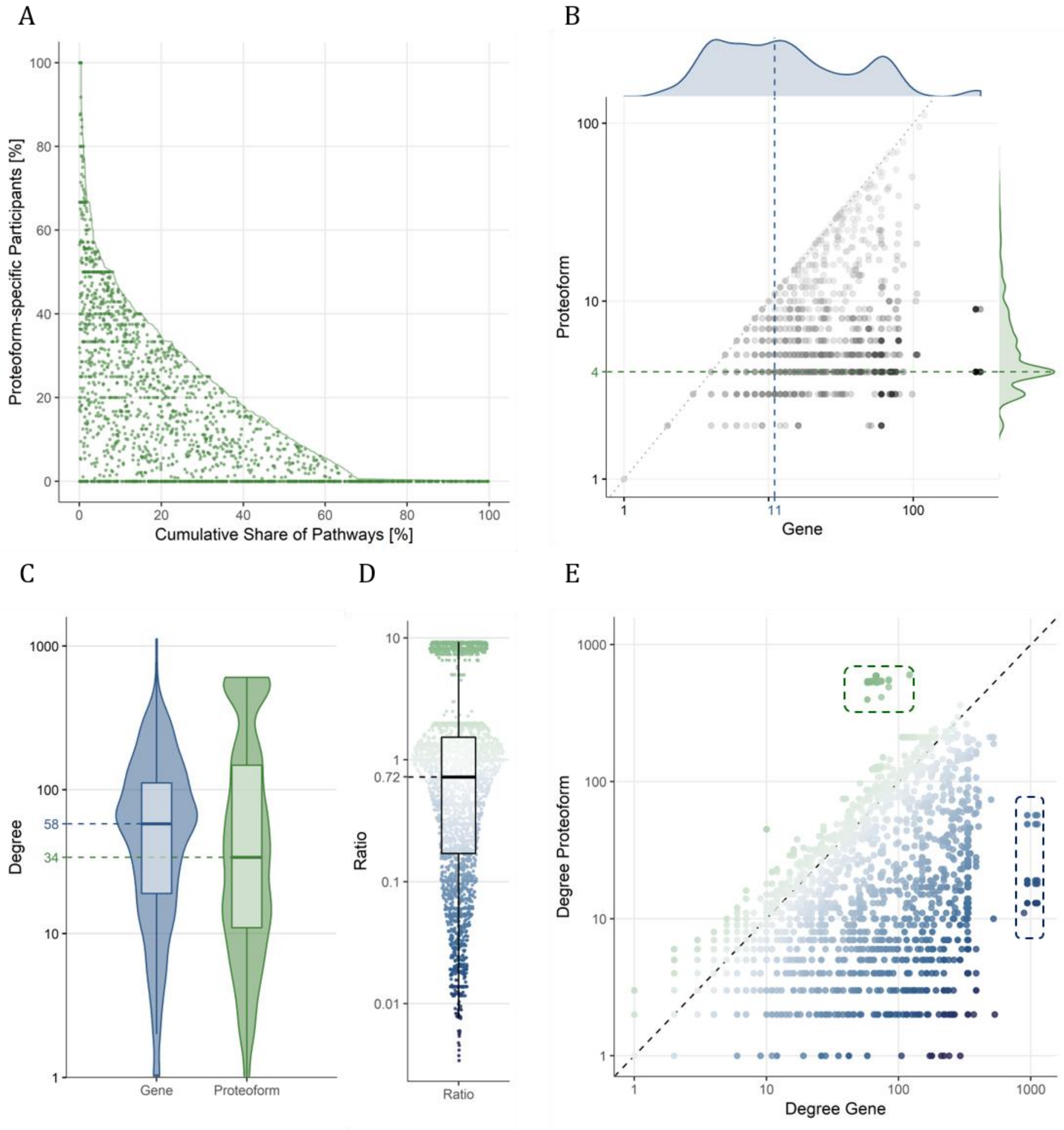

Figure 5: Prevalence of proteoforms in pathways. (A) The share of proteoform-specific participants in a pathway, i.e. *proteins that are annotated with isoform and/or PTM information, is plotted against the cumulative share of pathways, going from the highest share of proteoforms to the lowest. The cumulative share of pathways is displayed with a solid green line. The share of proteoform-specific participants in each pathway is plotted with a green dot with a jitter on the x-axis between zero and the solid line. (B) For all proteoform-specific participants, the number of pathways mapped using the proteoform versus gene is plotted in black. The density of the number of pathways mapped are indicated at the top (blue*

) and right (green) for gene and proteoform matching, respectively. The median number of pathways mapped is indicated with dashed lines. (C) The violin and box plots of the degree, i.e. number of connections, for the proteoform-specific participants in a gene-centric or proteoform-centric network are plotted to the left (blue) and right (green), respectively. (D) The ratio of degrees, proteoform over gene, is plotted with a blue-grey-green gradient with the box plot overlaid in black. (E) The degree of the proteoform-specific participants in the proteoform-centric network is plotted against the degree in the gene-centric network. Dots are colored with a blue-grey-green gradient corresponding to the ratio in D. Outliers of high degree in the gene-centric but not in the proteoform-centric network are indicated with blue dashes to the right. Outliers of high degree in the proteoform-centric but not in the gene-centric network are indicated with green dashes to the top. Note that base 10 logarithmic scales are used for the axes in B, C, D, and E.

In order to fully benefit from the gain in specificity of the proteoform-representation of pathways, it is necessary to exactly match the representation of proteoforms in Reactome. Any mismatch between the input data and the database would result in a loss of sensitivity. In practice, such mismatches can result from an incomplete proteoform representation in Reactome, where only the minimal set of modifications necessary to perform a reaction are annotated. Conversely, input data can present unresolved isoform, missing modifications or inaccurate localization, especially in the case of bottom-up proteomics [12]. Since the size of the proteoform network is unknown to date, the effect of missing annotations in the database is not directly quantifiable.

To estimate the sensitivity of the matching, we mapped the phosphoproteome from Ochoa *et al.* [13] to Reactome using PathwayMatcher: among the 10,588 accessions representing phosphoproteins, 5,519 (52%) could be matched to an accession in Reactome, while among the 116,258 phosphosites reported, only 654 (<1%) could be matched exactly in Reactome. Accession matching is equivalent in terms of sensitivity and specificity to a gene-centric representation of pathways, while strict proteoform matching, requiring exact isoform and modification set, maximizes specificity at the cost of sensitivity.

In order to mitigate the sensitivity loss while maintaining specificity, we implemented multiple types of matching that present different levels of stringency, as detailed in the methods: (i) *One*, (ii) *One without PTM types*, (iii) *Superset*, (iv) *Superset without PTM types*, (v) *Subset*, (vi) *Subset without PTM types*, and (vii) *Strict*. Table 2 lists the share of phosphosites that can be matched to

a proteoform in Reactome when querying the accession with a phosphorylation at the given site, and only at this site, with a tolerance of five amino acids. There, one can see that increasing the stringency of the matching dramatically reduces the sensitivity. Since both Reactome and the list of phosphosites represent a minimal set of modifications, the *Strict* matching is overly selective, while *Accession* and *Superset* include reactions where the proteins are not modified.

Subset and *One* represent the coverage of the input by Reactome. Here, *Subset* and *One* are equivalent because the input consists of single phosphosites. In a data set containing combinations of phosphosites, *Subset* would match proteoforms taking phosphosite combinations into account, while *One* would represent any proteoform with at least one matching phosphosite. The increased number of matches without PTM type can be imputed to mismatching PTM identifiers, or the presence of other PTMs at the input sites or at neighboring positions.

Matching Type	Share of Phosphosites Matched
Accession	57.44%
Superset without PTM types	56.38%
Superset	56.33%
One without PTM types	6.01%
Subset without PTM types	6.01%
One	1.27%
Subset	1.27%
Strict	0.15%

Table 2: Share of the phosphosites from Ochoa et al. [13] matching to Reactome using different matching types. Proteoforms were constructed by adding a phosphorylation at the given site, and only at this site, and were queried against Reactome. The percentage of proteoforms matched is provided in the second column. A tolerance of five amino acids was used on the modification site. More details on this analysis can be found in the Methods section.

To illustrate the difference induced by each matching type on the proteoform matching, we calculated the percentage of proteoforms matched with selected example proteoforms. In Error! Reference source not found., we present two of the example proteoforms, one from Insulin (P01308) and one from Mitogen-activated protein kinase kinase kinase 7 (MAP3K7). Insulin and *MAP3K7* have five and seven different proteoforms annotated in Reactome, four and six of them with PTM annotation, respectively. By design, the *Strict* matching type matches only the original proteoform while the accession matching matches all proteoforms. The other matching types allow balancing between the two stringencies, and displayed variying levels of specificity for those proteoforms.The results show that relaxing the stringency of the matching rapidly induces a loss in specificity due to the similarity of the different proteoforms of a given gene or protein.

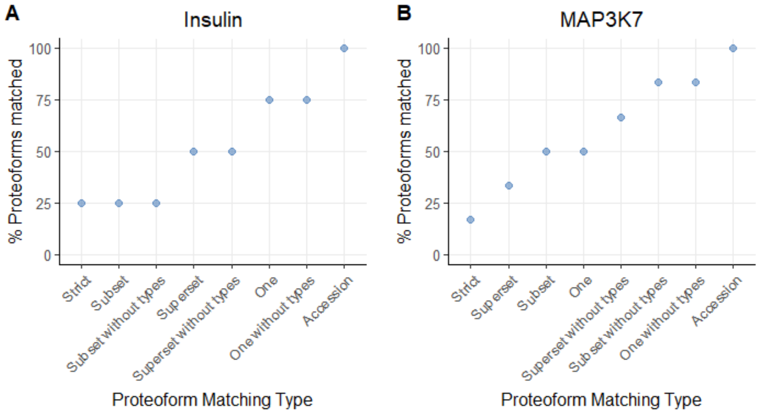

Figure 6: Two examples of proteoforms showing the proteoform matching results for each matching type. (A) Proteoform P01308;MOD:00087:53,MOD:00798:31,MOD:00798:43, from insulin (P01308), is matched against all modified proteoforms of insulin in Reactome. (B) Proteoform O43318;MOD:00047:184,MOD:00047:187, from "Mitogen-activated protein kinase kinase kinase 7" (MAP3K7)*, is matched against all modified proteoforms of* MAP3K7 *in Reactome.*

Furthermore, we randomly selected proteoforms in Reactome and altered them by changing the type and localization of the PTMs to simulate mismatching or missing information, and the altered proteoforms were matched to Reactome, see details in the Methods section. In this setup, the share of altered proteoforms that can be recovered using the different matching types, referred to as *Original* matches, providing an estimate of the matching sensitivity in case

of incomplete or mismatching proteoform definition. Conversely, the share of other proteoforms matching despite not being originally selected, referred to as *Other* matches, provides an estimate of the error rate, the complement of specificity.

Error! Reference source not found. shows the percentage of proteoforms that matched at least one proteoform in the database separated on matching type. As expected, accession matching displays the highest sensitivity at the lowest specificity, while the *Strict* and *Subset* matching display the highest specificity at lowest sensitivity. The *Superset* matching presented low sensitivity and low specificity, while the *One* matching presented a balance between specificity and sensitivity. Finally, the matching with no types presented similar trends but with almost maximum sensitivity and lower specificity. Together, these results show how relaxing the matching stringency allows balancing between sensitivity and specificity, and demonstrate the importance of accurate proteoform definition in both the input and the reference knowledgebase.

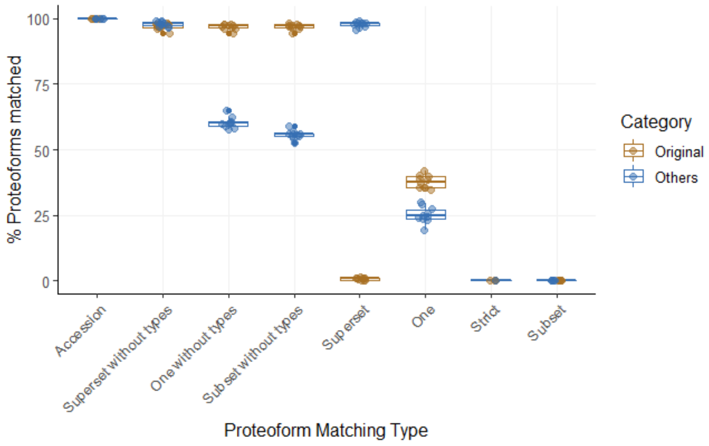

Figure 7: Percentage of proteoforms with at least one proteoform match in the database with each matching criteria. The total candidate proteoforms available are separated in two categories, the original and others. Original is the proteoform in the database that was modified for the sampling, while Others are the proteoforms that share the same protein accession.

Through its paradigm-shift, PathwayMatcher hence provides a fine-grained representation of pathways for the analysis of omics data. However, this comes at the cost of increased

complexity: gene-centric networks comprise a limited number of nodes, approximately 20,000 for humans, whereas in a proteoform-centric paradigm, the human network is expected to have several million nodes [2]. With the current version of Reactome, building the gene- and proteoform-centric networks results in 9,759 and 12,775 nodes with 443,229 and 672,047 connections, respectively. We classified the nodes into two categories, canonical or specific gene products, depending on whether or not they represent the unmodified canonical isoform of a protein according to UniProt. Within the proteoform network, 432,169 connections between 9,694 nodes link two canonical gene products, 95,539 connections between 7,734 nodes involved one canonical and one specific gene product, and 2,806 nodes with 144,339 connections involved two specific gene products. More summary statistics on the underlying network can be found in the wiki of the PathwayMatcher repository.

In addition to the increased size of the underlying network, matching proteoforms requires comparing isoforms and sets of modifications, possibly with tolerance and wildcards for the modification definition and localization, which is computationally much more intensive than simply comparing identifiers. **Figure 8** shows the performance of PathwayMatcher benchmarked against public data sets of (A) genetic variants, (B) proteins, (C) peptides, and (D) proteoforms.

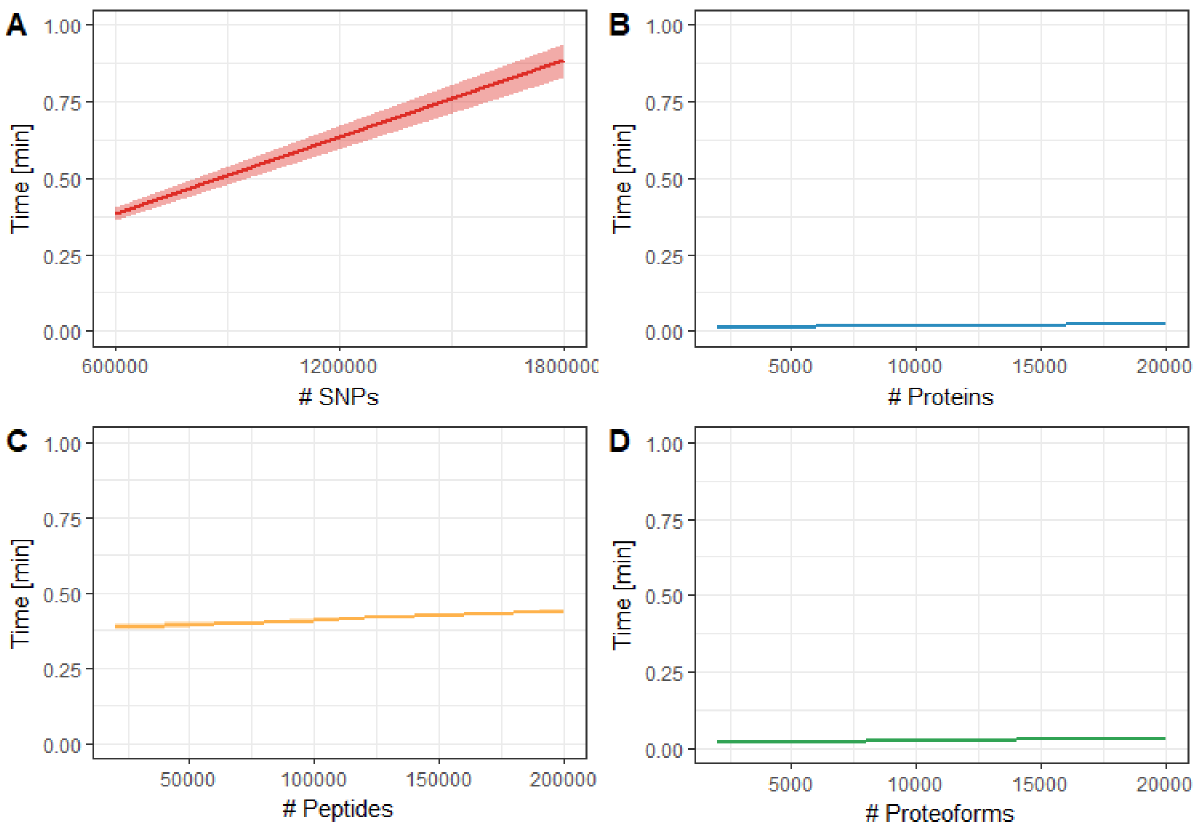

Figure 8: Performance of PathwayMatcher using (A) genetic variants as single-nucleotide polymorphisms (SNPs), (B) proteins, (C) peptides and (D) proteoforms. Performance in minutes is plotted against input size. The mean is displayed as a solid line and the 95% range as a ribbon (only visible in (A) due to the high reproducibility in other cases).

For the proteins and proteoforms, the processing time increased linearly related to the query size with a small slope, making it possible to search all available proteins within a few seconds. As expected, protein identifiers provided the fastest response time, while proteoforms were the second fastest. Mapping peptides took approximately 30 seconds more, corresponding to the indexing time of the protein sequences database by PeptideMapper [8], after which the time increased linearly in a similar fashion as for proteins. For the genetic variants, an extra mapping step is required to map possibly affected proteins, adding additional computing time. The overall mapping time for a million single-nucleotide polymorphisms (SNPs) was less than a minute, which is acceptable compared to the other steps of a variant analysis pipeline. Note that the processing time was very reproducible across runs, where minor variation is only noticeable using genetic variants, resulting in very thin ribbons in **Figure 8B-D**.

In conclusion, PathwayMatcher is a versatile application enabling the mapping of several types of omics data to pathways in reasonable time and can readily be included in bioinformatic workflows. It is important to underline that PathwayMatcher maps experimental data to pathways in a systematic and unbiased fashion, *i.e.* it collects all pathways containing at least one of the participant proteins or proteoforms of the input data and does not perform any filtering or biological inference. Through this process it attempts at minimizing the prevalence of false negatives by considering all the possible pathways annotated in the reference database. It can however not control for missing annotation, *i.e.* what is not annotated in the knowledgebase is not considered.

Furthermore, although PathwayMatcher implements an over-representation analysis module, we recommend that users rather interpret the results of the matching and the resulting networks using the systems biology method that best suits the experiment and biomedical context. Based on generic pathways, PathwayMatcher is not developed as a mechanism inference or validation tool, but as a hypothesis generation tool, helping to navigate large datasets and guide experiments to uncover biological processes relevant to specific research questions.

Thanks to the fine-grained information available in Reactome, PathwayMatcher supports refining the pathway representation to the level of proteoforms. To date, only a fraction of the several million expected proteoforms [2] have annotated interactions, but as the understanding of protein interactions continues to increase, and the ability to identify and characterize them in samples progresses, proteoform-centric networks will surely become of prime importance in biomedical studies. Notably, the effect of genetic variation on genes, transcripts, and proteins is currently only partially resolved for a fraction of the genome. The rapid development of this field will make it possible to identify biological functions affected by variants within the human network. Refining its representation to the level of proteoforms will allow pinpointing more precisely reactions and pathways, and hence increase our ability to understand biological mechanisms and potentially identify druggable targets.

Methods

Implementation

PathwayMatcher is implemented in Java 8.0.

Availability

PathwayMatcher is freely available at github.com/PathwayAnalysisPlatform/PathwayMatcher under the permissive Apache 2.0 license. It is also possible to use PathwayMatcher as a Docker image: hub.docker.com/r/lfhs/pathwaymatcher. PathwayMatcher can be obtained from the Bioconda channel of the Conda [14] package manager at bioconda.github.io/recipes/pathwaymatcher/README.html. Finally, PathwayMatcher is available as a Galaxy [15] tool in the Galaxy ToolShed [16] at toolshed.g2.bx.psu.edu/view/galaxyp/reactome_pathwaymatcher where it can be readily integrated into analysis workflows. PathwayMatcher has also been installed into the public European Galaxy instance, usegalaxy.eu, making it possible to use the application without requiring any local configuration and just providing valid input files and options. The complete URL for the online tool is:

https://usegalaxy.eu/?tool_id=toolshed.g2.bx.psu.edu%2Frepos%2Fgalaxyp%2Freactome_pathwaymatcher%2Freactome_pathwaymatcher

Upon installation, PathwayMatcher can be used from the command line to query Reactome using various types of omics data. Either the ".jar" file is run directly using Java or the Docker image is instantiated to a container. Detailed information on implementation, installation, usage and format specifications is available in the online documentation at github.com/PathwayAnalysisPlatform/PathwayMatcher/wiki.

Input and Output

Detailed and updated documentation of the input and output can be found in the online documentation at github.com/PathwayAnalysisPlatform/PathwayMatcher/wiki.

As schematized in **Figure 9**, a simple representation is used for proteoforms: (i) the UniProt protein accession and (ii) the set of PTMs separated by a semicolon ';'. The protein accession can include the isoform number specified with a dash '-'. The PTM set contains each PTM separated by a comma ','. Each PTM is specified using a modification identifier and a site, separated by a colon ':'.

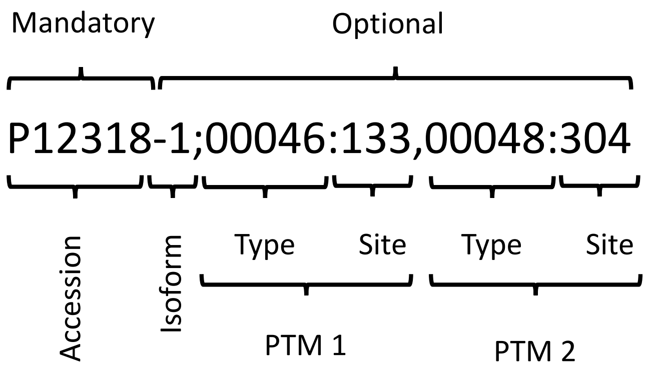

Figure 9 : Example of proteoform notation, composed of a protein accession, an isoform number, and a set of PTMs.

Note that the order of PTMs do not affect the search. The PTM identifier is a five digit identifier from the PSI-MOD Protein Modification [17]. The site is an integer specifying the 1-based index of the modified amino acid on the sequence as defined by UniProt. The modification site field is mandatory, *?* or *null* indicate that the position is not known.

It is common to write the identifiers for the PTM types with the prefix 'MOD:' before the five digits of the ontology term. PathwayMatcher also allows the user to write the identifier without the prefix. PathwayMatcher also allows querying all proteoforms modified at a given site using the '00000' wild card for modification type combined with a matching type that does not consider the modification types such as *One* without types or *Subset* without types. For more details, see the *Proteoform matching* subsection .

Post-translational modifications in the Reactome data model

The Reactome object model specifies physical entities, *e.g.* complexes, proteins and small molecules, and proteins are annotated using unique identifiers. These entities participate in reactions in specific cellular compartments. They can also be connected to multiple instances of *Translational Modification* objects, which contain a specific coordinate on the protein sequence and an identifier following the PSI-MOD ontology [17]. The portion of physical entities referring to proteins are associated to other class of objects as reference entities, which contain protein annotations in external databases such as UniProt [18]. Therefore, a proteoform is represented as a physical entity associated to a set of modifications for specific processes at specific subcellular location. Each modification has a PSIMOD ontology identifier as type and an integer coordinate for the site in the peptide sequence where the modification occurs. The coordinate can be *?* or *null* when the site is not known. Reactome annotates 127 different protein modifications for humans, of which Error! Reference source not found. displays the most frequent.

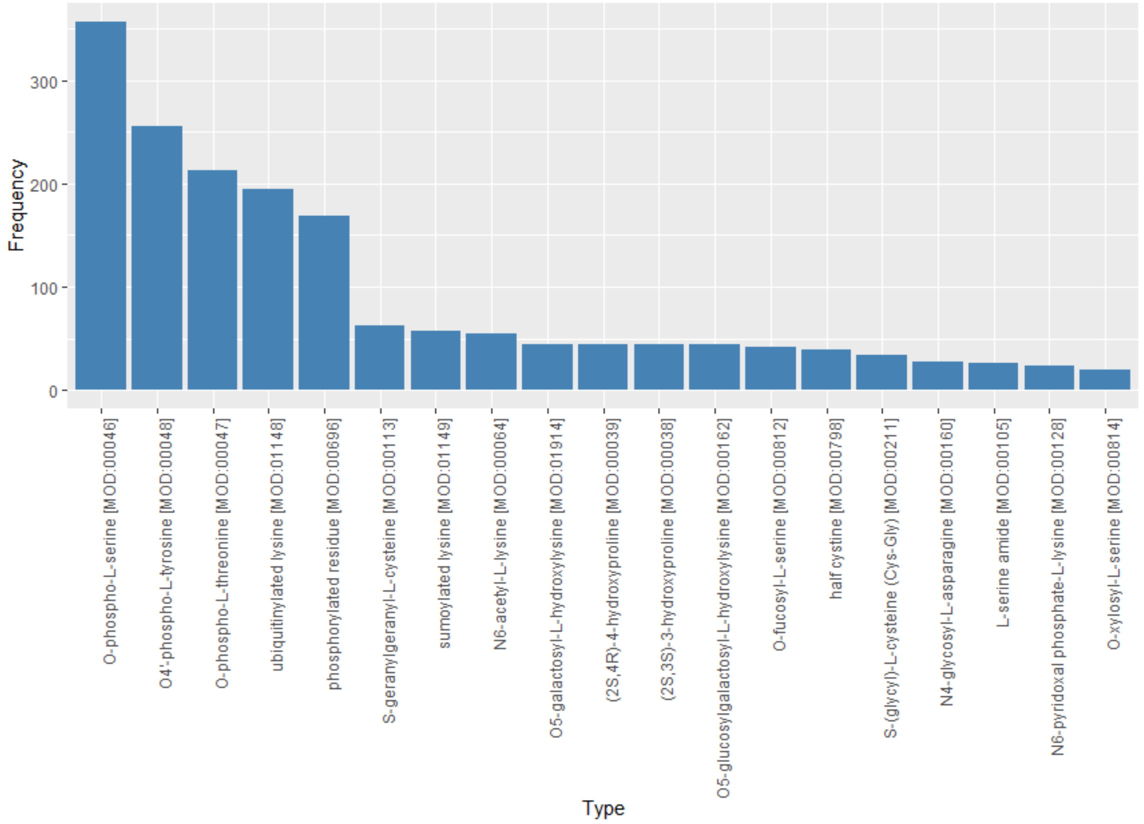

Figure 10: Prevalence of the different PTM annotations in Reactome. PTM labels are extracted from the Reactome database and the number of proteins annotated with the PTM is displayed for each label. If a protein is carrying multiple instances of the PTM, the PTM is counted only once.

Proteoform matching

Searching pathways using gene names or protein accessions solely requires mapping a string of characters between the input and the knowledgebase. In order to map the proteoforms to reactions and pathways, it is necessary to decide if the proteoforms in the input are equivalent to the proteoforms annotated in the reference database, Reactome, taking into account the protein accession, isoform information, and the set of PTMs. Two proteoforms can have all, some, or none of these elements in common. We defined a set of criteria to match two proteoforms, one from the input and one from the reference database. First, identical protein accession and isoform number are required for a match: either both proteoforms are from the canonical isoform (*e.g.* P31749), or from the same isoform (*e.g.* P31749-3). Then, the PTMs carried by each proteoform are compared using the modification type and the modification site on the protein sequence. For two PTMs to match, their modification type as defined by the PSI-

MOD ontology [17] needs to be identical and the distance between their sites must be below a user-provided margin, as detailed in Table 2.

PTM

Different matching types are implemented in PathwayMatcher for the PTM sets:

- *Strict*: the input and reference proteoforms have the same number of PTMs and every PTM of the input proteoform matches a PTM in the reference proteoform.
- *Superset*: every PTM of the reference proteoform matches a PTM of the input proteoform, but some PTMs in the input proteoform may not match PTMs in the reference proteoform.
- *Subset*: every PTM of the input proteoform matches a PTM of the reference proteoform, but some PTMs of the reference proteoform may not match PTMs in the input proteoform.
- *One*: at least one PTM of the input proteoform matches a PTM of the reference proteoform.

In addition, *Superset without PTM types*, *Subset without PTM types*, and *One without PTM types* are identical to *Superset*, *Subset,* and *One*, respectively, but do not account for modification type in PTM matching. Finally, note that for the *Strict* matching, the PTMs match when their sites are exactly identical and no margin is allowed: either both are the same positive integer or both are *null*, or *?*.

For details and examples to run PathwayMatcher with the different matching criteria see the online documentation (github.com/PathwayAnalysisPlatform/PathwayMatcher/wiki/Proteoform-matching).

Additional considerations:

- Negative, zero, or floating-point values are invalid as sequence coordinates in the input.

- The margin to compare the coordinates must be a positive integer.

Input	Reference	Margin	Matched	Comment
17	17	0	Yes	Equal
16	17	0	No	Out of margin
7	13	5	No	Out of margin
8	13	5	Yes	In margin
19	13	5	No	Out of margin
0	2	5	No	Input in margin, but 0 is not a valid coordinate
-1	2	5	No	Input in margin but negative
?, empty, null	c	k	Yes	Input is less specific
c	?, empty, null, -1	k	Yes	Input is more specific
?, empty, null	?, empty, null, -1	k	Yes	Equally unspecific
Negative int, zero	Any	k	No	Negative or zero input are invalid

Table 3: Post-translational modification coordinates criteria for comparison. It compares the value of a PTM coordinate of an input Proteoform with the value of a PTM coordinate in a reference proteoform. The letter k represents any positive integer.

Sensitivity analysis

In order to estimate the prevalence of missing annotation in Reactome, we evaluated the matching power of each matching type of PathwayMatcher using a reference list of 116,258 phosphosites obtained from Ochoa *et al.* [13]. Each phosphosite was transformed into a proteoform which had the same protein accession and a single PTM at the given site. The PTM accession number 00046, 00047, or 00048 was used if the phosphorylated amino acid reported was a serine, a threonine, or a tyrosine, respectively. Each of the proteoforms with a single phosphorylation was matched against all proteoforms available in Reactome using PathwayMatcher. The share of phosphosites yielding a match for each matching type is available in

Table 2.

Subsequently, we evaluated the robustness of each matching type by selecting sets of proteoforms from Reactome, altering them, and matching them back.

First, we selected the proteins which had multiple proteoforms with at least one PTM (1,364 proteins). Then, we gathered all those post-translationally modified proteoforms and altered them: (1) for the proteoforms with one or more PTMs, the type of the first PTM was replaced by "00000" and modification sites were increased by five positions; (2) for the proteoforms with two or more PTMs, the site of the second PTM was moved as well.

Then, we took 10 samples of 300 altered proteoforms and matched them to proteoforms in Reactome using PathwayMatcher. For each matching type we calculated the percentage proteoforms in the sample that matched any proteoform in the database.

The results for all ten samples are shown in **Figure 7**, where we split the matching the original sample proteoforms and other candidate proteoforms.

Mapping omics data to pathways

The input is mapped to proteins or proteoforms to find the reactions where the input entities are participants (Error! Reference source not found.). The input is mapped to proteins when data types without PTMs or specific translation products are specified; otherwise a mapping to proteoforms is used. When one type of data yields multiple results due to ambiguity, e.g. a SNP or peptide mapping multiple proteins, all the possibilities are included in the search entities.

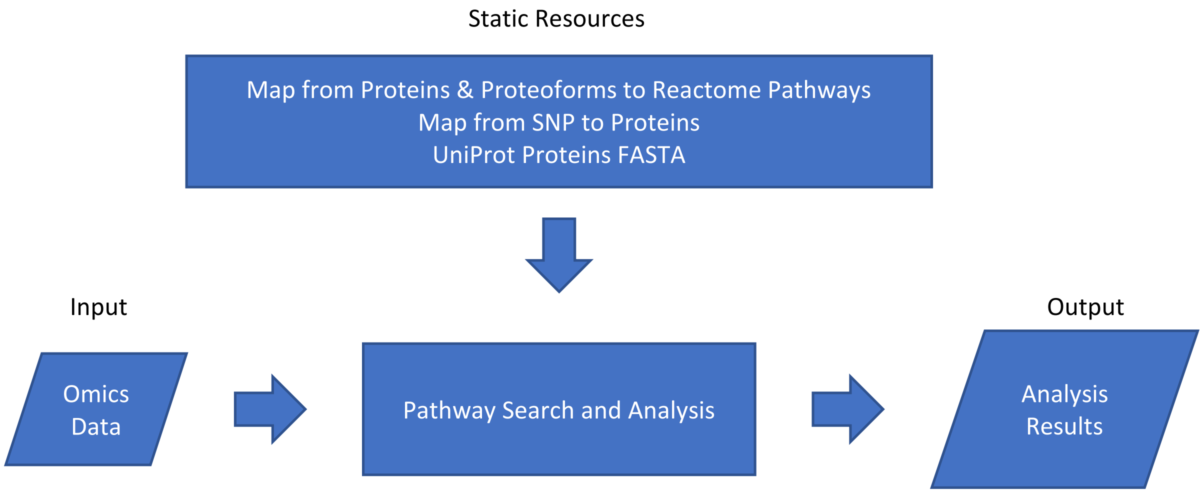

Figure 11: PathwayMatcher general overview. The program takes the user input in the form of omics data files and the reference pathways from the database as input. It then executes the search and analysis algorithm to create a resulting list of output files.

When a list of SNPs is provided, mapping from the Ensembl Variant Effect Predictor (VEP) [6] is used to find the possibly affected proteins. When peptides are provided, their sequence is mapped to UniProt protein identifiers [7] using PeptideMapper [8] and possible proteoforms are constructed. When proteins or proteoforms are available, PathwayMatcher maps them to reactions and pathways using data structures embedded in the PathwayMatcher jar file. These data structures are extracted from the Reactome Neo4j graph database[19] and serialized. All mapping files are available in a dedicated repository: github.com/PathwayAnalysisPlatform/MappingFiles.

In addition, we made it possible for the user to generate new mapping files as detailed in the PathwayMatcher repository (github.com/PathwayAnalysisPlatform/PathwayMatcher/tree/master/src/main/java/extractor). PathwayMatcher can then be executed with the new set of mapping files as provided by the user.

Over-representation analysis

The matching of each entity to a given pathway is modelled as a Bernoulli trial with two possible outcomes: success or failure, depending on whether the protein or proteoform is a participant of a reaction in the pathway. Trials are considered independent from each other, meaning that the outcome of previous trials does not affect the next. Finally, the probability of success is calculated by the proportion of choosing a protein in a pathway over the total number of possible proteins, therefore the probability is constant over all trials.

First, we search all the input entities (proteins or proteoforms) across all the pathways and count how many of them were found in each pathway. The number of entities found in a pathway is taken as the number of successful trials. Then, with the binomial probability distribution, we calculate how likely it would be to get a result equal to or more extreme than the current result (the same number or more proteins or proteoforms in the pathway), given that the input (proteins or proteoforms) were randomly selected [10].

This is done using the cumulative distribution function for the binomial distribution, which calculates the probability of getting at most k successes out of n trials, with a probability $p \in [0,1]$, where X is a random variable following the binomial distribution, as detailed in **Equation 1**.

$$F(k, n, p) = \Pr(X \leq k) = \sum_{i=0}^{\mathrm{k}} \binom{n}{i} p^i (1-p)^{n-i} \quad (1)$$

For each pathway, p is set to the ratio between the number of total proteins or proteoforms in the pathway and the total possible entities in the database, n is the number of proteins or proteoforms in the input sample, k is the number of proteins successfully mapped in the pathway, X is the number of entities found in the current pathway after the search.

Finally, given that the p-value requires the calculation of the probability of an equal or more extreme result, we use the complement of **Equation 1** to calculate the probability of getting at least k successful trials out of n as stated in **Equation 2**.

$$\Pr(\mathrm{X} \geq \mathrm{k}) = 1 - \Pr(\mathrm{X} \leq \mathrm{k} - 1) \quad (2)$$

The calculations for proteins or proteoforms are similar but are performed separately depending on the input. If the input consists of protein accessions, the number of participants is calculated by only considering proteins. On the other hand, for the proteoform input, the number of entities in the pathways and the database are the participant proteoforms.

Performance Benchmark

The performance of PathwayMatcher was evaluated using data sets of different sizes obtained from sampling publicly available resources:

- Proteins: human complement of the UniProtKB/Swiss-Prot database (release 2017_10).
- Peptides: ProteomeTools [20] as available in PRIDE [21], dataset PXD004732, release date 23/01/2017.
- Genetic variants: variants from the human assembly GRCh37.p13.
- Proteoforms: annotated proteoforms in Reactome Graph database version 62.

Performance testing was done using a standard desktop computer (Intel® Core™ i7-6600U CPU @ 2.60GHz with 2 cores using 64-bit Windows 10 with Java SE 1.8.0_144 on SSD). Details and code are available at

github.com/PathwayAnalysisPlatform/PathwayMatcher/wiki/Performance

Metrics and Figures

The metrics presented in this manuscript were obtained by querying the Reactome graph database directly [19]. The queries used can be found in the online documentation at:

github.com/PathwayAnalysisPlatform/PathwayMatcher/wiki/queries

The figures in this manuscript were built in R version 3.4.1 (2017-06-30) - "Single Candle" (r-project.org) using the following packages: ggplot2, ggrepel, igraph, scico, grid, purr, dplyr, graphlayouts, and gtable. The R scripts used to build the figures are available in the tool repository at:

github.com/PathwayAnalysisPlatform/PathwayMatcher_Publication/tree/master/R

Availability of supporting source code and requirements

Project name: PathwayMatcher

Project home page: github.com/PathwayAnalysisPlatform/PathwayMatcher

Operating system(s): Platform independent

Programming language: Java

Other requirements:

License: Apache 2.0

RRID: SCR_016759

Availability of Supporting Data

Snapshots of our code and other supporting data are available in the *GigaScience* repository, GigaDB [22].

Declarations

List of abbreviations

PTM: Post-translational modification

Ethics approval and consent to participate

Not applicable

Consent for publication

Not applicable

Competing interests

The authors declare that they have no competing interests

Funding

LFHS, SJ, PRN, and MV are supported by the European Research Council and the Research Council of Norway. BB, CH, and HB are supported by the Bergen Research Foundation. HB is also supported by the Research Council of Norway. LFHS, SJ, PRN, and MV are supported by the

European Research Council and by the Research Council of Norway. This work has been supported by National Institutes of Health BD2K grant (U54 GM114833) and National Human Genome Research Institute at the National Institutes of Health Reactome grant (U41 HG003751).

Authors' contributions

LFHS did most of the programming, testing, documentation, and wrote the manuscript. BB and CH contributed with programming, testing, documentation, ideas, and manuscript writing. AF, SJ, and PRN contributed with ideas and manuscript writing. HB contributed with ideas, supervised the work, and wrote the manuscript. HH contributed with project design, manuscript writing, and supervised the work. MV contributed with project design, programming, documentation, testing, supervised the work, and wrote the manuscript. All authors participated in the preparation of the manuscript.

Acknowledgements

The authors thank the Reactome curators for their massive curation effort and the guidance in interpreting the annotations. The authors thank the Galaxy community, and especially Dr. Björn Grüning, for their indefectible support.

References

1. Smith LM, Kelleher NL and The Consortium for Top Down P. Proteoform: a single term describing protein complexity. Nature methods. 2013;10 3:186-7. doi:10.1038/nmeth.2369.
2. Aebersold R, Agar JN, Amster IJ, Baker MS, Bertozzi CR, Boja ES, et al. How many human proteoforms are there? Nature Chemical Biology. 2018;14:206. doi:10.1038/nchembio.2576.
3. Seet BT, Dikic I, Zhou M-M and Pawson T. Reading protein modifications with interaction domains. Nature Reviews Molecular Cell Biology. 2006;7:473. doi:10.1038/nrm1960.
4. Menche J, Sharma A, Kitsak M, Ghiassian SD, Vidal M, Loscalzo J, et al. Uncovering disease-disease relationships through the incomplete interactome. Science. 2015;347 6224.
5. Fabregat A, Jupe S, Matthews L, Sidiropoulos K, Gillespie M, Garapati P, et al. The Reactome Pathway Knowledgebase. Nucleic acids research. 2018;46 D1:D649-d55. doi:10.1093/nar/gkx1132.
6. McLaren W, Gil L, Hunt SE, Riat HS, Ritchie GR, Thormann A, et al. The Ensembl Variant Effect Predictor. Genome Biol. 2016;17 1:122. doi:10.1186/s13059-016-0974-4.
7. The UniProt C. UniProt: the universal protein knowledgebase. Nucleic acids research. 2017;45 D1:D158-D69. doi:10.1093/nar/gkw1099.
8. Kopczynski D, Barsnes H, Njolstad PR, Sickmann A, Vaudel M and Ahrends R. PeptideMapper: efficient and versatile amino acid sequence and tag mapping. Bioinformatics. 2017;33 13:2042-4. doi:10.1093/bioinformatics/btx122.
9. Nesvizhskii AI and Aebersold R. Interpretation of shotgun proteomic data: the protein inference problem. Molecular & cellular proteomics : MCP. 2005;4 10:1419-40. doi:10.1074/mcp.R500012-MCP200.
10. García-Campos MA, Espinal-Enríquez J and Hernández-Lemus E. Pathway Analysis: State of the Art. Frontiers in Physiology. 2015;6 383 doi:10.3389/fphys.2015.00383.
11. Benjamini Y and Hochberg Y. Controlling the false discovery rate: a practical and powerful approach to multiple testing. Journal of the royal statistical society Series B (Methodological). 1995:289-300.
12. Schaffer LV, Millikin RJ, Miller RM, Anderson LC, Fellers RT, Ge Y, et al. Identification and Quantification of Proteoforms by Mass Spectrometry. Proteomics. 2019;19 10:1800361. doi:10.1002/pmic.201800361.
13. Ochoa D, Jarnuczak AF, Gehre M, Soucheray M, Kleefeldt AA, Vieitez C, et al. The functional landscape of the human phosphoproteome. bioRxiv. 2019:541656. doi:10.1101/541656.
14. Grüning B, Dale R, Sjödin A, Chapman BA, Rowe J, Tomkins-Tinch CH, et al. Bioconda: sustainable and comprehensive software distribution for the life sciences. Nature Methods. 2018;15 7:475-6. doi:10.1038/s41592-018-0046-7.
15. Afgan E, Baker D, Batut B, van den Beek M, Bouvier D, Čech M, et al. The Galaxy platform for accessible, reproducible and collaborative biomedical analyses: 2018 update. Nucleic acids research. 2018;46 W1:W537-W44. doi:10.1093/nar/gky379.
16. Blankenberg D, Von Kuster G, Bouvier E, Baker D, Afgan E, Stoler N, et al. Dissemination of scientific software with Galaxy ToolShed. Genome Biology. 2014;15 2:403. doi:10.1186/gb4161.
17. Montecchi-Palazzi L, Beavis R, Binz PA, Chalkley RJ, Cottrell J, Creasy D, et al. The PSI-MOD community standard for representation of protein modification data. Nat Biotechnol. 2008;26 8:864-6. doi:10.1038/nbt0808-864.
18. Natale DA, Arighi CN, Blake JA, Bona J, Chen C, Chen SC, et al. Protein Ontology (PRO): enhancing and scaling up the representation of protein entities. Nucleic acids research. 2017;45 D1:D339-d46. doi:10.1093/nar/gkw1075.
19. Fabregat A, Korninger F, Viteri G, Sidiropoulos K, Marin-Garcia P, Ping P, et al. Reactome graph database: Efficient access to complex pathway data. PLoS Comput Biol. 2018;14 1:e1005968. doi:10.1371/journal.pcbi.1005968.

20. Zolg DP, Wilhelm M, Schnatbaum K, Zerweck J, Knaute T, Delanghe B, et al. Building ProteomeTools based on a complete synthetic human proteome. Nature Methods. 2017;14:259. doi:10.1038/nmeth.4153.
21. Vizcaíno JA, Csordas A, del-Toro N, Dianes JA, Griss J, Lavidas I, et al. 2016 update of the PRIDE database and its related tools. Nucleic acids research. 2016;44 D1:D447-D56. doi:10.1093/nar/gkv1145.

22. Hernández Sánchez LF; Burger B; Horro C; Fabregat A; Johansson S; Njølstad PR; Barsnes H; Hermjakob H; Vaudel M: Supporting data for "PathwayMatcher: proteoform-centric network construction enables fine-granularity multi-omics pathway mapping" GigaScience Database. 2019. http://dx.doi.org/10.5524/100621

Answer to reviewers
Click here to access/download;Personal Cover;PathwayMatcher_Response_to_Reviewers_31.05.19.pdf

Reviewer reports:

Reviewer #1:

general review

The manuscript presents a software that extends beyond existing query methods for biological pathway databases in that it allows querying for specific proteoforms of a protein instead of only the consensus protein entry. It establishes different matching setups for proteoforms with varying strictness, describes the developed software and provides some basic characterization of how proteoform identifier queries can have an increased specificity compared to protein or gene identifier queries.

With the description of a new software tool and the augmented data base it uses, this manuscript is a good fit for publication in Giga Science.
The software and the respective data are available with an Apache license and are mostly well-documented in the manuscript and in a repository wiki. Code and data for the figures generated for the manuscript are available in the same repository.

With the two major questions below addressed, I see the minimum standards of reporting fulfilled and have no objections to publication.

Answer: We have carefully examined all comments and corrected our work accordingly. We are convinced that the software, documentation, and manuscript were greatly improved thanks to the reviewer's comments. We would therefore like to express our gratitude for this outstanding review.

requested revisions for publication

The following two main questions should in my opinion be addressed before publication. Below come further smaller comments, spotted errors and recommendations regarding the software, the data and the manuscript text itself.

## extended description of Extractor

The abstract states:
Based on the Reactome knowledgebase, we built a network of protein-protein interactions accounting for the documented isoform and modification statuses of proteins.

To me, this indicates that this generated network is a major part of the innovation presented in this manuscript. The data availability and method description requirements of Giga Science would in my opinion therefore require a description of what the respective Extractor tool does both in the manuscript here and in the README of the repository for its code (<https://github.com/PathwayAnalysisPlatform/Extractor>).

I would especially welcome a description of which exact resources are used to construct this network, and how it is constructed--i.e. what is matched to what. From the Extractor repository, it looks to me, as though data is extracted from the Ensembl Variant Effect Predictor (vep), ProteomeTools (peptides), PSIMOD and Reactome (neo4j). Are these all used to create a single network? Which versions of each data base were used in the current version of PathwayMatcher?

In connection to this Extractor point, please also see the recommendation for separation of data and code in the `data` section below.

Answer: We thank the reviewer for this suggestion. We agree that the manuscript was lacking details on the Extractor, and as the reviewer points out here and in the data section, our architecture was not efficient. We have therefore refactored our repositories entirely so that the organization is cleaner and the system easier to maintain. Notably, the code of the different modules, including Extractor, is now integrated into the PathwayMatcher repository. The structure of the application is now described in the wiki, with specific readme files for the different modules:

github.com/PathwayAnalysisPlatform/PathwayMatcher/tree/master/src/main/java/extractor/
github.com/PathwayAnalysisPlatform/PathwayMatcher/tree/master/src/main/java/model/
github.com/PathwayAnalysisPlatform/PathwayMatcher/tree/master/src/main/java/methods/
github.com/PathwayAnalysisPlatform/PathwayMatcher

The reviewer is correct that we use third-party tools and resources for the creation of the network and to allow the matching of different types of omics data. For the sake of ease of installation, portability, and performance, these third-party tools are not used when running PathwayMatcher, but static mappings are created by the extractor module at every release. We have extended the manuscript and documentation to clarify and better detail our usage of third-party tools and resources.

## decreased sensitivity?

While the manuscript clearly makes the point that using proteoform queries will improve specificity of the results, by narrowing down on fewer pathways and interactions than protein / gene queries would, it lacks a test and discussion of sensitivity. My main question would be:

Will using the proteoform query result in missing some potential pathways for lack of proper proteoform annotation to date?
This boils down to: Will available proteoforms of a gene always recreate all the interactions reported for that gene? Or asked the other way around: Are there genes where (a lot of or certain) interactions are only annotated for the main gene identifier, but not annotated for any of its reported proteoforms, while there are proteoforms reported?
I think that this could mostly be addressed by characterizing the current proteoform annotation status of the underlying Reactome data

base, e.g. answering questions like: Do genes with few annotated proteoforms have lots of gene-centric annotations that are not annotated to a specific proteoform? Does this number decrease with more proteoforms annotated? Here, both summary statistics and individual show-cases would be helpful, along the lines of what the manuscript nicely does for specificity.

Answer: The reviewer is correct that the sensitivity of the search is decreased when proteoform annotation is mismatching or missing. The annotation can be incomplete or inaccurate in the reference database, but also in the data, for example with bottom-up proteomics data. In some cases, one can speculate that the loss of sensitivity might even shadow the gain in specificity.

The reviewer is correct in that the gene-centric representation encompass all proteoform-centric edges, without the distinction of proteoform-proteoform interaction between proteoforms from the same gene. In contrast, the proteoform-centric representation contains the gene-centric network, but with more details. As a consequence, it is possible to build the gene-centric network from the proteoform-centric representation, but not the other way around.
To give the user more flexibility, we implemented many ways of tuning the matching: by relaxing proteoform matching tolerances, the user can increase sensitivity at the cost of specificity, up to the extreme case of matching by accession, where there is no loss of sensitivity but no gain in specificity. We anticipate that users will use different stringencies in proteoform matching based on the type of data queried, ranging from exact proteoform matching to gene matching, hence balancing specificity and sensitivity. It will even be possible to do differential analyses using different levels of stringencies in matching.

To highlight this, we conducted a sensitivity and specificity analysis and included all results in the manuscript. As suggested by the reviewer, we used individual show-cases (namely Insulin and *MAP3K7*) as well as summary statistics. We also use a recently published meta-analysis of phosphoproteomics data representing over 100,000 phosphosites. We are convinced that the results of these analyses greatly improved the text and will be valuable to the users when tuning PathwayMatcher. We would therefore like to thank the reviewer for this challenging but very useful comment.

software

installation

It is very much appreciated, that various options for installation and usage are offered, that all aim at a simple installation and reproducible usage. I have explicitly tried out the installation via bioconda and can confirm that it installs seamlessly.

Answer: We thank the reviewer for underlying our efforts in integrating our software in multiple bioinformatic environments. This has been greatly enabled by the Galaxy community who deserves acknowledgement for their indefectible support.

documentation

Both the installation process and the usage are well documented, with the documentation Wiki linked to directly in the main README of the software repository. Example data for all possible input data is provided. As proteoform input is a unique feature of PathwayMatcher, I used this as a general test case for trying out the software.

The software worked well and produced the described outputs. One thing I was missing in documentation were suggestions on how to visualise and / or analyse the graph files that are an optional output. Here, I could imagine both a general pointer to software and / or a pointer to scripts used in the manuscript or elsewhere.

Answer: We thank the reviewer for this suggestion. Links to follow-up analysis tools (Cytoscape, IGraph), and to the scripts used to generate the examples featured in the paper have been added to the documentation:
github.com/PathwayAnalysisPlatform/PathwayMatcher/wiki/Protein-connection-graph#visualization-and-follow-up-analysis
github.com/PathwayAnalysisPlatform/PathwayMatcher_Publication/tree/master/R
github.com/PathwayAnalysisPlatform/PathwayMatcher/wiki/queries

command-line interface

The command-line interface provides a useful help message and provides standard flags like `--version`. Some minor things I have stumbled upon where I would suggest future improvements--but which I would not make a requirement for publication--are:
* It seems like not all command line options are displayed in the `--help` output, e.g. I found the hidden `--version` tag.
The options for help and version are now visible.
* It would be useful to have the help message display the defaults for command line arguments. I came across this for the match type, when using the proteoform.

* It would be useful to have a quick description of the output files generated in the help message, so not to have to refer to the wiki for that.
* It would be useful to be able to specify the names of individual output files for easier pipeline integration of PathwayMatcher, where usually input and output files have to be named explicitly. The `--output` path option makes this possible, but individual options for the file names with the current values as defaults would in my opinion increase usability.
* Instead of one command for all possible input types, I would recommend using different subcommands instead of a command line argument for input type. This would allow for different interfaces for different formats, as e.g. for proteoform input you have to specify the matching type,

whereas other input types don't need this. So a usage could look like something along the lines of `pathwaymatcher match-proteoforms <options>` or `java -jar PathwayMatcher.jar match-proteforms <options>`.

From the above points, it seems like the currently used CLI library is probably not the best choice. As I am not a Java programmer, I am only guessing here and cannot recommend a better command line interface library, but maybe this stackexchange thread is useful: <https://softwarerecs.stackexchange.com/questions/16450/what-library-should-i-use-for-handling-cli-arguments-for-my-java-program>

Answer:
The options for help and version are now visible and can be executed with the short ("-v") and long ("--version") arguments. The default values for range and matchType are shown in the help text. The other arguments have no default value, but the user is now required to provide the values in order to execute. We added a brief description of the output files in the help text and what each command does.

We replaced the command line interface library from Apache CLI to Picocli. The "inputType" parameter was removed in favor of the subcommands interface provided by the new library. We also made it possible for the user to name the output files produced by a command execution using a common prefix, which allows using the same output folder for different runs without overwriting of the results.

We thank the reviewer for these suggestions which greatly simplify the usage of the tool.

## code

Upon a quick glance by a non-Java coder, the code looks well organised and seems to contain extensive tests for the different possible input formats, which is very much appreciated. The modules in the separate repositories (Model, Method and Extractor) all still lack a useful README file, which would help grasping how they work together, but the code itself contains useful comments.

Answer: We thank the reviewer for his appreciation of our effort to abide by programming good practices, and for taking the time to dive in our code. As suggested, a README.md file has been added to the Extractor, Model and Methods modules. As detailed in our answer to the first comment, the code architecture has been refactored and better documented.

data

Example input data is available for all possible input types and output formats are well described in the documentation. The data base needed for mapping inputs to Reactome pathways is provided with the executable and is thus directly available.

The last point, while facilitating accessibility, is also a point of

criticism for me. With the data base included in the main software repository, including multiple versions of it in the `.git` history, the repository currently has a size of 2 GB and will drastically increase in size with every new version of the data base generated--which will become necessary with every new version of the Reactome data base that someone wants to use with PathwayMatcher. Also, there will be differences between the version numbers of the software and the Reactome data base mapping packaged with it and with the current setup it will not be clear to users which is which--from what I gather, I cannot currently query the command-line tool for the Reactome data base used. I would therefore recommend separating out the network generated with Extractor from the software repository, and distributing it separately (e.g. via GigaDB: <http://gigadb.org/>, Open Science Framework: <https://osf.io/> or something similar, e.g. check via: <https://www.re3data.org>). This will reduce the repo size drastically, from currently above 2 GB to probably a couple of MB, and will then allow for a separate versioning of the software and versions of the network generated from different versions of Reactome. To remove large files from git history, e.g. consider the respective GitHub tutorial: <https://help.github.com/articles/removing-sensitive-data-from-a-repository/>

A further reduction in repo size could be achieved by also separating out the manuscript (including code for plots) from the software code into a separate repository. As the manuscript and associated code will not change further after publication, such a repository would not change further, whereas the software will live on.

Answer: Once again we thank the reviewer for very relevant suggestions on how to organize our codebase. We have now refactored our repositories and better described the structure in the documentation: all code necessary to build and use the network are now in the same repository (github.com/PathwayAnalysisPlatform/PathwayMatcher), and all large files are now in a separate repository (github.com/PathwayAnalysisPlatform/MappingFiles), as well as all code and resources used for the paper (github.com/PathwayAnalysisPlatform/PathwayMatcher_Publication). As a result, the main repository is much smaller, and the cloning of the repository takes considerably less space and time.

The version of Reactome and all third-party resources are available from the command line and displayed in the command line help.
A set of compressed mapping files are still included in PathwayMatcher to ensure that it can be run upon download, and to facilitate integration in docker and Galaxy. Now, it is further possible for the user to create the static mapping files within the Extractor (github.com/PathwayAnalysisPlatform/PathwayMatcher/tree/master/src/main/java/extractor/#running-extractor), this allows setting the version of the database locally. We added a parameter for the path to the mapping files to be used in the pathway analysis. We however anticipate that this functionality will be used by expert users only.

manuscript / text comments

Findings

Page 5, line 10: The self-citation [1] does not provide support for the statement in the previous sentence, that proteins through biochemical reactions form pathways that interact to form a biological network. However, this statement is so basic that a citation might not be necessary, at all.

Answer: The citation has been removed. However, we disagree with the reviewer that the citation does not support the sentence since the structure of the network formed by pathways and its complexity are precisely the object of this study.

Page 7, Line 53 (Figure 2):
It is not immediately apparent, that counts are cumulative, as this is only mentioned later in the caption. I would suggest the following two minor changes:

* amend the y-axis label to read: cumulative # publications
* amend the caption start to read: The cumulative number of publications

Answer: The y-axis title has been renamed and the caption updated accordingly.

Page 8, Line 50 (Figure 3):
Two minor changes I would like to suggest:

* correct the caption start from protein to proteoform, to read:
Gene-centric versus proteoform-centric representation
* Gene symbols should always be italicized, while protein symbols should always be just plain formatting. Currently, this is not used systematically in this caption, while the main text seems to be fine.

Answer: This has been corrected. Since the legends are in italic, gene names are switched back to roman there, as normally done for italics within italics.

Page 12, Figure 5, panels C and D:
How can a ratio of degrees which are all positive become negative? Or are the ratio values in the inset log10-transformed, like the values in panel D? This should be noted in the axis labelling and the figure caption. To make the panels more accessible, I wouldn't log-transform the values, but only the axes -- as it is done in panel B. In this case, the tick mark labels of ratios in the C inset would correspond to values found in the main text and the tick mark labels in D would correspond to the

degree values in panel C. In addition, the colour scale used in panel D, could also be used in the inset in panel C, to further highlight the correspondence.

Answer: The reviewer is correct that the ratio in C is log-transformed and we apologize that this figure was not correctly annotated and described. This has now been corrected. We have also now use the same scaling, representation, and coloring throughout the elements of the panel. We thank the reviewer for these suggestions that greatly improved the figure.

## Methods

Proteoform matching

The description of the proteoform matching types was very hard to follow, especially the part starting page 19, line 5 and running until page 22, line 1. I would remove redundancies between the different matching types, to make this section more readable. In order to make every definition only once, the following reasoning flow seems the most straightforward to me:
1. matching of UniProt accessions
2. matching of isoform specifiers (if isoform doesn't exist in Reactome, shouldn't it match the unmodified one as a default? should there be a mode for that?)
3. PTM matching:
   1. coordinate matching
   2. type matching
4. explain the three non-strict matching types and that they can all be invoked with or without considering PTM type information
5. describe how the strict matching differs from the other matching types

Table 1: The input reference combinations 18-17, 9-13 and 17-13 do not add any information, I would remove them for a quicker overview and only keep the important corner cases. Also, Table 1 is not referenced in the text, but probably should be in the description of PTM coordinate matching.

Answer: We thank the reviewer for suggestions on how to improve this section. It has been rewritten accordingly.

Mapping omics data to pathways

Page 23, line 50: The link in parentheses suggests to be the source of the Reactome database, while this is only a tool to download it -- as

described at: <https://reactome.org/dev/graph-database>. I would prefer having the proper citation of the database here (currently reference [22])

Tables 2 and 3: These do not really add to the text, so I would skip them altogether or reduce them to something like 2-3 entries each.

Answer: This link has been replaced. The tables have been relocated to a summary statistics wiki page and are referred to in the results section.
github.com/PathwayAnalysisPlatform/PathwayMatcher/wiki/Summary-statistics

## References

* Reference 13 is a duplicate of 6.
* Reference 14 is a duplicate of 3.

Answer: This has been corrected.

Reviewer #2:

The manuscript entitled "PathwayMatcher: proteoform-centric network construction enables fine-granularity multi-omics pathway mapping" by Sánchez et. al describes a new paradigm to build networks for human biomedical data based on proteoforms including PTMs rather than centering on gene. Developed algorithm relies on Reactome knowledgebase database for proteoform interactions. This manuscript has originality and covers an interesting topic for multi-omics field. I have no doubts that this application will be of great interest for OMICS users. It is important to highlight this review is from the viewpoint of a potential user, since I am a researcher that works with proteomics rather than an expert in application developer. Therefore, I lack the expertise to evaluate the technical algorism issues and I hope other revi-ewers with this expertise will bring more valuable suggestions on this matter. Regarding the use of PathwayMatcher, the Galaxy version seems user friendly and intuitive. However, in my experience was not straightforward when I tried. It is essential to have a better tutorial for users to get the output results as reactions & pathways, over-representation and network view as illustrated in figure 4 of the manuscript. In case users have to login to have full access, this information should be clear. In addition, the local installation shows a major concern. Even though I had installed the Java as suggested in the website instructions I could not execute the jar file. The error was "could not find or load main class". Since, this local installation is an option in additional to the galaxy version, it would be helpful to have a better description in the website regarding possible

troubleshoots to guide new users.

Answer: We thank the reviewer for this positive assessment for our work. We have now extended the documentation, and notably added more details on how to get started and how to work with the output. We apologize for the issues with the local installation, the command line should run as simply as in Bioconda or Galaxy. We have corrected potential issues and extended the documentation to prevent problems with the local installation.

The suggestions pointed by this reviewer were here in order to improve users' accessibility since I believe and hope that PathwayMatcher will be widely used in OMICS field.
Minor points:
-> This reviewer believes that authors used the term "isoform" sometimes to do not overwrite the correct term "proteoform". However, I strongly suggest using only proteoform throughout the manuscript since it is the most acceptable term nowadays.

Answer: We agree with the reviewer that isoforms and proteoforms are two different concepts and have thoroughly checked the manuscript that the wording is correct.

-> I suggest the author to include a zoom-in on fig 3B to highlight the proteoforms (including PTMs) in the red nodes regarding TP53 gene.

Answer: We thank the reviewer for this suggestion, the different nodes are now annotated as suggested.

-> There are several proteoforms that does not have the interaction information. How often will be PathwayMatcher updating the database? Will it be based on Reactome update? Please indicate in the manuscript.

Answer: PathwayMatcher is updated at every release of Reactome, bug fix, and new feature implementation.
Furthermore, the code has now been extended so that users can generate the mapping files for PathwayMatcher from a specific version of Reactome. Then the program can be executed with an extra parameter stating the location of the self-generated mapping files. We expect this feature to be of interest to expert users. Instructions on how to do this are given in the wiki: github.com/PathwayAnalysisPlatform/PathwayMatcher/tree/master/src/main/java/extractor/#running-extractor

-> For consistency, the MOD number for all modifications represented in Fig. 8 (x-axis) should be included.

Answer: This has been fixed.

-> The phrase "PathwayMatcher is developed to be a hypothesis generation

tool, helping to navigating large datasets and guide experiments. It is not a validation or mechanism inference tool" written in Methods section should be included in the main body text as many readers may first recognize this as a potential tool to understand biological mechanisms.

Answer: We agree with the reviewer and apologize for this inconsistency in the manuscript. This consideration has now been moved to the discussion and made more prominent.

Personal Cover Click here to access/download;Personal Cover;Cover Letter.pdf

Dear Editor, Dear Nicole,

We hereby would like to submit the revised version of our manuscript entitled *PathwayMatcher: proteoform-centric network construction enables fine-granularity multi-omics pathway mapping* for publication in GigaScience.

We sincerely apologize for the delay of our revision. Although the initial decision on the manuscript was *minor revision*, correctly answering all comments of reviewer 1 was a serious piece of work: (1) to answer their first main comment, we refactored our code repository entirely and better documented the different modules of the software, resulting in a clearer structure; (2) to answer their second main comment, we conducted a thorough sensitivity and robustness analysis using a recently published phosphoproteome and a synthetic data set of proteoforms derived from Reactome. In addition, we corrected our code, documentation, and manuscript in detail based on all other comments from both reviewers.

Importantly, the new analyses strengthen the results we initially reported and do not alter the usage of the tool for the end user. Overall, our software and manuscript have been greatly improved by the review process. We would therefore like to express our gratitude to the reviewers for their outstanding work and hope that you will find our revised version acceptable for publication.

On behalf of the authors,

Luis Francisco Hernández Sánchez and Marc Vaudel
