# Supplementary material for: PathwayMatcher: proteoform-centric network construction enables fine-granularity multiomics pathway mapping
Source: Gigascience. 2019 Jul 30;8(8):giz088. doi: 10.1093/gigascience/giz088 (PMC6667378; doi:10.1093/gigascience/giz088)
Supplement: giz088_Response_to_Reviewer_Comments_Original_Submission [file giz088_response_to_reviewer_comments_original_submission.pdf]

Author's Response To Reviewer Comments

Close

Reviewer reports:
Reviewer #1:

general review

The manuscript presents a software that extends beyond existing query methods for biological pathway databases in that it allows querying for specific proteoforms of a protein instead of only the consensus protein entry. It establishes different matching setups for proteoforms with varying strictness, describes the developed software and provides some basic characterization of how proteoform identifier queries can have an increased specificity compared to protein or gene identifier queries.

With the description of a new software tool and the augmented data base it uses, this manuscript is a good fit for publication in Giga Science.
The software and the respective data are available with an Apache license and are mostly well-documented in the manuscript and in a repository wiki. Code and data for the figures generated for the manuscript are available in the same repository.

With the two major questions below addressed, I see the minimum standards of reporting fulfilled and have no objections to publication.

Answer: We have carefully examined all comments and corrected our work accordingly. We are convinced that the software, documentation, and manuscript were greatly improved thanks to the reviewer's comments. We would therefore like to express our gratitude for this outstanding review.

requested revisions for publication

The following two main questions should in my opinion be addressed before publication. Below come further smaller comments, spotted errors and recommendations regarding the software, the data and the manuscript text itself.

extended description of Extractor

The abstract states:
Based on the Reactome knowledgebase, we built a network of protein-protein interactions accounting for the documented isoform and modification statuses of proteins.

To me, this indicates that this generated network is a major part of the innovation presented in this manuscript. The data availability and method description requirements of Giga Science would in my opinion therefore require a description of what the respective Extractor tool does both in the manuscript here and in the README of the repository for its code ().

I would especially welcome a description of which exact resources are used to construct this network, and how it is constructed--i.e. what is matched to what. From the Extractor repository, it looks to me, as though data is extracted from the Ensembl Variant Effect Predictor (vep), ProteomeTools (peptides), PSIMOD and Reactome (neo4j). Are these all used to create a single network? Which versions of each data base

were used in the current version of PathwayMatcher?

In connection to this Extractor point, please also see the
recommendation for separation of data and code in the `data` section below.

Answer: We thank the reviewer for this suggestion. We agree that the manuscript was lacking details on the Extractor, and as the reviewer points out here and in the data section, our architecture was not efficient. We have therefore refactored our repositories entirely so that the organization is cleaner and the system easier to maintain. Notably, the code of the different modules, including Extractor, is now integrated into the PathwayMatcher repository. The structure of the application is now described in the wiki, with specific readme files for the different modules:

github.com/PathwayAnalysisPlatform/PathwayMatcher/tree/master/src/main/java/extractor/
github.com/PathwayAnalysisPlatform/PathwayMatcher/tree/master/src/main/java/model/
github.com/PathwayAnalysisPlatform/PathwayMatcher/tree/master/src/main/java/methods/
github.com/PathwayAnalysisPlatform/PathwayMatcher

The reviewer is correct that we use third-party tools and resources for the creation of the network and to allow the matching of different types of omics data. For the sake of ease of installation, portability, and performance, these third-party tools are not used when running PathwayMatcher, but static mappings are created by the extractor module at every release. We have extended the manuscript and documentation to clarify and better detail our usage of third-party tools and resources.

decreased sensitivity?

While the manuscript clearly makes the point that using proteoform
queries will improve specificity of the results, by narrowing down on
fewer pathways and interactions than protein / gene queries would, it
lacks a test and discussion of sensitivity. My main question would be:

Will using the proteoform query result in missing some potential
pathways for lack of proper proteoform annotation to date?
This boils down to: Will available proteoforms of a gene always recreate
all the interactions reported for that gene? Or asked the other way
around: Are there genes where (a lot of or certain) interactions are
only annotated for the main gene identifier, but not annotated for any
of its reported proteoforms, while there are proteoforms reported?
I think that this could mostly be addressed by characterizing the
current proteoform annotation status of the underlying Reactome data
base, e.g. answering questions like: Do genes with few annotated
proteoforms have lots of gene-centric annotations that are not annotated
to a specific proteoform? Does this number decrease with more
proteoforms annotated? Here, both summary statistics and individual
show-cases would be helpful, along the lines of what the manuscript
nicely does for specificity.

Answer: The reviewer is correct that the sensitivity of the search is decreased when proteoform annotation is mismatching or missing. The annotation can be incomplete or inaccurate in the reference database, but also in the data, for example with bottom-up proteomics data. In some cases, one can speculate that the loss of sensitivity might even shadow the gain in specificity.

The reviewer is correct in that the gene-centric representation encompass all proteoform-centric edges, without the distinction of proteoform-proteoform interaction between proteoforms from the same gene. In contrast, the proteoform-centric representation contains the gene-centric network, but with more details. As a consequence, it is possible to build the gene-centric network from the proteoform-centric representation, but not the other way around.
To give the user more flexibility, we implemented many ways of tuning the matching: by relaxing proteoform matching tolerances, the user can increase sensitivity at the cost of specificity, up to the extreme case of matching by accession, where there is no loss of sensitivity but no gain in specificity. We anticipate that users will use different stringencies in proteoform matching based on the type of data queried, ranging from exact proteoform matching to gene matching, hence balancing specificity and

sensitivity. It will even be possible to do differential analyses using different levels of stringencies in matching.

To highlight this, we conducted a sensitivity and specificity analysis and included all results in the manuscript. As suggested by the reviewer, we used individual show-cases (namely Insulin and MAP3K7) as well as summary statistics. We also use a recently published meta-analysis of phosphoproteomics data representing over 100,000 phosphosites. We are convinced that the results of these analyses greatly improved the text and will be valuable to the users when tuning PathwayMatcher. We would therefore like to thank the reviewer for this challenging but very useful comment.

software

installation

It is very much appreciated, that various options for installation and usage are offered, that all aim at a simple installation and reproducible usage. I have explicitly tried out the installation via bioconda and can confirm that it installs seamlessly.

Answer: We thank the reviewer for underlying our efforts in integrating our software in multiple bioinformatic environments. This has been greatly enabled by the Galaxy community who deserves acknowledgement for their indefectible support.

documentation

Both the installation process and the usage are well documented, with the documentation Wiki linked to directly in the main README of the software repository. Example data for all possible input data is provided. As proteoform input is a unique feature of PathwayMatcher, I used this as a general test case for trying out the software.

The software worked well and produced the described outputs. One thing I was missing in documentation were suggestions on how to visualise and / or analyse the graph files that are an optional output. Here, I could imagine both a general pointer to software and / or a pointer to scripts used in the manuscript or elsewhere.

Answer: We thank the reviewer for this suggestion. Links to follow-up analysis tools (Cytoscape, IGraph), and to the scripts used to generate the examples featured in the paper have been added to the documentation:
github.com/PathwayAnalysisPlatform/PathwayMatcher/wiki/Protein-connection-graph#visualization-and-follow-up-analysis
github.com/PathwayAnalysisPlatform/PathwayMatcher_Publication/tree/master/R
github.com/PathwayAnalysisPlatform/PathwayMatcher/wiki/queries

command-line interface

The command-line interface provides a useful help message and provides standard flags like `--version`. Some minor things I have stumbled upon where I would suggest future improvements--but which I would not make a requirement for publication--are:
* It seems like not all command line options are displayed in the `--help` output, e.g. I found the hidden `--version` tag.
The options for help and version are now visible.
* It would be useful to have the help message display the defaults for command line arguments. I came across this for the match type, when using the proteoform.

* It would be useful to have a quick description of the output files generated in the help message, so not to have to refer to the wiki for that.

* It would be useful to be able to specify the names of individual output files for easier pipeline integration of PathwayMatcher, where usually input and output files have to be named explicitly. The `--output` path option makes this possible, but individual options for the file names with the current values as defaults would in my opinion increase usability.
* Instead of one command for all possible input types, I would recommend using different subcommands instead of a command line argument for input type. This would allow for different interfaces for different formats, as e.g. for proteoform input you have to specify the matching type, whereas other input types don't need this. So a usage could look like something along the lines of `pathwaymatcher match-proteoforms ` or `java -jar PathwayMatcher.jar match-proteforms `.

From the above points, it seems like the currently used CLI library is probably not the best choice. As I am not a Java programmer, I am only guessing here and cannot recommend a better command line interface library, but maybe this stackexchange thread is useful:

Answer:
The options for help and version are now visible and can be executed with the short ("-v") and long ("--version") arguments. The default values for range and matchType are shown in the help text. The other arguments have no default value, but the user is now required to provide the values in order to execute. We added a brief description of the output files in the help text and what each command does.

We replaced the command line interface library from Apache CLI to Picocli. The "inputType" parameter was removed in favor of the subcommands interface provided by the new library. We also made it possible for the user to name the output files produced by a command execution using a common prefix, which allows using the same output folder for different runs without overwriting of the results.

We thank the reviewer for these suggestions which greatly simplify the usage of the tool.

code

Upon a quick glance by a non-Java coder, the code looks well organised and seems to contain extensive tests for the different possible input formats, which is very much appreciated. The modules in the separate repositories (Model, Method and Extractor) all still lack a useful README file, which would help grasping how they work together, but the code itself contains useful comments.

Answer: We thank the reviewer for his appreciation of our effort to abide by programming good practices, and for taking the time to dive in our code. As suggested, a README.md file has been added to the Extractor, Model and Methods modules. As detailed in our answer to the first comment, the code architecture has been refactored and better documented.

data

Example input data is available for all possible input types and output formats are well described in the documentation. The data base needed for mapping inputs to Reactome pathways is provided with the executable and is thus directly available.

The last point, while facilitating accessibility, is also a point of criticism for me. With the data base included in the main software repository, including multiple versions of it in the `.git` history, the repository currently has a size of 2 GB and will drastically increase in size with every new version of the data base generated--which will become necessary with every new version of the Reactome data base that someone wants to use with PathwayMatcher. Also, there will be

differences between the version numbers of the software and the Reactome data base mapping packaged with it and with the current setup it will not be clear to users which is which--from what I gather, I cannot currently query the command-line tool for the Reactome data base used. I would therefore recommend separating out the network generated with Extractor from the software repository, and distributing it separately (e.g. via GigaDB: , Open Science Framework:
or something similar, e.g. check via:
). This will reduce the repo size drastically, from currently above 2 GB to probably a couple of MB, and will then allow for a separate versioning of the software and versions of the network generated from different versions of Reactome. To remove large files from git history, e.g. consider the respective GitHub tutorial:

A further reduction in repo size could be achieved by also separating out the manuscript (including code for plots) from the software code into a separate repository. As the manuscript and associated code will not change further after publication, such a repository would not change further, whereas the software will live on.

Answer: Once again we thank the reviewer for very relevant suggestions on how to organize our codebase. We have now refactored our repositories and better described the structure in the documentation: all code necessary to build and use the network are now in the same repository (github.com/PathwayAnalysisPlatform/PathwayMatcher), and all large files are now in a separate repository (github.com/PathwayAnalysisPlatform/MappingFiles), as well as all code and resources used for the paper (github.com/PathwayAnalysisPlatform/PathwayMatcher_Publication). As a result, the main repository is much smaller, and the cloning of the repository takes considerably less space and time.

The version of Reactome and all third-party resources are available from the command line and displayed in the command line help.
A set of compressed mapping files are still included in PathwayMatcher to ensure that it can be run upon download, and to facilitate integration in docker and Galaxy. Now, it is further possible for the user to create the static mapping files within the Extractor (github.com/PathwayAnalysisPlatform/PathwayMatcher/tree/master/src/main/java/extractor/#running-extractor), this allows setting the version of the database locally. We added a parameter for the path to the mapping files to be used in the pathway analysis. We however anticipate that this functionality will be used by expert users only.

manuscript / text comments

Findings

Page 5, line 10: The self-citation [1] does not provide support for the statement in the previous sentence, that proteins through biochemical reactions form pathways that interact to form a biological network. However, this statement is so basic that a citation might not be necessary, at all.

Answer: The citation has been removed. However, we disagree with the reviewer that the citation does not support the sentence since the structure of the network formed by pathways and its complexity are precisely the object of this study.

Page 7, Line 53 (Figure 2):
It is not immediately apparent, that counts are cumulative, as this is only mentioned later in the caption. I would suggest the following two minor changes:

* amend the y-axis label to read: cumulative # publications

* amend the caption start to read: The cumulative number of publications

Answer: The y-axis title has been renamed and the caption updated accordingly.

Page 8, Line 50 (Figure 3):
Two minor changes I would like to suggest:

* correct the caption start from protein to proteoform, to read:
Gene-centric versus proteoform-centric representation
* Gene symbols should always be italicized, while protein symbols should always be just plain formatting. Currently, this is not used systematically in this caption, while the main text seems to be fine.

Answer: This has been corrected. Since the legends are in italic, gene names are switched back to roman there, as normally done for italics within italics.

Page 12, Figure 5, panels C and D:
How can a ratio of degrees which are all positive become negative? Or are the ratio values in the inset log10-transformed, like the values in panel D? This should be noted in the axis labelling and the figure caption. To make the panels more accessible, I wouldn't log-transform the values, but only the axes -- as it is done in panel B. In this case, the tick mark labels of ratios in the C inset would correspond to values found in the main text and the tick mark labels in D would correspond to the degree values in panel C. In addition, the colour scale used in panel D, could also be used in the inset in panel C, to further highlight the correspondence.

Answer: The reviewer is correct that the ratio in C is log-transformed and we apologize that this figure was not correctly annotated and described. This has now been corrected. We have also now use the same scaling, representation, and coloring throughout the elements of the panel. We thank the reviewer for these suggestions that greatly improved the figure.

Methods

Proteoform matching

The description of the proteoform matching types was very hard to follow, especially the part starting page 19, line 5 and running until page 22, line 1. I would remove redundancies between the different matching types, to make this section more readable. In order to make every definition only once, the following reasoning flow seems the most straightforward to me:
1. matching of UniProt accessions
2. matching of isoform specifiers (if isoform doesn't exist in Reactome, shouldn't it match the unmodified one as a default? should there be a mode for that?)
3. PTM matching:
1. coordinate matching
2. type matching
4. explain the three non-strict matching types and that they can all be invoked with or without considering PTM type information
5. describe how the strict matching differs from the other matching types

Table 1: The input reference combinations 18-17, 9-13 and 17-13 do not add any information, I would remove them for a quicker overview and only keep the important corner cases. Also, Table 1 is not referenced in the

text, but probably should be in the description of PTM coordinate matching.

Answer: We thank the reviewer for suggestions on how to improve this section. It has been rewritten accordingly.

Mapping omics data to pathways

Page 23, line 50: The link in parentheses suggests to be the source of the Reactome database, while this is only a tool to download it -- as described at: . I would prefer having the proper citation of the database here (currently reference [22])

Tables 2 and 3: These do not really add to the text, so I would skip them altogether or reduce them to something like 2-3 entries each.

Answer: This link has been replaced. The tables have been relocated to a summary statistics wiki page and are referred to in the results section.
github.com/PathwayAnalysisPlatform/PathwayMatcher/wiki/Summary-statistics

References

* Reference 13 is a duplicate of 6.
* Reference 14 is a duplicate of 3.

Answer: This has been corrected.

Reviewer #2:
The manuscript entitled "PathwayMatcher: proteoform-centric network construction enables fine-granularity multi-omics pathway mapping" by Sánchez et. al describes a new paradigm to build networks for human biomedical data based on proteoforms including PTMs rather than centering on gene. Developed algorithm relies on Reactome knowledgebase database for proteoform interactions. This manuscript has originality and covers an interesting topic for multi-omics field. I have no doubts that this application will be of great interest for OMICS users. It is important to highlight this review is from the viewpoint of a potential user, since I am a researcher that works with proteomics rather than an expert in application developer. Therefore, I lack the expertise to evaluate the technical algorism issues and I hope other revi-ewers with this expertise will bring more valuable suggestions on this matter. Regarding the use of PathwayMatcher, the Galaxy version seems user friendly and intuitive. However, in my experience was not straightforward when I tried. It is essential to have a better tutorial for users to get the output results as reactions & pathways, over-representation and network view as illustrated in figure 4 of the manuscript. In case users have to login to have full access, this information should be clear. In addition, the local installation shows a major concern. Even though I had installed the Java as suggested in the website instructions I could not execute the jar file. The error was "could not find or load main class". Since, this local installation is an option in additional to the galaxy version, it would be helpful to have a better description in the website regarding possible troubleshoots to guide new users.

Answer: We thank the reviewer for this positive assessment for our work. We have now extended the documentation, and notably added more details on how to get started and how to work with the output. We apologize for the issues with the local installation, the command line should run as simply as in

Bioconda or Galaxy. We have corrected potential issues and extended the documentation to prevent problems with the local installation.

The suggestions pointed by this reviewer were here in order to improve users' accessibility since I believe and hope that PathwayMatcher will be widely used in OMICS field.
Minor points:
-> This reviewer believes that authors used the term "isoform" sometimes to do not overwrite the correct term "proteoform". However, I strongly suggest using only proteoform throughout the manuscript since it is the most acceptable term nowadays.

Answer: We agree with the reviewer that isoforms and proteoforms are two different concepts and have thoroughly checked the manuscript that the wording is correct.

-> I suggest the author to include a zoom-in on fig 3B to highlight the proteoforms (including PTMs) in the red nodes regarding TP53 gene.

Answer: We thank the reviewer for this suggestion, the different nodes are now annotated as suggested.

-> There are several proteoforms that does not have the interaction information. How often will be PathwayMatcher updating the database? Will it be based on Reactome update? Please indicate in the manuscript.

Answer: PathwayMatcher is updated at every release of Reactome, bug fix, and new feature implementation.
Furthermore, the code has now been extended so that users can generate the mapping files for PathwayMatcher from a specific version of Reactome. Then the program can be executed with an extra parameter stating the location of the self-generated mapping files. We expect this feature to be of interest to expert users. Instructions on how to do this are given in the wiki: github.com/PathwayAnalysisPlatform/PathwayMatcher/tree/master/src/main/java/extractor/#running-extractor

-> For consistency, the MOD number for all modifications represented in Fig. 8 (x-axis) should be included.

Answer: This has been fixed.

-> The phrase "PathwayMatcher is developed to be a hypothesis generation tool, helping to navigating large datasets and guide experiments. It is not a validation or mechanism inference tool" written in Methods section should be included in the main body text as many readers may first recognize this as a potential tool to understand biological mechanisms.

Answer: We agree with the reviewer and apologize for this inconsistency in the manuscript. This consideration has now been moved to the discussion and made more prominent.

Close
