# Supplementary material for: PathwayMatcher: proteoform-centric network construction enables fine-granularity multiomics pathway mapping
Source: Gigascience. 2019 Jul 30;8(8):giz088. doi: 10.1093/gigascience/giz088 (PMC6667378; doi:10.1093/gigascience/giz088)
Supplement: giz088_Reviewer_1_Report_Original_Submission — David Lahnemann -- 1/10/2019 Reviewed [file giz088_reviewer_1_report_original_submission.pdf]

Reviewer Report

Title: PathwayMatcher: proteoform-centric network construction enables fine-granularity multi-omics pathway mapping

Version: Original Submission **Date:** 1/10/2019

Reviewer name: David Lahnemann

Reviewer Comments to Author:

general review
The manuscript presents a software that extends beyond existing query methods for biological pathway databases in that it allows querying for specific proteoforms of a protein instead of only the consensus protein entry. It establishes different matching setups for proteoforms with varying strictness, describes the developed software and provides some basic characterization of how proteoform identifier queries can have an increased specificity compared to protein or gene identifier queries.
With the description of a new software tool and the augmented data base it uses, this manuscript is a good fit for publication in Giga Science. The software and the respective data are available with an Apache license and are mostly well-documented in the manuscript and in a repository wiki. Code and data for the figures generated for the manuscript are available in the same repository.
With the two major questions below addressed, I see the minimum standards of reporting fulfilled and have no objections to publication.
requested revisions for publication
The following two main questions should in my opinion be addressed before publication. Below come further smaller comments, spotted errors and recommendations regarding the software, the data and the manuscript text itself.
extended description of Extractor
The abstract states:
Based on the Reactome knowledgebase, we built a network of protein-protein interactions accounting for the documented isoform and modification statuses of proteins.
To me, this indicates that this generated network is a major part of the innovation presented in this manuscript. The data availability and method description requirements of Giga Science would in my opinion therefore require a description of what the respective Extractor tool does both in the manuscript here and in the README of the repository for its code (<https://github.com/PathwayAnalysisPlatform/Extractor>).
I would especially welcome a description of which exact resources are used to construct this network, and how it is constructed--i.e. what is matched to what. From the Extractor repository, it looks to me, as though data is extracted from the Ensembl Variant Effect Predictor (vep), ProteomeTools (peptides), PSIMOD and Reactome (neo4j). Are these all used to create a single network? Which versions of each data base were used in the current version of PathwayMatcher?
In connection to this Extractor point, please also see the recommendation for separation of data and code in the `data` section below.

decreased sensitivity?
While the manuscript clearly makes the point that using proteoform queries will improve specificity of the results, by narrowing down on fewer pathways and interactions than protein / gene queries would, it lacks a test and discussion of sensitivity. My main question would be:
Will using the proteoform query result in missing some potential pathways for lack of proper proteoform annotation to date?
This boils down to: Will available proteoforms of a gene always recreate all the interactions reported for that gene? Or asked the other way around: Are there genes where (a lot of or certain) interactions are only annotated for the main gene identifier, but not annotated for any of its reported proteoforms, while there are proteoforms reported?
I think that this could mostly be addressed by characterizing the current proteoform annotation status of the underlying Reactome data base, e.g. answering questions like: Do genes with few annotated proteoforms have lots of gene-centric annotations that are not annotated to a specific proteoform? Does this number descrease with more proteoforms annotated? Here, both summary statistics and individual show-cases would be helpful, along the lines of what the manuscript nicely does for specificity.
software
installation
It is very much appreciated, that various options for installation and usage are offered, that all aim at a simple installation and reproducible usage. I have explicitly tried out the installation via bioconda and can confirm that it installs seamlessly.
documentation
Both the installation process and the usage are well documented, with the documentation Wiki linked to directly in the main README of the software repository. Example data for all possible input data is provided. As proteoform input is a unique feature of PathwayMatcher, I used this as a general test case for trying out the software.
The software worked well and produced the described outputs. One thing I was missing in documentation were suggestions on how to visualise and / or analyse the graph files that are an optional output. Here, I could imagine both a general pointer to software and / or a pointer to scripts used in the manuscript or elsewhere.
command-line interface
The command-line interface provides a useful help message and provides standard flags like `--version`. Some minor things I have stumbled upon where I would suggest future improvements--but which I would not make a requirement for publication--are:
* It seems like not all command line options are displayed in the `--help` output, e.g. I found the hidden `--version` tag.
* It would be useful to have the help message display the defaults for command line arguments. I came across this for the match type, when using the proteoform.
* It would be useful to have a quick description of the output files generated in the help message, so not to have to refer to the wiki for that.
* It would be useful to be able to specify the names of individual output files for easier pipeline integration of PathwayMatcher, where usually input and output files have to be named explicitly. The `--

output` path option makes this possible, but individual options for the file names with the current values as defaults would in my opinion increase usability.
* Instead of one command for all possible input types, I would recommend using different subcommands instead of a command line argument for input type. This would allow for different interfaces for different formats, as e.g. for proteoform input you have to specify the matching type, whereas other input types don't need this. So a usage could look like something along the lines of `pathwaymatcher match-proteoforms <options>` or `java -jar PathwayMatcher.jar match-proteforms <options>`.
From the above points, it seems like the currently used CLI library is probably not the best choice. As I am not a Java programmer, I am only guessing here and cannot recommend a better command line interface library, but maybe this stackexchange thread is useful: <https://softwarerecs.stackexchange.com/questions/16450/what-library-should-i-use-for-handling-cli-arguments-for-my-java-program>
code
Upon a quick glance by a non-Java coder, the code looks well organised and seems to contain extensive tests for the different possible input formats, which is very much appreciated. The modules in the separate repositories (Model, Method and Extractor) all still lack a useful README file, which would help grasping how they work together, but the code itself contains useful comments.
data
Example input data is available for all possible input types and output formats are well described in the documentation. The data base needed for mapping inputs to Reactome pathways is provided with the executable and is thus directly available.
The last point, while facilitating accessibility, is also a point of criticism for me. With the data base included in the main software repository, including multiple versions of it in the `.git` history, the repository currently has a size of 2 GB and will drastically increase in size with every new version of the data base generated--which will become necessary with every new version of the Reactome data base that someone wants to use with PathwayMatcher. Also, there will be differences between the version numbers of the software and the Reactome data base mapping packaged with it and with the current setup it will not be clear to users which is which--from what I gather, I cannot currently query the command-line tool for the Reactome data base used.
I would therefore recommend separating out the network generated with Extractor from the software repository, and distributing it separately (e.g. via GigaDB: <http://gigadb.org/>, Open Science Framework: <https://osf.io/> or something similar, e.g. check via: <https://www.re3data.org>). This will reduce the repo size drastically, from currently above 2 GB to probably a couple of MB, and will then allow for a separate versioning of the software and versions of the network generated from different versions of Reactome. To remove large files from git history, e.g. consider the respective GitHub tutorial:
<https://help.github.com/articles/removing-sensitive-data-from-a-repository/>
A further reduction in repo size could be achieved by also separating out the manuscript (including code for plots) from the software code into a separate repository. As the manuscript and associated code will not change further after publication, such a repository would not change further, whereas the software will live on.

manuscript / text comments
Findings
Page 5, line 10: The self-citation [1] does not provide support for the statement in the previous sentence, that proteins through biochemical reactions form pathways that interact to form a biological network. However, this statement is so basic that a citation might not be necessary, at all.
Page 7, Line 53 (Figure 2):
It is not immediately apparent, that counts are cumulative, as this is only mentioned later in the caption. I would suggest the following two minor changes:
* amend the y-axis label to read: cumulative # publications
* amend the caption start to read: The cumulative number of publications
Page 8, Line 50 (Figure 3):
Two minor changes I would like to suggest:
* correct the caption start from protein to proteoform, to read: Gene-centric versus proteoform-centric representation
* Gene symbols should always be italicized, while protein symbols should always be just plain formatting. Currently, this is not used systematically in this caption, while the main text seems to be fine.
Page 12, Figure 5, panels C and D:
How can a ratio of degrees which are all positive become negative? Or are the ratio values in the inset log10-transformed, like the values in panel D? This should be noted in the axis labelling and the figure caption.
To make the panels more accessible, I wouldn't log-transform the values, but only the axes -- as it is done in panel B. In this case, the tick mark labels of ratios in the C inset would correspond to values found in the main text and the tick mark labels in D would correspond to the degree values in panel C. In addition, the colour scale used in panel D, could also be used in the inset in panel C, to further highlight the correspondence.
Methods
Proteoform matching
The description of the proteoform matching types was very hard to follow, especially the part starting page 19, line 5 and running until page 22, line 1. I would remove redundancies between the different matching types, to make this section more readable. In order to make every definition only once, the following reasoning flow seems the most straightforward to me:
1. matching of UniProt accessions
2. matching of isoform specifiers (if isoform doesn't exist in Reactome, shouldn't it match the unmodified one as a default? should there be a mode for that?)
3. PTM matching:
1. coordinate matching
2. type matching
4. explain the three non-strict matching types and that they can all be invoked with or without considering PTM type information
5. describe how the strict matching differs from the other matching types
Table 1: The input reference combinations 18-17, 9-13 and 17-13 do not add any information, I would

remove them for a quicker overview and only keep the important corner cases. Also, Table 1 is not referenced in the text, but probably should be in the description of PTM coordinate matching.
Mapping omics data to pathways
Page 23, line 50: The link in parentheses suggests to be the source of the Reactome database, while this is only a tool to download it -- as described at: <https://reactome.org/dev/graph-database>. I would prefer having the proper citation of the database here (currently reference [22])
Tables 2 and 3: These do not really add to the text, so I would skip them altogether or reduce them to something like 2-3 entries each.
References
* Reference 13 is a duplicate of 6.
* Reference 14 is a duplicate of 3.

Level of Interest

Please indicate how interesting you found the manuscript: Choose an item.

Quality of Written English

Please indicate the quality of language in the manuscript: Choose an item.

Declaration of Competing Interests

Please complete a declaration of competing interests, considering the following questions:

- Have you in the past five years received reimbursements, fees, funding, or salary from an organisation that may in any way gain or lose financially from the publication of this manuscript, either now or in the future?
- Do you hold any stocks or shares in an organisation that may in any way gain or lose financially from the publication of this manuscript, either now or in the future?
- Do you hold or are you currently applying for any patents relating to the content of the manuscript?
- Have you received reimbursements, fees, funding, or salary from an organization that holds or has applied for patents relating to the content of the manuscript?
- Do you have any other financial competing interests?
- Do you have any non-financial competing interests in relation to this paper?

If you can answer no to all of the above, write 'I declare that I have no competing interests' below. If your reply is yes to any, please give details below.

I declare that I have no competing interests

I agree to the open peer review policy of the journal. I understand that my name will be included on my report to the authors and, if the manuscript is accepted for publication, my named report including any attachments I upload will be posted on the website along with the authors' responses. I agree for my report to be made available under an Open Access Creative Commons CC-BY license (http://creativecommons.org/licenses/by/4.0/). I understand that any comments which I do not wish to be included in my named report can be included as confidential comments to the editors, which will not be published.

I agree to the open peer review policy of the journal

To further support our reviewers, we have joined with Publons, where you can gain additional credit to further highlight your hard work (see: https://publons.com/journal/530/gigascience). On publication of this paper, your review will be automatically added to Publons, you can then choose whether or not to claim your Publons credit. I understand this statement.

Yes Choose an item.
