# Supplementary material for: PathwayMatcher: proteoform-centric network construction enables fine-granularity multiomics pathway mapping
Source: Gigascience. 2019 Jul 30;8(8):giz088. doi: 10.1093/gigascience/giz088 (PMC6667378; doi:10.1093/gigascience/giz088)
Supplement: giz088_Reviewer_2_Report_Original_Submission — Marcella Nunes Melo-Braga, PhD -- 2/21/2019 Reviewed [file giz088_reviewer_2_report_original_submission.pdf]

Reviewer Report

Title: PathwayMatcher: proteoform-centric network construction enables fine-granularity multi-omics pathway mapping

Version: Original Submission **Date:** 2/21/2019

Reviewer name: Marcella Nunes Melo-Braga, PhD

Reviewer Comments to Author:

The manuscript entitled "PathwayMatcher: proteoform-centric network construction enables fine-granularity multi-omics pathway mapping" by SÃ¡nches et. al describes a new paradigm to build networks for human biomedical data based on proteoforms including PTMs rather than centering on gene. Developed algorithm relies on Reactome knowledgebase database for proteoform interactions. This manuscript has originality and covers an interesting topic for multi-omics field. I have no doubts that this application will be of great interest for OMICS users.
It is important to highlight this review is from the viewpoint of a potential user, since I am a researcher that works with proteomics rather than an expert in application developer. Therefore, I lack the expertise to evaluate the technical algorism issues and I hope other revi-ewers with this expertise will bring more valuable suggestions on this matter.
Regarding the use of PathwayMatcher, the Galaxy version seems user friendly and intuitive. However, in my experience was not straightforward when I tried. It is essential to have a better tutorial for users to get the output results as reactions & pathways, over-representation and network view as illustrated in figure 4 of the manuscript. In case users have to login to have full access, this information should be clear. In addition, the local installation shows a major concern. Even though I had installed the Java as suggested in the website instructions I could not execute the jar file. The error was "could not find or load main class". Since, this local installation is an option in additional to the galaxy version, it would be helpful to have a better description in the website regarding possible troubleshoots to guide new users.
The suggestions pointed by this reviewer were here in order to improve users' accessibility since I believe and hope that PathwayMatcher will be widely used in OMICS field.
Minor points:
-> This reviewer believes that authors used the term "isoform" sometimes to do not overwrite the correct term "proteoform". However, I strongly suggest using only proteoform throughout the manuscript since it is the most acceptable term nowadays.
-> I suggest the author to include a zoom-in on fig 3B to highlight the proteoforms (including PTMs) in the red nodes regarding TP53 gene.
-> There are several proteoforms that does not have the interaction information. How often will be PathwayMatcher updating the database? Will it be based on Reactome update? Please indicate in the manuscript.
-> For consistency, the MOD number for all modifications represented in Fig. 8 (x-axis) should be included.

-> The phrase "PathwayMatcher is developed to be a hypothesis generation tool, helping to navigating large datasets and guide experiments. It is not a validation or mechanism inference tool" written in Methods section should be included in the main body text as many readers may first recognize this as a potential tool to understand biological mechanisms.

Level of Interest

Please indicate how interesting you found the manuscript: Choose an item.

Quality of Written English

Please indicate the quality of language in the manuscript: Choose an item.

Declaration of Competing Interests

Please complete a declaration of competing interests, considering the following questions:

- Have you in the past five years received reimbursements, fees, funding, or salary from an organisation that may in any way gain or lose financially from the publication of this manuscript, either now or in the future?
- Do you hold any stocks or shares in an organisation that may in any way gain or lose financially from the publication of this manuscript, either now or in the future?
- Do you hold or are you currently applying for any patents relating to the content of the manuscript?
- Have you received reimbursements, fees, funding, or salary from an organization that holds or has applied for patents relating to the content of the manuscript?
- Do you have any other financial competing interests?
- Do you have any non-financial competing interests in relation to this paper?

If you can answer no to all of the above, write 'I declare that I have no competing interests' below. If your reply is yes to any, please give details below.

I declare that I have no competing interests

I agree to the open peer review policy of the journal. I understand that my name will be included on my report to the authors and, if the manuscript is accepted for publication, my named report including any attachments I upload will be posted on the website along with the authors' responses. I agree for my report to be made available under an Open Access Creative Commons CC-BY license (http://creativecommons.org/licenses/by/4.0/). I understand that any comments which I do not wish to be included in my named report can be included as confidential comments to the editors, which will not be published.

I agree to the open peer review policy of the journal

To further support our reviewers, we have joined with Publons, where you can gain additional credit to further highlight your hard work (see: https://publons.com/journal/530/gigascience). On publication of this paper, your review will be automatically added to Publons, you can then choose whether or not to claim your Publons credit. I understand this statement.

Yes Choose an item.
